# Molecular mechanisms underlying opportunistic seasonal reproduction in the male rodent pest *Arvicola terrestris scherman*

**DOI:** 10.1038/s41598-026-51017-9

**Published:** 2026-04-30

**Authors:** A. Chorfa, Y. Renaud, Y. Bidet, S. Viala, G. Marceau, S. Bravard, C. Damon-Soubeyrand, C. Martins, S. Gominard, J. R. Drevet, F. Saez

**Affiliations:** 1 EVALSEM, Faculté de Médecine, Université Clermont Auvergne, Clermont Auvergne Innovation, CRBC, GReD Institute, 28 Place Henri Dunant, Clermont-Ferrand, 63001 France; 2 Faculté de Médecine, GReD Institute, CNRS UMR 6293-Inserm U1103 - Université Clermont Auvergne, CRBC, 28, Place Henri Dunant, Clermont- Ferrand cedex, 63001 France; 3 Laboratoire d’Oncologie Moléculaire, Centre Jean Perrin, Imagerie Moléculaire et Stratégies Théranostiques, INSERM U1240 - Université Clermont Auvergne, 58 rue Montalembert, Clermont Ferrand, 63000 France; 4 FREDON Auvergne-Rhône-Alpes, délégation Auvergne, 16 rue Aimé RUDEL, Lempdes, 63370 France

**Keywords:** Spermatozoa, Testis, Seasonal breeding, Wild vole, Climate change, Ecology, Ecology, Physiology, Zoology

## Abstract

The fossorial vole *Arvicola terrestris scherman* (*ATS*) is a pest rodent in Central Europe that is subject to cyclical infestations, the frequency of which is steadily increasing in a global context of climate and environmental change. Due to the significant economic, agricultural, and sanitary issues associated with *ATS* outbreaks, research is being conducted to better understand the physiological, behavioral, and environmental characteristics of these phenomena. The work presented here consisted of comparing *ATS* males from summer and winter in terms of anatomical, systemic, and testicular gene expression parameters. Interestingly, two reproductive morphs of males with the same anatomical characteristics in terms of overall body weight and coat length were observed during the winter period. One morph is typical of this seasonal breeding species, showing significant winter regression of its reproductive organs, while the other morph is atypical, appearing to having preserved the male reproductive tissues and function. Histological and RNAseq analyses of the testicular tissue clearly define the distinct characteristics of these animals, confirming the maintenance of reproductive capacity in some winter males. Knowledge of these physiological changes is an important factor in defining relevant control strategies.

**Supplementary Information:**

The online version contains supplementary material available at 10.1038/s41598-026-51017-9.

## Introduction

For most seasonal mammals of the temperate zone, photoperiod detection together with temperature and food availability reliably predicts changes in foraging conditions and energy balance^1–3^. These environmental cues are key drivers of alternating periods of sexual activity and reproductive quiescence, as reproduction imposes substantial energy cost^4,5^. However, marked interspecific and interindividual variation exists in responsiveness to environmental parameters^6^. Such variability is particularly pronounced in small rodents, whose short generation facilitates rapid adaptive responses to environmental fluctuations through selection of “opportunistic” breeding strategies^3^.

*Arvicola terrestris scherman* (*ATS)* belongs to the *Cricetidae* family and is mainly found in mountainous regions of southern and central Europe, at altitudes between 800 m and 2,400 m. It represents a fossorial southwestern European phylogeographic variant of the water vole *Arvicola amphibius*^7^. *ATS* area of repartition includes temperate mid-mountain permanent meadows and orchards where it digs extensive burrow systems. Classified as a major agricultural pest in several European countries, *ATS* undergoes cyclic outbreaks^8^ that cause considerable economic damage and management challenges^9–11^. Comprehensive syntheses of its biology, ecology, and control strategies emphasize the complexity of its population regulation and the need to better understand the determinants of reproductive dynamics^12^. In France, understanding and predicting these outbreaks has been a long-lasting challenge involving several governmental regulatory agencies, researchers and field stakeholders.

In *Arvicolinae* rodents, reproductive timing is tightly linked to population dynamics, and even subtle modifications in the onset, duration, or intensity of reproductive activity may have major demographic consequences. This may partly explain the recurrent cyclic outbreaks of the fossorial vole *ATS*, reported across several European regions over the last decades^13^.

Long-term field studies based on capture–recapture approaches and demographic monitoring have described marked fluctuations in reproductive activity, population structure, and gonadal condition in this taxon^14–17^. Cyclic changes in population structure and breeding activity have also been documented in related taxa, including *Arvicola amphibius*^18^. Although these earlier works did not investigate molecular or gene expression mechanisms, they clearly demonstrated that reproductive activity in *ATS* and closely related forms can vary considerably across regions and years, including reports of winter breeding or extended reproductive periods in certain populations^19–22^. Continuous or nearly continuous breeding has been described in northwestern Iberian populations of *Arvicola scherman*, suggesting substantial ecological flexibility in reproductive scheduling^20,23,24^. Together, these studies highlight that the phenotypic expression of reproductive cycles—assessed through gonadal morphology, reproductive indices, and demographic parameters—may differ markedly depending on geographic and environmental context. Importantly, while demographic and morphological studies across Europe have described winter breeding, unconventional reproductive timing, or region-specific reproductive patterns, the physiological and molecular mechanisms underlying these phenotypic observations remain largely unexplored. The reproductive cycles documented at the population level ultimately represent the phenotypic outcome of underlying endocrine, cellular, and gene regulatory processes. In this sense, molecular analyses such as those presented here provide a mechanistic framework that may help explain previously reported variations in reproductive timing and intensity among European populations.

In an earlier study, we reported that *ATS* are classified as seasonal breeders and that photoperiod strongly regulates reproductive activity via modulation of the hypothalamo-pituitary-gonadal (HPG) axis^25^. Temperature and food availability were also suspected to modulate this photoperiodic control, allowing fine-tuning of the onset and termination of the reproductive season.

Within a research program aimed at developing alternate control strategies based on immunocontraception targeting male gamete proteins^26^, we conducted seasonal field collections of *ATS* males in the Puy de Sancy (Auvergne, France), one of the French hot spots of *ATS* outbreaks. Transcriptomic analysis of testes from winter and summer males was performed to evaluate seasonal expression patterns of candidate immunocontraceptive.

targets^26^. Unexpectedly, these analyses revealed that a significant proportion of winter males exhibited molecular signatures consistent with active reproductive function, suggesting the presence of an “opportunistic” breeding phenotype.

Here, we report that a non-negligible fraction of *ATS* males maintains reproductive capacities during winter, a season generally considered non-permissive for reproduction in temperate seasonal rodents. At the molecular level, these sexually active winter males display a distinctive transcriptomic profile more closely resembling that of fully reproductive spring males than that of reproductively quiescent winter conspecifics. Placed in the broader geographic and ecological framework provided by previous demographic and morphological studies across Europe, our results suggest that the winter breeding reported in some populations may rely on specific underlying molecular configurations. From a population dynamics perspective, the persistence of reproductively competent males during winter could accelerate population growth as soon as permissive conditions return, thereby contributing to the rapid amplification phase characteristic of *ATS* outbreaks.

## Materials & methods

### Animals

Male *Arvicola terrestris scherman* (*ATS*) were collected in natural pastures in the region of Puy-de-Dôme (France): Perpezat (45°68’33’’ N–2°78′33′’ E); Nébouzat (45°42’59’’N–2°54′19′’ E); Saint-Julien-Puy-Lavèze (45°39’58’’ N–2°40′25′’ E). Winter males were trapped in January and February and summer males in July and August (2019–2020). In the immediate vicinity of freshly made molehills, galleries were detected with a sounder and a trap (Topcat) was placed inside. Only animals of reproductive age, that is with a weight greater than 70 g, were kept alive and brought back to the animal facility the same day where they were housed in a controlled environment (23 °C, 12 h light/12 h dark). The *ATS* were fed *ad libitum* with carrots. All the following procedures were approved by the Auvergne Animal Experiment Ethics Committee (C2E2A) and the French Ministry for Research (APAFIS authorization # 10653-2017071016422159 v5). The study was designed, conducted, and reported in accordance with the ARRIVE guidelines (Animal Research: Reporting of In Vivo Experiments) and the EU Directive 2010/63/EU for animal experiments. The animals were anesthetized using 4% isoflurane (Isovet, France) in the inhaled air to perform blood sampling. They were then killed by cervical dislocation before tissue sampling.

### Tissue sampling and processing

After cervical dislocation, the testes, epididymides, seminal vesicles and the epididymal white adipose tissue were sampled and weighted. One caput epididymis and one testis *per* animal were fixed in 4% paraformaldehyde (Euromedex, France) for 24 h and 48 h, respectively. Tissues were then dehydrated in an increasing ethanol gradient and placed in Histoclear (HS200; National Diagnostics, USA) for 2 h before paraffin embedding. The other testis and epididymis were snap-frozen in liquid nitrogen and kept at -80 °C until further use.

### Histology, immunohistochemistry and immunofluorescence

Five µm thick paraffin sections of testis and epididymis were made and tissue sections were then deparaffinized by passage through Histoclear and progressively rehydrated in a decreasing ethanol gradient. Masson’s Trichrome staining was performed using an automated deparaffinization and staining machine (HMS 70, Microm) and the slides were finally mounted with Cytoseal (Thermo Fisher Scientific, USA). For immunohistochemistry and immunofluorescence, after deparaffinization, slides were rehydrated in PBS for 5 min at room temperature (RT), and endogenous peroxidases were inhibited with 0.3% H_2_O_2_ in PBS for 30 min at RT for the immunohistochemistry procedure. Tissue sections were then saturated for 1 h in PBS with 5% normal goat serum (Vector Laboratories, USA), and incubated overnight with the following primary antibodies: anti-SYCP3 (Abcam ab97672, 1/2000), anti-H4Ac (Millipore 06866, 1/2000), anti-ZBTB16 (R&D AF2944, 1/1000), anti-AMH (Abcam ab25542, 1/250), anti-GATA4 (Santa Cruz sc1237, 1/500) and anti-THRA (a Sertoli cell maturation marker, Abclonal A5592, 1/75) diluted in PBS with 2.5% normal goat serum. The slides were then rinsed three times 5 min in PBS at RT and incubated with either fluorescent secondary antibodies (1/500 in NGS 2.5%, conjugated to Alexa-fluor A488 or A555) or with biotinylated secondary antibodies (1/500 in NGS 2.5%). For immunohistochemistry, the revelation was performed by incubating the slides with streptavidin-HRP (1/500 in NGS 2.5%) and with the Novared revelation kit (Vector Laboratories). Finally, slides were counterstained with either 1 µg/ml Hoechst 33,342 (Sigma-Aldrich^®^), mounted in Mowiol™ (Electron Microscopy Science), or counterstained with hematoxylin staining and mounted in PBS-glycerol. They were then observed through a Zeiss Axioimager.M2 epifluorescence microscope, and analyzed using Zen 2.3 software (Zeiss).

### Blood biochemistry

Blood was collected at the time of tissue sampling, as a terminal sampling using intracardiac puncture under anesthesia. A maximum volume of 1 ml was collected. After collection, whole blood was centrifuged and the serum collected and stored at -80 °C until use. All concentrations were determined on an automated clinical chemistry analyzer (Dimension Vista 1500, Siemens Healthcare Diagnostics) according to the supplier’s recommendations and standard ISO15189. Free T3 and free T4 were measured by chemiluminescent immunoassay; LDL cholesterol, HDL cholesterol, total cholesterol and triglycerides by Trinder reaction.

### RNA-sequencing and analysis

Total mRNA was isolated from testicular frozen tissues using miRNeasy mini-Kit (Qiagen, France), with DNAse treatment. The quality and quantity of total RNAs were determined using 4150 TapeStation and RNA ScreenTapes (Agilent Technologies). cDNA libraries were prepared using KAPA mRNA HyperPrep Kit (Roche Diagnostics). This kit captures messenger RNA using magnetic oligo-dT beads and it fragments the material using heat and magnesium. Then first strand cDNA was synthetized using random priming and Illumina adapters were added by ligation. Library preparation was performed according to supplier recommendations and as previously described, using 1 µg of total RNA input to ensure high diversity. Indexed cDNA libraries were sequenced single end (1 × 75 bp reads) according to the standard Illumina protocol, using NextSeq 550 High Output v2 kit. An average of 20.2 million reads were generated *per* sample (with a minimum of 14.1 million).

Reads were filtered and trimmed to remove adapter-derived or low-quality bases using trim_galore tool v0.6.10 with parameter –stringency = 6 and FASTQC v0.12.1 was used to verify quality of the outputs. Illumina reads were aligned to the Microtus_ochrogaster reference genome (MicOch1) with Hisat2. Read counts were generated for each annotated gene using HTSeq-Count. Read normalization, variance estimation and pair-wise differential expression analysis with multiple testing corrections were conducted using the R Bioconductor DESeq2 package and RPKM (Reads Per Kilobase of exon per Megabase of library size) were computed during this process. PCA were generated using « PCAplot() » function of the DEseq2 package using only the 500 most highly variant genes. Venn diagrams were generated using “Venny” (https://bioinfogp.cnb.csic.es/tools/venny/). Gene set enrichment analyses were performed with R package “clusterProfiler” v4.10.1 using mm10 annotation database (org.Mm.eg.db). Statistical tests were realized with R or Graphpad PRISM 5.

### qRT-PCR

In order to better characterize the W^+^ phenotype, we performed molecular analyses on specific targets involved in testicular physiology regulation, using both qRT-PCR and immunofluorescence (see above). Five targets were selected, to study the different stages of spermatogenesis (*Zbtb16* and *Sycp3*), Sertoli cell function (*Amh* and *Gata4*) and the thyroid hormone signaling pathway, a known regulator of seasonal reproduction in rodents.

Total mRNAs from testis (see above for the extraction protocol) were reverse transcribed (1 µg) with Moloney leukemia virus reverse transcriptase and random hexamer primers (Promega) according to the manufacturer’s instructions. Quantitative PCR was performed with SYBR Premix Ex Taq II (TaKaRa Bio) on a LightCycler 480 II (Roche). Primer sequences are listed in supplementary Table 1. The relative accumulation level of each mRNA was normalized using actin as a standard, following the comparative Ct method (ΔΔCt method).

### Statistics

To determine whether differences were statistically significant, groups were compared using the non-parametric Kruskal-Wallis test, followed by Dunn’s Multiple Comparison Test. *p* < 0.05 was considered statistically significant. GraphPad PRISM 5 software was used to perform the statistical analysis and generate the associated graphs.

## Results

**Fig. 1:**
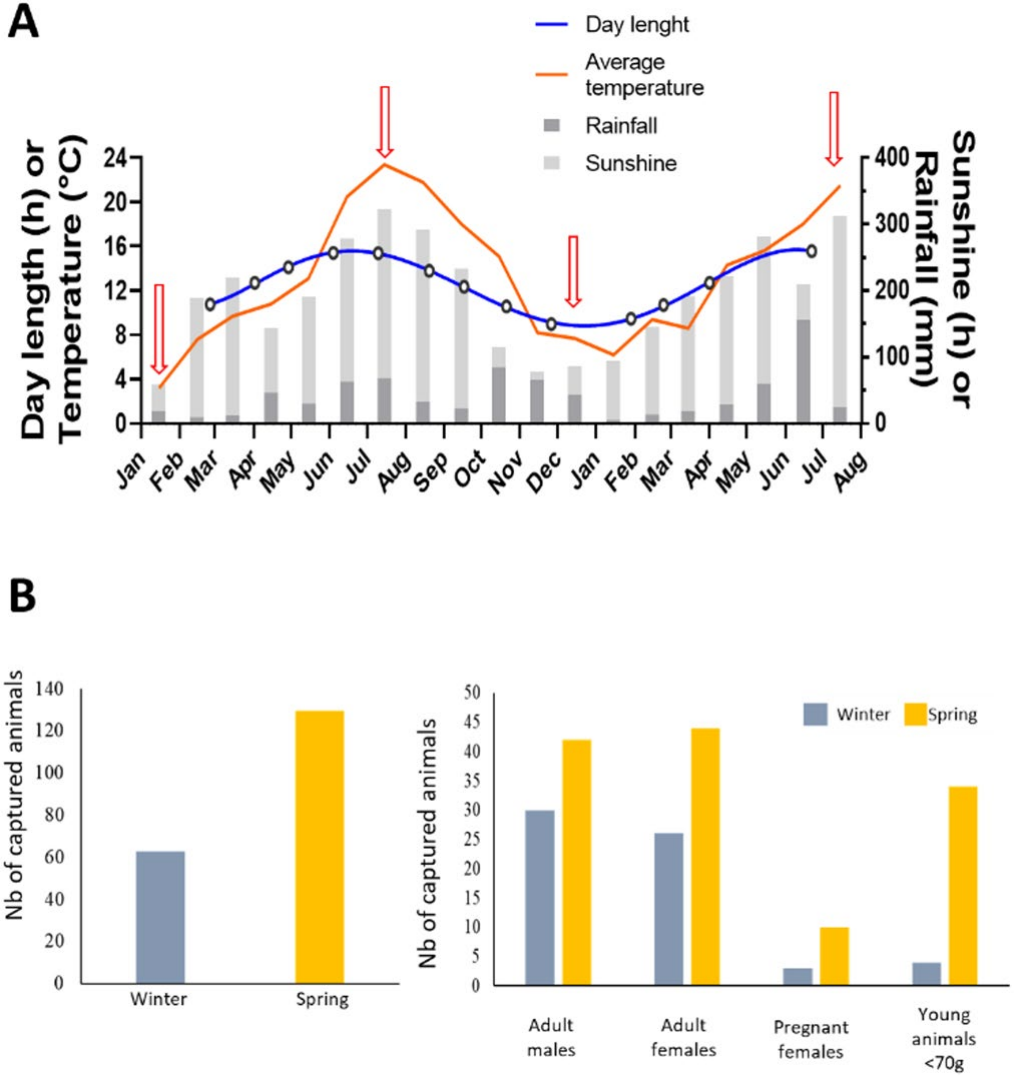
Seasonal variations and animal capture. (**A**) Representation of the climatic parameters (Day length, average temperature, rainfall and sunshine) in the area where the animals were captured, during the 2-year period of the study. (**B**) Details of the animal categories trapped in relation with the season (left panel) or with the age, sex and gestation status of the females (right panel).

**Fig. 2:**
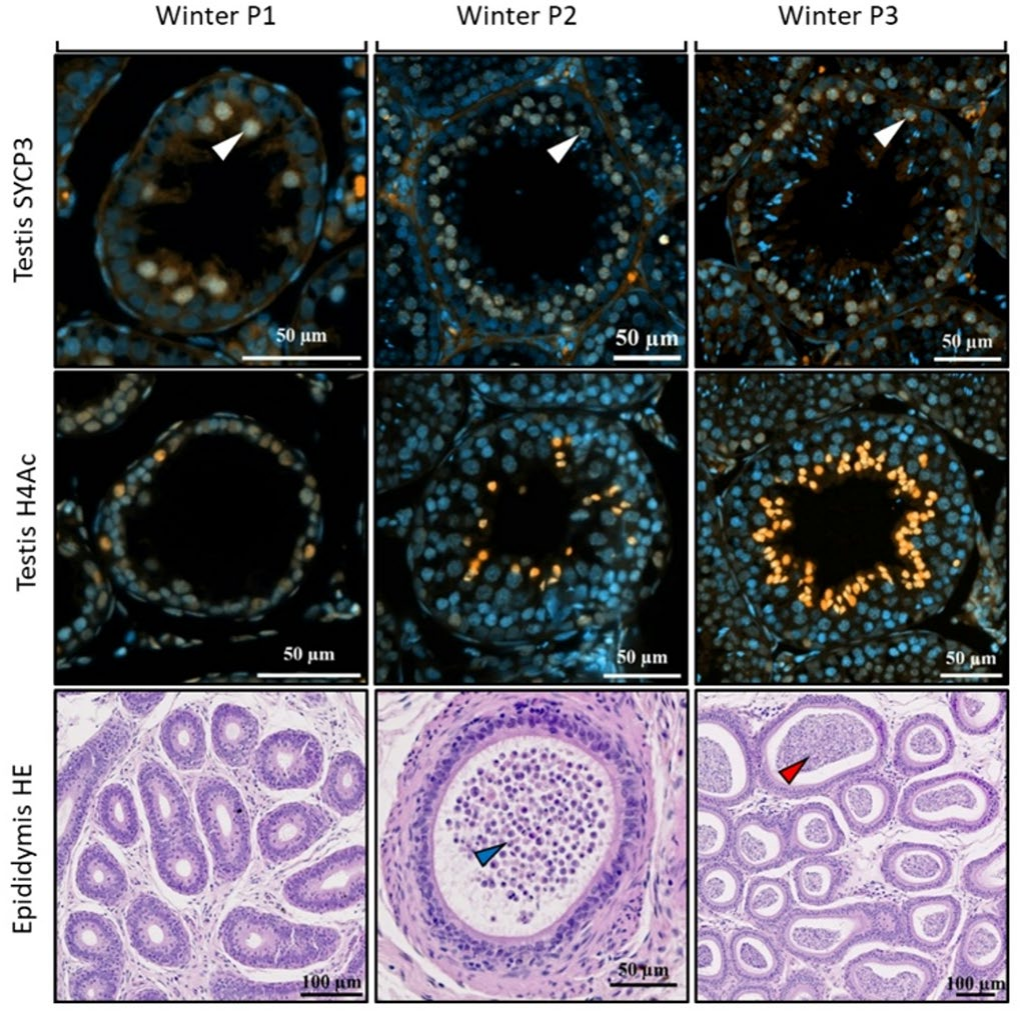
The three different reproductive status of winter males. Early (upper panel, SYCP3) and late (middle panel, H4Ac) stages of spermatogenesis were investigated by immunofluorescence. The histology of the epididymis was characterized by Hematoxylin-eosin staining (lower panel).

Field captures made in summer (July/August) and winter (January/February) for two consecutive years (Fig. 1A, red open arrows) showed differences in terms of animal abundance (Fig. 1B), with no distortion in sex ratio. In winter, we were occasionally able to capture gravid females as well as young animals (< 70 g) attesting to reproductive events (Fig. 1B). A histological evaluation of winter-caught male testes and epididymides was carried-out and Fig. 2 shows representative histochemical sections of winter male seminiferous tubules immuno-detected with the germ cell-restricted meiotic marker SYCP3^27^ (synaptonemal complex protein 3) as well as with the acetylated form of Histone H4 (H4Ac), the classic marker of late spermatogenesis targeting elongated spermatids^28^. This enabled us to distinguish 3 distinct testicular phenotypes within the *ATS* winter male population, illustrated by the presence of animals with empty seminiferous tubules with no sign of spermatogenesis (Phenotype 1: Winter P1), animals with signs of complete spermatogenetic function (Phenotype 3: Winter P3) or in an intermediate situation (Phenotype 2: Winter P2). The absence or presence (red arrow in Fig. 2 right panel) of spermatozoa in the lumen of the epididymal tubules, as illustrated by the hematoxylin/eosin sections, confirms that spermatogenesis was indeed complete in some winter males, whereas it was not in others. The cells indicated by the blue arrow (middle panel) have the morphological characteristics of immature germ cells.

**Fig. 3:**
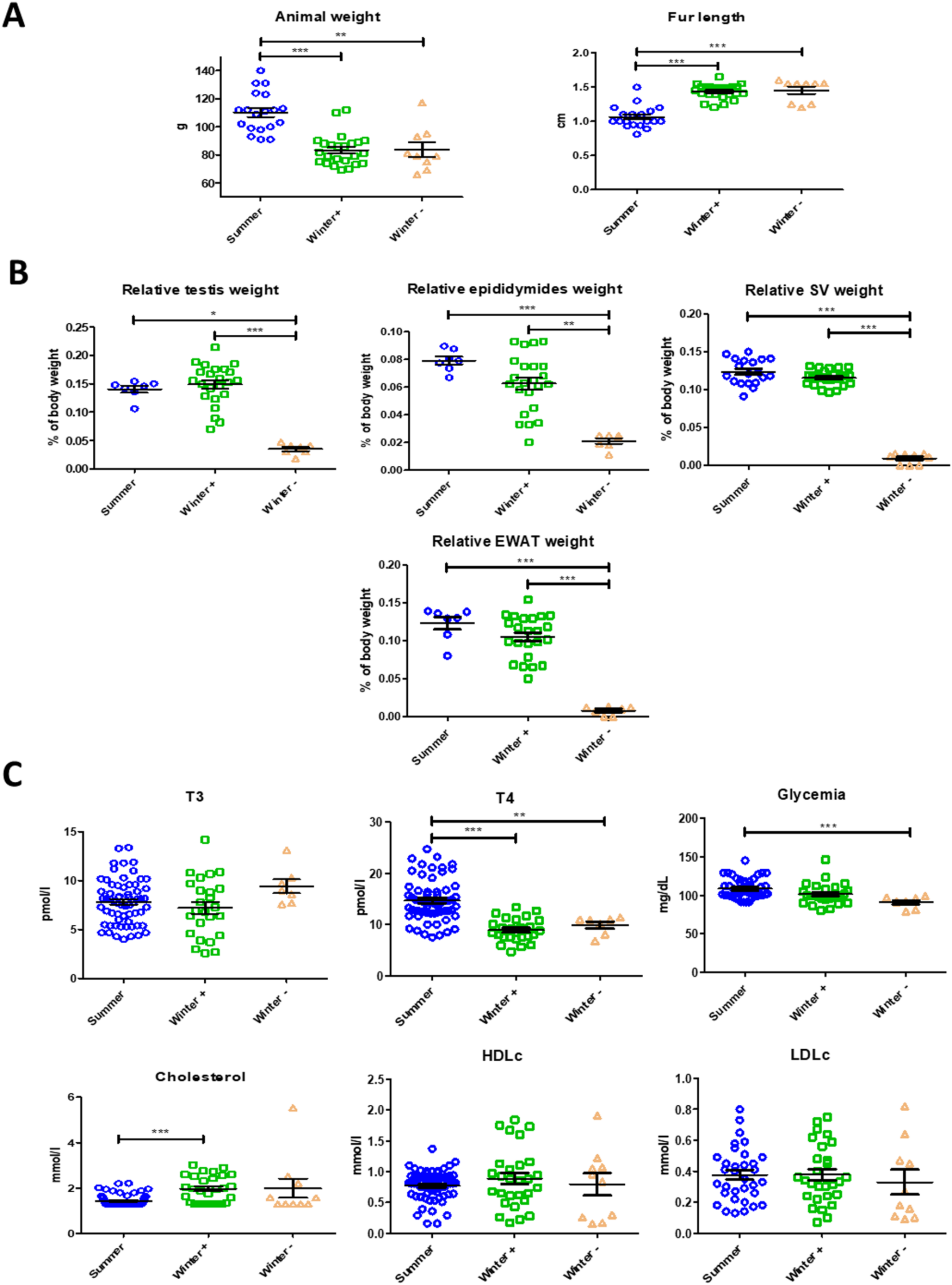
Winter^+^ males keep normal reproductive function. (**A**) W+ males (green squares) showed body weight and fur length similar to W^−^ males (orange triangles), (**B**) whereas their relative testis, epididymis, seminal vesicles and epididymal white adipose tissue weights were similar to summer males (blue circles). (**C**) The circulating thyroid hormones, glycemia and blood cholesterol levels (total, HDL and LDL) of the three groups showed few variations. *P < 0.05, **P < 0.01 and ***P < 0.001 when groups were compared using the Kruskal-Wallis test followed by Dunn’s Multiple Comparison Test. Data are represented as scatter plots and the bar represents the mean ± SEM.

Winter males were then distributed in 2 subgroups of winter animals arbitrarily named “Winter^+^ = W^+^” (corresponding to phenotypes P3) and “Winter ^−^ = W^−^” (corresponding to phenotypes P1 and P2), for males that maintain or do not maintain spermatogenesis during the winter season, respectively. These groups were used for further studies and comparisons with summer males (S). In an initial comparative assessment, we examined gross anatomical features (body weight and fur length) typical of phenotypic differences between summer and winter voles (Fig. 3A). For these two specific parameters, W^+^ and W^−^ animals were indistinguishable from each other and both significantly different from summer voles (S). In a second series of phenotypic assessments, we measured the classic anatomical parameters of the male genitalia between the 3 subgroups of animals (Fig. 3B). The mean relative weight of testes, epididymides and seminal vesicles was found to be significantly reduced in W^−^ animals. W^+^ animals, on the other hand, showed little variation from summer (S) animals. Only the mean relative weight of the epididymides of W^+^ males tend to be slightly lower than that of the S group (not reaching statistical significance). Confirming that W^+^ animals were very similar to S animals, it was observed that their epididymal white adipose tissue (EWAT), known to be involved in supporting spermatogenesis^29^, was almost equivalent to that of S animals (Fig. 3B).

When looking at the systemic compartment (Fig. 3C), glycemia and the thyroid hormone T4 were significantly lower in W^−^ compared to S animals, whereas T4 (decrease) and cholesterol (increase) were significantly different in W^+^ animals compared to S animals. Testosterone could not be measured due to detection sensitivity issues in the majority of the samples.

**Fig. 4:**
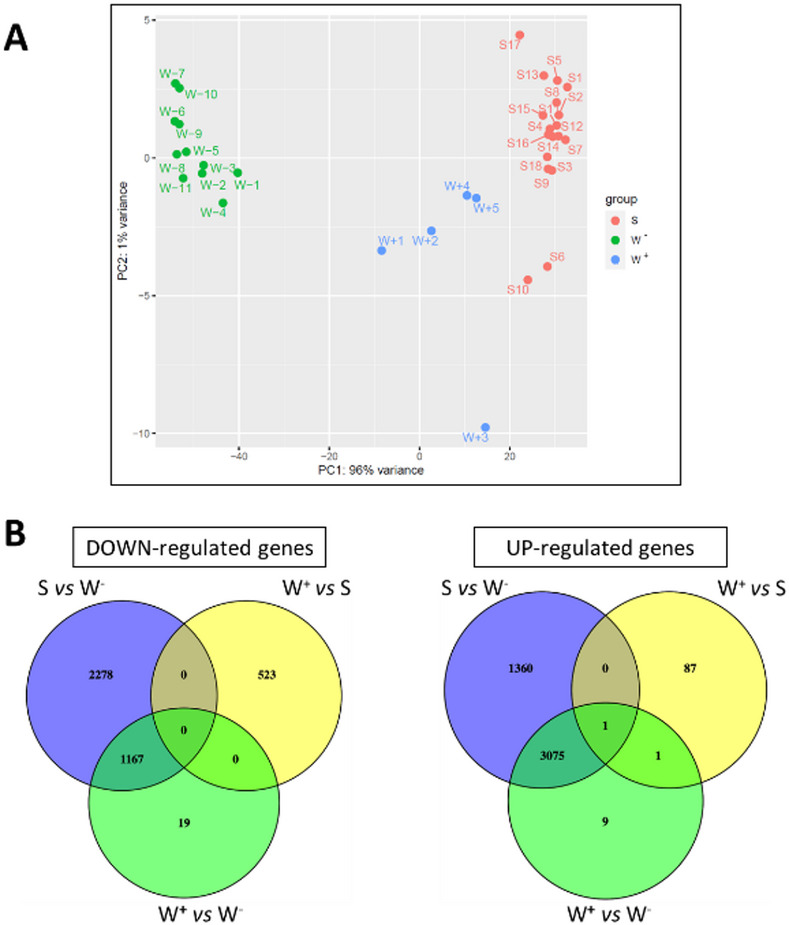
The W^+^ males have a specific testicular transcriptome. (**A**) PCA analysis of the RNAseq data from testis of winter and summer males. The first component explains 96% of the variance. The W^+^ males are distinguished in blue. (**B**) Venn diagrams comparing the number of differentially expressed genes (with log2 fold change > 1; p-value ˂ 0.05) between the three groups of animals. Down-regulated genes are shown in the left panel and up-regulated ones in the right panel.

RNAseq analysis was performed on the testes of a group of 34 animals (16 captured in winter and 18 captured in summer) to assess their gene expression profiles. A principal component analysis presented in Fig. 4A clearly identified 3 groups of animals with distinct expression profiles. The summer animals (*N* = 18) were rather well grouped, while the winter animals (*N* = 16) showed two distinct profiles (Fig. 4A). Interestingly, the 5 winter animals with an expression profile different from that of the W^−^ and S animals (W^+ 1^ to W^+ 5^, Fig. 4A) are those which, according to the anatomical assessment, belong to the W^+^ group meaning they have retained their male reproductive characteristics. The number of differentially expressed genes (DEGs) in Table 1 showed that between summer and winter animals, there are almost 8,000 genes differentially expressed in the testis (3445 down-regulated and 4435 up-regulated). When W^+^ animals were compared to W^−^ animals, over 4,000 testicular genes were differentially expressed between the two groups of animals (1186 down-regulated and 3086 up-regulated). Finally, when W^+^ animals were compared with S animals, only 612 testicular genes were differentially expressed (523 down-regulated genes and 89 up-regulated genes). Furthermore, the DEGs between W^+^ and S groups showed a specific pattern as compared to others, because nearly all of these DEGs were not shared with other comparisons, as seen in the Venn diagram (Fig. 4B).

**Table 1:** Comparisons of up- and down- regulated genes between the different groups of animals.

	Down-regulated genes	Up-regulated genes
S vs. W^−^	3445	4435
W^+^ vs. W^−^	1186	3086
W^+^ vs. S	523	89

**Fig. 5:**
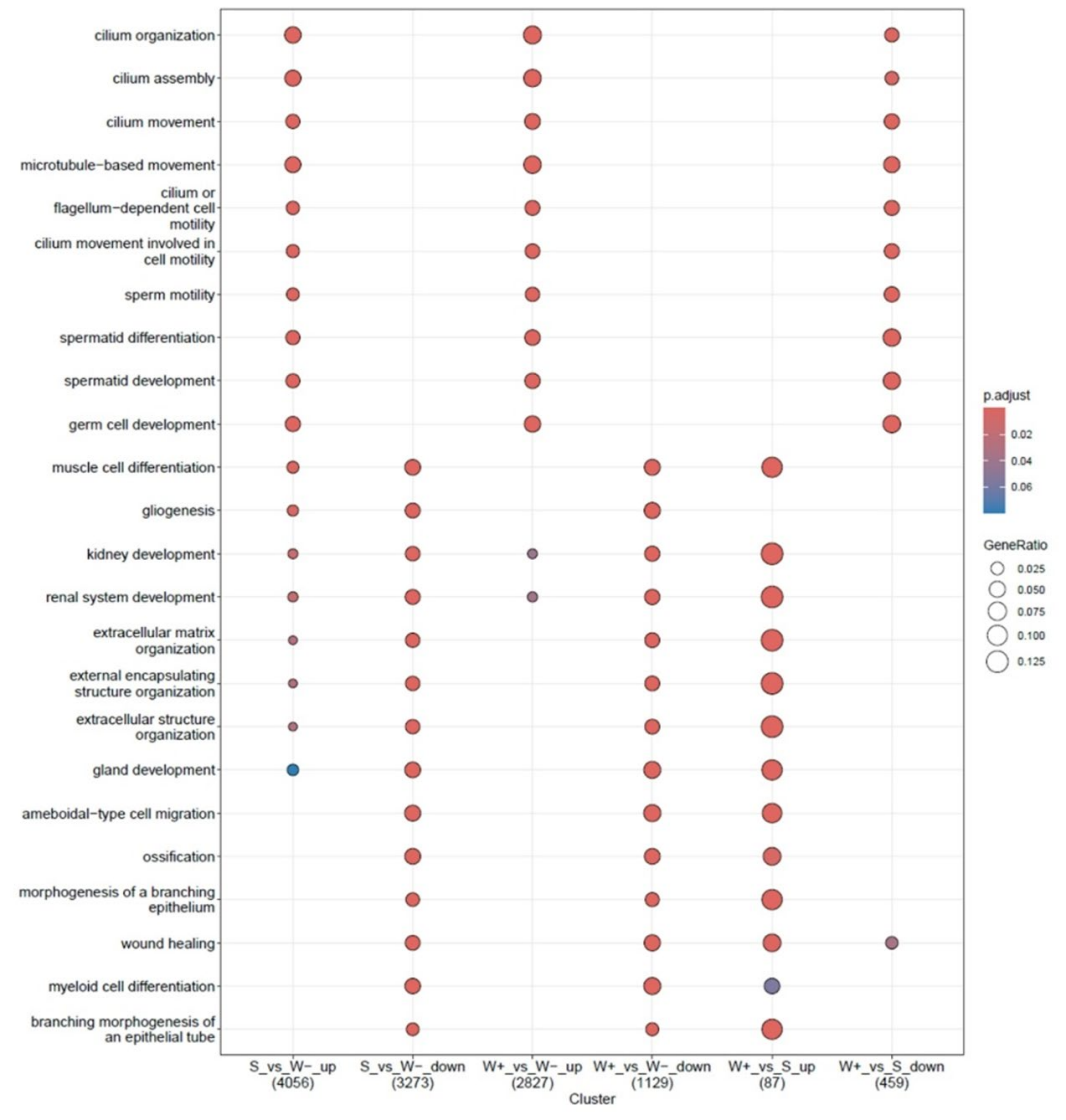
The W^+^ males transcriptome analysis by GO shows their particular testicular activity. Gene ontology analysis comparing the up- and down- regulated genes between the three groups of animals.

Comparing the gene ontology of these DEGs (Fig. 5) between W^+^, W^−^ and S animals resulted in a mirror response, with virtually the same gene pathways and almost identical intensities (Fig. 5, four right panels). The W^+^ animals are thus different from the two other morphs, in accordance with the previous analysis (Fig. 4). The comparison of S animals with W^+^ or W^−^ animals were also identical, showing that S animals were different from the two winter groups. This result shows that W^+^ males have a testicular genetic activity closer to S animals. Consistent with the maintenance of an active spermatogenesis in W^+^ animals, it is interesting to note that gene ontology categories related to cilium, flagellum and germ cell development were up-regulated in W^+^ vs. W^−^ animals but down-regulated in W^+^ vs. S animals, in accordance with the observed phenotypes on testis histology (Fig. 2). This specific signature in testes of W^+^ males, however close to S animals, is confirmed by a PCA analysis of the transcriptomic data two by two (Supplementary Fig. 1) where it clearly appeared that W^+^ animals were really different from W^−^ and close to S.

**Fig. 6:**
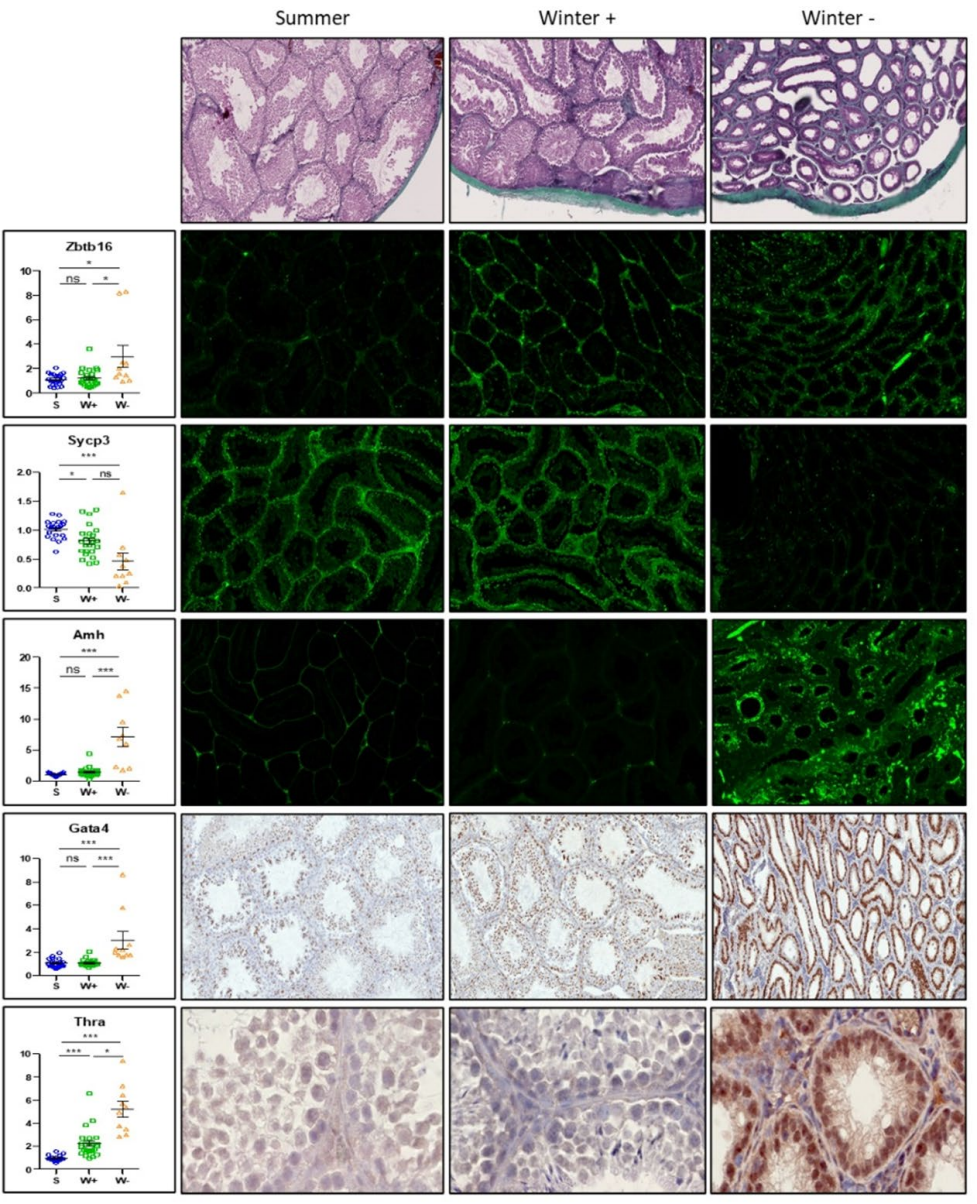
The W^+^ males show testicular gene expression and protein accumulation close to S males. The testis histology appears on the upper panel after Masson’s trichrome staining, and spermatozoa are present in the seminiferous tubules of W+ males. Five targets were selected, to investigate the different steps of spermatogenesis (*Zbtb16* for spermatogonia and *Sycp3* as a meiotic marker), Sertoli cells (*Amh* and *Gata4*) and the thyroid hormone receptor (Thra), respectively from the top to the bottom. Both the qRT-PCR and the immunostaining experiments show that W^+^ males harbor characteristics closer to the S male testis than the W^−^. *P < 0.05 and ***P < 0.001 when groups were compared using the Kruskal-Wallis test followed by Dunn’s Multiple Comparison Test. Data are represented as scatter plots and the bar represents the mean ± SEM.

*Zbtbt16* (also called *Plzf*) is a spermatogonia-specific transcription factor, required to regulate self-renewal and maintenance of the stem cell pool^30^. It was significantly more expressed in W^−^ testes compared to the two other groups (Fig. 6), whereas *Sycp3* (a meiotic marker) showed the inverse pattern of expression (significantly less expressed in W^−^ testes compared to S animals whereas W^+^ animals having an intermediate level between these two groups). These two markers confirm an active spermatogenesis in W^+^ animals, however not at the level of S animals. Two Sertoli cell markers were also investigated: *Amh* (a marker of altered testicular function in adult mammals^31^ and *Gata4* (which regulates Sertoli cell function and the blood-testis barrier^32^. Both showed a higher expression in W^−^ animals compared to the two other groups, in relation with the histology of the organs where only the peripheral basal cells of the tubules remain, i.e., spermatogonia and Sertoli cells. Immunohistological staining of GATA4 showed that the level of protein accumulation was here again intermediate in W^+^ animals. Finally, the alpha isoform of the thyroid hormone receptor (THRA) was also shown to be highly expressed in W^−^ animals, the W^+^ animals having once more an intermediate level of expression. The highest level of protein was obvious in W^−^ animals, whereas it was very faint in W^+^ and S males (immunohistochemistry). The expression levels of the five genes shown in Fig. 6 are in accordance with the RNAseq data (See Supplemental Table 2).

## Discussion

Unlike *Arvicola amphibius*, whose population is gradually declining, leading to its protection as an endangered species, *ATS* is subject to recurrent outbreaks, prompting its classification as a crop and environmental pest. Fossorial water vole populations (*Arvicola scherman*) have been shown to exhibit both cyclic and non-cyclic multi-annual density fluctuations across their range. Such patterns have been documented directly in fossorial water vole populations from different European regions^8,20,33^, thereby providing specific support for statements concerning *ATS* population dynamics. These studies indicate that population fluctuations in this species are spatially heterogeneous and not restricted to the French context. A number of hypothesizes have been proposed in the literature to explain these repeated outbreaks, including the elimination of natural predators (such as foxes, weasels, stoats, …) themselves classified as pests by French environmental agencies. In addition, landscape simplification through the rarefaction of hedges and isolated trees, reducing predators’ habitat, and changes in grassland management leading to the proliferation of dandelions and docks, may provide voles with abundant winter food. While it is entirely reasonable to believe that all these anthropogenic actions have contributed to the steady increase in cyclical *ATS* outbreaks, environmental conditions such as food availability and habitat structure have long been recognized as key determinants of reproduction in wild rodent populations. Winter or extended reproduction in rodents has been interpreted in the context of energy allocation and resource availability, whereby reproduction can occur once primary demands such as thermoregulation and food acquisition are satisfied. In fact, winter or continuous reproduction in *ATS* has already been documented under favorable environmental conditions, including sporadic winter sexual activity at higher altitudes (e.g. Jura populations described by Meylan & Airoldi, 1975^33^) and stable, year-round reproduction at low altitude (e.g. sea-level populations reported by Somoano et al., 2017^20^). More broadly, numerous studies have shown that favorable environments can increase reproductive effort or even extend reproduction throughout the entire year in rodent populations^34–36^.

In the present work we show that in winter, 2 morphs of *ATS* males exist. In the first (named W^−^), and as expected for a seasonal breeder, we find males in which spermatogenesis is completely arrested, as evidenced by several organ, cell and molecular features. Obvious macroscopic characteristics include reduced weight of the testis and of male sexual accessory organs as well as the absence of spermatozoa in the lumen of the seminiferous tubules. At the opposite we found males (named W^+^) that appear to have retained the attributes of animals in which spermatogenesis is fully underway (i.e. no significant difference in relative weight of testis and accessory organs, and presence of spermatozoa in the lumen of seminiferous tubules), close to the phenotype of males in full reproductive period. Interestingly, however, when examining gross phenotypic parameters such as body weight and fur length, W^−^ and W^+^ males are indistinguishable.

Rodents are highly adaptable and they respond to environmental opportunities more quickly and effectively than many other animal species. In tempered regions, rodent reproduction and life expectancy used to be strongly influenced by low winter temperatures and lack of food. The shorter, warmer winters that have characterized the last two decades, at least in the Auvergne volcanic mountain area, may be one of several local environmental factors contributing to winter reproduction, although the scope of the present study does not allow direct evaluation of climatic changes. *ATS* represents a good example of how rodent reproduction can occur outside the expected seasonal window when local conditions are favorable. Normally, a seasonal breeder begins the breeding season in spring and ends it in late autumn. Our survey of *ATS* males in the Sancy Auvergne area suggests that a significant number of animals retain their full reproductive capacity during the winter season. Female *ATS* have also been previously described as seasonal breeders^37^. In the present study, pregnant females and young animals were captured during winter (see Fig. 1), indicating that reproductive activity may persist in some individuals outside the classical breeding season. The biological mechanisms at stake should be investigated in a specific study. The exact contribution of these winter-bred animals to the local *ATS* demography and its expansion the following year is not yet entirely clear. Despite the fact that *ATS* is not a prolific breeder (which is somewhat fortunate! ) winter born animals will be sexually mature earlier in the season, as they reach sexual maturity at the age of two months, and could contribute to population expansion. Even though our observations suggest that breeding seasons, previously described as extending from March to October, may in some areas extend into winter months, they require formal scientific documentation and should be interpreted with caution. The survivors of the winter season, probably young males born late in the *ATS* reproductive period, could contribute to early reproductive activity the following spring. However, it is important to distinguish between winter reproductive activity at the individual level and multi-annual demographic outbreaks at the population level. Although winter-born individuals may participate in the subsequent breeding season, demographic outbreaks are irregular phenomena that result from complex ecological processes acting over longer temporal scales, and cannot be attributed solely to the occurrence of winter reproduction. A detailed study evaluating the ecological factors, such as food availability, that may support winter reproduction would be needed to investigate this point. Accordingly, our interpretations focus on the physiological and molecular mechanisms, rather than on environmental correlates not directly measured in this study.

At the genetic level, and as expected, *ATS* males in full reproductive period (S males) have a transcriptomic signature very distinct from that of the W^-^ males with a substantial number of genes up- and down-regulated (more than 3000 each). Although closer to S males, W^+^ males have also a specific transcriptomic signature (Fig. 4A and supplementary Fig. 1). To validate the RNAseq data, mRNA quantifications were performed by qRT-PCR. Consistent with the fact that spermatogenesis was slowed down in W^-^ males compared to S males, we found a strong decrease in the testis accumulation of *Sycp3* and *Prm2* mRNAs, a meiotic marker and a marker of late spermatogenesis, respectively (Fig. 6 and supplementary Fig. 2). On the contrary, confirming that spermatogenesis was still active in W^+^ males, we observed that the testicular accumulation of these two transcripts was significantly higher than in W^-^ males and quite close to the levels observed in S males, even though *Prm2* was significantly lower in W^+^ animals than in S animals (Fig. 6 and Supplementary Fig. 2). In W^-^ animals, the seminiferous epithelium was thin and appeared to contain only peripheral spermatogonia (Figs. 2 and 6). This was confirmed by the expression levels of the spermatogonia-specific gene *Zbtb16* (and the level of its corresponding protein), which was significantly increased only in W^-^ animals (Fig. 6). Sertoli cell-specific genes, such as *Amh* and *Sox9*, showed the same profile as *Zbtb16*, indicating that these cells were also present and active in the epithelium.

As a seasonal breeder, the male *ATS* must regulate its reproductive function by integrating environmental signals, such as photoperiod, which are transmitted to peripheral organs by several molecular actors. Among these, circulating and peripheral thyroid signaling players and circadian clock genes have already been characterized in rodents and other domestic as well as wild mammals (Refs.^38,39^ and review by^40^). The thyroid gland secretes the prohormone thyroxine (T4), which must be converted to tri-iodothyronine (T3) in target cells by type 2 deiodinase (DIO2), while type 3 deiodinase (DIO3) is the deactivating enzyme. The expression level of *Dio2* was unchanged in W^+^ animals compared to S animals, while it was significantly reduced in W^-^ (supplementary Fig. 2). In contrast, *Dio3* mRNA was upregulated in W^+^ and W^-^ males compared to S animals. These changes promote greater availability of active thyroid hormones (TH) in the testes of W^+^ males, while the degradation pathway is dominant in W^-^ testes. This local regulation of testicular TH appears to be more significant between these two morphs of *ATS* in a context where circulating TH remains comparable (Fig. 3C). In addition, the presence of the TH alpha receptor (THRA, Fig. 6) was higher in the testes of W^-^ animals than in those of the other two morphs. These results are quite surprising when compared to those obtained in other seasonally breeding mammals such as the golden hamster (*Mesocricetus auratus*^41^. In the latter, testicular levels of DIO2 and THRA peak under a long photoperiod (16 h of light corresponding to our S animals) and reach their minimum under a short photoperiod (8 h of light corresponding to our W^-^ animals^-^). In our study, this pattern is different, as levels of *Thra* transcripts (and THRA protein) are not correlated with levels of *Dio2* in W^-^, whereas this is the case in S and W^+^ animals. These results suggest that the level of THRA in the testes of *ATS* may not be regulated solely by testicular TH, particularly during winter. Another explanation could be the state of Sertoli cells in W^-^ animals, as THRA has been described in adult testes to be a reliable indicator of undifferentiated Sertoli cells^38^, which would be consistent with the high levels of *Sox9* and *Amh* expression observed in W^-^ animals.

The peripheral circadian clock is also altered in the testes of male *ATS* from different seasons, and it was clear that W^+^ animals had intermediate expression levels of the various players compared to W^-^ and S animals (Supplementary Fig. 2). The expression of the type 1 A melatonin receptor (*Mtnr1a*) was elevated for S and W^+^ animals and nearly null for W^-^ animals, indicating that photoperiod is likely not the primary environmental signal regulating the peripheral circadian clock in *ATS*. The transcription factor and, one of the main regulators of the circadian clock, *Arntl* (also known as *Bmal1*), showed an expression profile opposite to that of *Mtrn1a*, being significantly higher in W^-^ animals. The BMAL1 protein binds to the CLOCK protein, and the dimer activates the rhythmic transcription of other central clock genes such as *Rorc* (which codes for the ROR gamma protein). This is a negative feedback loop, as RORC rhythmically represses the transcription of *Bmal1*. This antagonism is clear in W^-^ animals, but less obvious in S and W^+^ animals, the latter showing high interindividual variation in *Rorc* expression levels. Strikingly, in W^+^ animals, the level of *Rorc* mRNA is significantly different from that in S and W^-^ animals, confirming the dysregulation of the testicular circadian clock.

In conclusion, this study showed that in the wild population of *ATS*, some males are non-seasonal breeders, which could exacerbate the rhythm of their cyclical epidemics, with a significant economic impact on the farmers who fight them. This finding documents flexibility in the reproductive cycle of this species and highlights that reproductive activity may extend beyond the classical seasonal window. It is important to clearly distinguish this reproductive flexibility from multi-annual density fluctuations or demographic outbreaks, which operate on different temporal scales and are driven by distinct ecological processes. Although we cannot explain the exact cause of the physiological changes in these animals, it has become clear that the main regulators of seasonal testicular activity are not subject to seasonal regulation in these animals. As underground animals, they may have a particular regulation of their circadian mechanisms, different from other rodents, and photoperiod may not be the only environmental factor driving the seasonal adaptation. The occurrence of winter breeders should be interpreted in the context of known flexible reproductive strategies in *ATS* and other rodents under favorable local conditions without necessarily implying recent or unprecedented changes although it cannot be bluntly excluded. The characterization of these winter breeders contributes to a better understanding of the reproductive biology of this species, which represents one component - among others - of its population dynamics. It might help refine management strategies, provided that such applications are grounded in a comprehensive understanding of its population ecology.

## Supplementary Information

Supplementary Information.

## Data Availability

RNA seq data have been deposited on GEO (https://www.ncbi.nlm.nih.gov/geo/) under accession code GSE311418.
