# Supplementary material for: Molecular mechanisms underlying opportunistic seasonal reproduction in the male rodent pest Arvicola terrestris scherman
Source: Sci Rep. 2026 Apr 30;16:20052. doi: 10.1038/s41598-026-51017-9 (PMC13319813; doi:10.1038/s41598-026-51017-9)
Supplement: Supplementary file 1 — Supplementary Information. [file 41598_2026_51017_MOESM1_ESM.pdf]

Supplementary Fig. 1

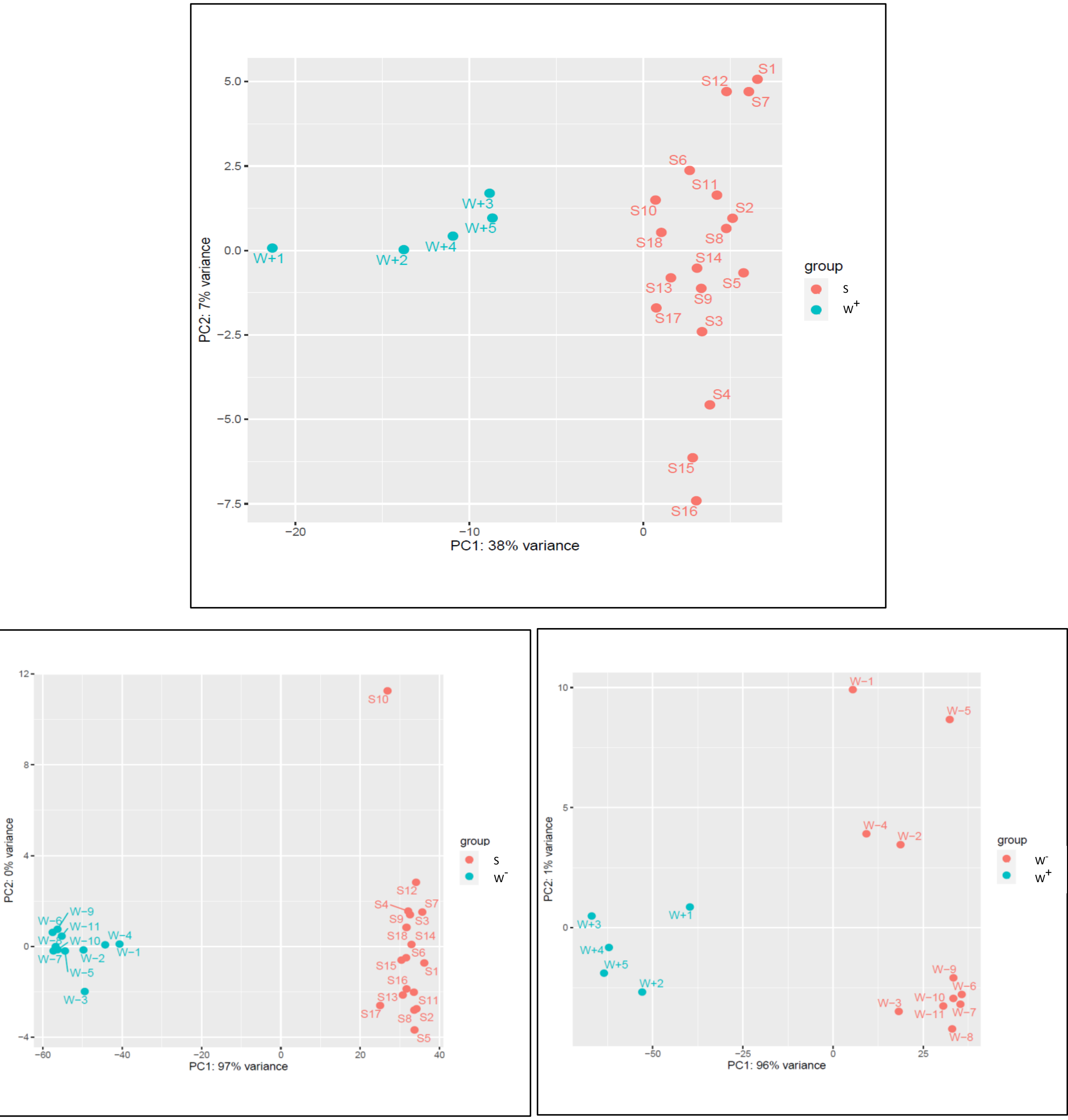

Supplementary Fig. 2

Spermatogenesis

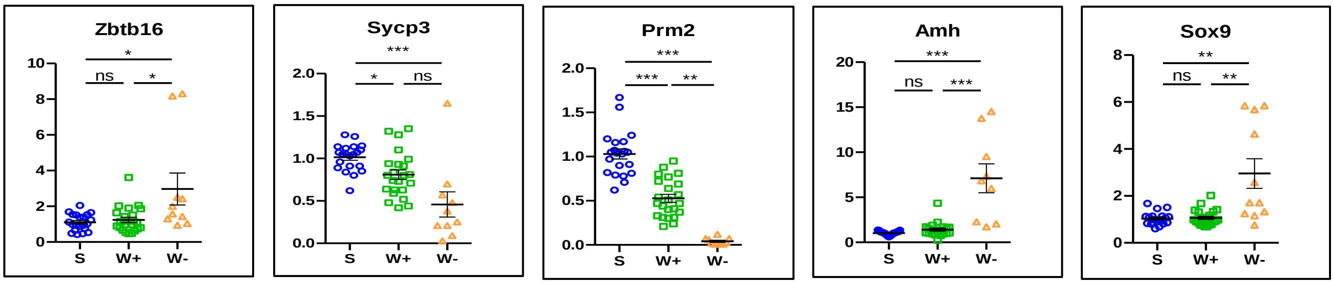

Thyroid hormone pathway

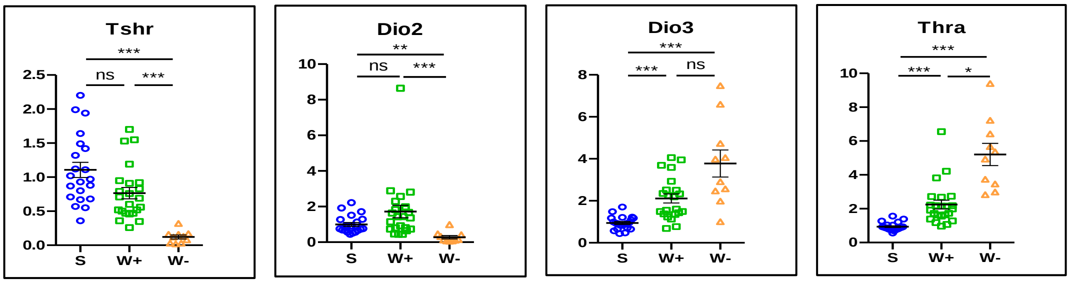

Peripheral circadian clock genes

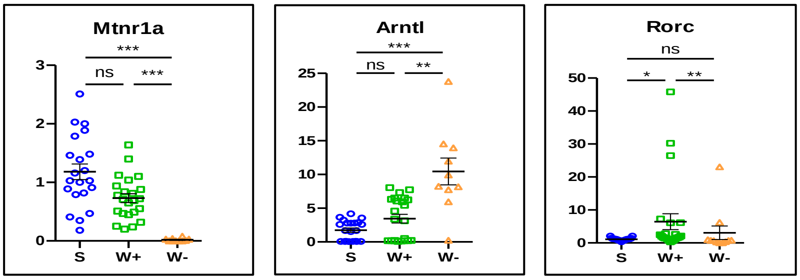

Supplementary Tables

Supplementary Table 1: list of primers.

Gene	Forward	Reverse
**Actb**	CCGCAAATGCTTCTAGGCAG	GCCTTCACCGTTCCAGTTTT
**Amh**	GGCTCTGACTCTCGCTGTTT	AAGCGGGTGAGAGTCTCTAGG
**Arntl**	GAGGCCCACAGTCAGATCG	TCGGATAGAAATGTTGGCTTG
**Dio2**	GGACTCTAGAAGAGACAAGGGT	CCCTCTGAGGTCAACATGCG
**Dio3**	GCCTGGTCACCAAGTACCAA	CCATATGCGGAACTGCTGGA
**Gata4**	CCAAGAACCTGAATAAATCTAAGACA	CCGGACACAGTACTGACAGAGA
**Mtnr1a**	TGGACATCCTGGGCAATTT	CGACACTCAAACCCATCAGA
**Prm2**	CCAGTGAGGGTCAGCACCAG	TCTGCAGCCTCTGCGATGCC
**Rorc**	GCTTGCCTGGCAGAAAAACA	TAGACCTAAGACCCCTCGCC
**Sox9**	CCTTCCTCAAAGGGCATCGT	ACCACAAGGACGGACGAAAA
**Sycp3**	GGAGCTGACATCAACAAAGC	GTATATCCAGTTCCCACTGC
**Thra**	CTGATGTCGACAGACCGCT	AACGTAGTGCTCAAAGGCCA
**Tshr**	TCAAGAAGCTCCCTCTGTCC	TAGAGGGGACCTCTCAGCAC
**Zbtb16**	AACTTCCTCACCTCCATGCAC	ACAGCCAAGAAACCGGAGAT

SUPPLEMENTARY TABLE 2

S vs W- up

0610030E20Rik
1110002E22Rik
1110017D15Rik
1110032A03Rik
1110051M20Rik
1500009C09Rik
1700001K19Rik
1700001L19Rik
1700001O22Rik
1700001P01Rik
1700003F12Rik
1700007K13Rik
1700008O03Rik
1700009N14Rik
1700010I14Rik
1700011L22Rik
1700012B07Rik
1700012B09Rik
1700012P22Rik
1700013F07Rik
1700015G11Rik
1700016C15Rik
1700016D06Rik
1700016H13Rik
1700016K19Rik
1700017N19Rik
1700018B08Rik
1700019A02Rik
1700019D03Rik
1700019N19Rik
1700020A23Rik
1700020L24Rik
1700020N01Rik
1700021F07Rik
1700022I11Rik
1700024G13Rik
1700028J19Rik
1700028P14Rik
1700029H14Rik
1700029I15Rik
1700029J07Rik
1700030J22Rik
1700030K09Rik
1700034E13Rik
1700034J05Rik

1700037C18Rik
1700037H04Rik
1700042G07Rik
1700057G04Rik
1700066M21Rik
1700067K01Rik
1700067P10Rik
1700074P13Rik
1700088E04Rik
1700092M07Rik
1700093K21Rik
1700094D03Rik
1700102P08Rik
1700109H08Rik
1700113H08Rik
1700122O11Rik
1700125H20Rik
1810013L24Rik
1810024B03Rik
2010106E10Rik
2200002J24Rik
2210010C04Rik
2210408I21Rik
2300009A05Rik
2310002L09Rik
2310061I04Rik
2610301B20Rik
2610318N02Rik
2900092C05Rik
3425401B19Rik
4430402I18Rik
4833427G06Rik
4921504E06Rik
4921507P07Rik
4921517D22Rik
4921536K21Rik
4921539E11Rik
4930402F06Rik
4930402H24Rik
4930404N11Rik
4930407I10Rik
4930415O20Rik
4930430F08Rik
4930444P10Rik
4930447F04Rik
4930451I11Rik
4930452B06Rik

4930453N24Rik
4930505A04Rik
4930519G04Rik
4930522H14Rik
4930544D05Rik
4930550C14Rik
4930558K02Rik
4930568D16Rik
4930578I06Rik
4930579F01Rik
4930579G24Rik
4930590J08Rik
4931406B18Rik
4931414P19Rik
4931428F04Rik
4931429L15Rik
4932438A13Rik
4933402J07Rik
4933402N03Rik
4933405L10Rik
4933405O20Rik
4933417A18Rik
4933424G06Rik
4933428M09Rik
4933430I17Rik
5031439G07Rik
5330417C22Rik
5730480H06Rik
5S_rRNA
6330403K07Rik
6820408C15Rik
7SK
9330159F19Rik
9330182L06Rik
9530053A07Rik
9530077C05Rik
9930012K11Rik
9930021J03Rik
a
A1cf
A2m
A2ml1
A430005L14Rik
A730071L15Rik
A930017K11Rik
A930018P22Rik
AA467197

Aaas
Aadacl2
Aadacl2fm3
Aadacl3
Aadacl4
Aadacl4fm4
Aadat
Aak1
Aamdc
Aanat
Aars
Aasdhppt
AB124611
Abca12
Abca14
Abca15
Abca16
Abca17
Abca4
Abca7
Abcb11
Abcc12
Abcf2
Abcf3
Abcg3
Abcg5
Abcg8
Abhd12
Abhd12b
Abhd16b
Abhd2
Abhd5
Abi2
Ablim2
Abra
Abtb1
Acaca
Acadvl
Acap1
Acap2
Acap3
Acbd6
Ace
Ace3
Acer2
Ache
Acot7

Acot8
Acox3
Acoxl
Acp4
Acp7
Acr
Acrbp
Acrv1
Acsbg2
Acsbg3
Acsl1
Acsl5
Acsl6
Acsm1
Acsm3
Acsm4
Acta1
Actbl2
Acte1
Actg2
Actl11
Actl6a
Actl6b
Actl7a
Actl7b
Actl9
Actn3
Actr1a
Actr3b
Actr6
Actrt1
Actrt2
Actrt3
Acvr1c
Acyp1
Ada
Adad1
Adad2
Adal
Adam11
Adam12
Adam18
Adam2
Adam24
Adam28
Adam3
Adam32

Adam5
Adam7
Adamts17
Adamts18
Adamts20
Adamts3
Adamts4
Adamts6
Adamtsl1
Adamtsl3
Adar
Adarb2
Adat1
Adat2
Adcy10
Adcy8
Adgrf4
Adgrf5
Adgrg4
Adgrg7
Adh6a
Adig
Adprm
Adra1d
Adrb1
Adrb3
Adss
Adtrp
Afap1
Aff2
Aff4
Afg1l
Aftph
Agbl1
Agbl2
Agbl4
Agbl5
Ager
Agfg1
Agmo
Ago2
Ago3
Ago4
Agpat1
Agpat2
Agrp
Agtpbp1

Agxt
Ahctf1
Ahcyl2
Ahi1
Ahrr
Ahsg
AI182371
AI661453
Aida
Aif1
Aif1l
Aipl1
Ajm1
Ak1
Ak5
Ak7
Ak8
Ak9
Akain1
Akap1
Akap10
Akap3
Akap4
Akap6
Akirin2
Akna
Aknad1
Akr1e1
Aktip
Aldh1a2
Aldoa
Aldob
Alk
Alkbh3
Allc
Aloxe3
Alpk2
Alpk3
Alx1
Ambn
Amelx
Amer2
Amer3
Ammecr1l
Amn
Amn1
Amotl1

Ampd1
Amz2
Angel1
Angptl8
Ankar
Ankef1
Ankfn1
Ankib1
Ankmy1
Ankrd27
Ankrd29
Ankrd31
Ankrd33
Ankrd34a
Ankrd34c
Ankrd35
Ankrd37
Ankrd40
Ankrd42
Ankrd45
Ankrd49
Ankrd53
Ankrd54
Ankrd55
Ankrd60
Ankrd61
Ankrd9
Anks4b
Anks6
Ankzf1
Anln
Ano10
Ano2
Ano4
Ano5
Ano7
Ano9
Antxrl
Aopep
Ap1m1
Ap1m2
Ap2a1
Ap2b1
Ap3b1
Ap3m2
Ap3s1
Ap4m1

Ap5b1
Ap5m1
Apba1
Apip
Aplnr
Apoa1
Apoa4
Apoa5
Apob
Apobec2
Apoh
Apopt1
Aptx
Aqp11
Aqp12
Aqp7
Aqp9
Arc
Arel1
Arfgap3
Arfgef1
Arfgef2
Arg1
Arg2
Arhgap19
Arhgap20
Arhgap24
Arhgap29
Arhgap33
Arhgap35
Arhgap36
Arhgap40
Arhgap9
Arhgef33
Arid3a
Arid3b
Arih1
Arih2
Arl1
Arl13a
Arl13b
Arl14epl
Arl2bp
Arl3
Arl5b
Arl6
Arl6ip6

Arl9
Armc12
Armc3
Armc4
Armc9
Armh1
Armh4
Armt1
Arntl2
Arrdc5
Arsa
Arsj
Art1
Art3
Asah2
Asap1
Asap3
Asb1
Asb10
Asb13
Asb14
Asb15
Asb16
Asb17
Asb3
Asb4
Asb5
Asb8
Ascc2
Ascl3
Asf1b
Ash2l
Asna1
Asns
Aspa
Aspscr1
Asrgl1
Astn1
Astn2
Asxl2
Asxl3
Atad1
Atad2
Atad2b
Ate1
Atg3
Atg7

Atg9a
Atg9b
Atl3
Atm
Atoh8
Atox1
Atp10a
Atp10b
Atp11b
Atp12a
Atp13a4
Atp13a5
Atp1a3
Atp1a4
Atp1b3
Atp4a
Atp5j
Atp6v1c1
Atp6v1c2
Atp6v1d
Atp6v1e2
Atp6v1fnb
Atp6v1g1
Atp8b3
Atp8b4
Atp8b5
Atp9a
Atp9b
Atpif1
Atr
Atxn10
Atxn2l
Atxn3
Atxn7l1
Atxn7l3b
AU018091
AU022252
Aurka
Avpr1b
AW554918
Awat2
Axdnd1
Azgp1
Azin2
B230118H07Rik
B4galnt2
B4galnt3

B9d1
B9d2
Babam2
Bag1
Bag3
Bag5
Bag6
Baiap3
Banf2
Bap1
Batf2
Baz2b
Bbof1
Bbs1
Bbs10
Bbs2
Bbs5
Bbs7
Bbs9
BC004004
BC031181
BC048562
BC048671
BC049352
Bcan
Bcap29
Bcat1
Bcl11a
Bcl2l12
Bcl2l14
Bcl2l15
Bcl7c
Bclaf3
Bco1
Bco2
Bdh1
Bdkrb1
Bhlha9
Bhmt2
Bin2
Bin3
Birc5
Blk
Blm
Bloc1s6
Blzf1
Bmerb1

Bmp15
Bmp3
Bmp6
Bmp8b
Bnc2
Bnip5
Boll
Borcs5
Bpi
Bpifa1
Bpifa3
Bpifa5
Bpifb1
Bpifb2
Bpifb5
Bpifc
Braf
Brap
Brd2
Brdt
Bri3bp
Brinp1
Brinp2
Brinp3
Brsk1
Bsn
Btaf1
Btbd1
Btbd10
Btbd16
Btbd18
Btbd19
Btbd7
Btbd9
Btc
Btg4
Btla
Btrc
Bub1b
Bud31
C2cd3
C2cd4c
C2cd6
C87436
C8a
C9orf72
Cab39l

Cabcoco1
Cabp2
Cabp4
Cabyr
Cacna1e
Cacna1h
Cacna1i
Cacna1s
Cacna2d3
Cacna2d4
Cacng2
Cacng4
Cacng5
Cacng7
Cacng8
Cacul1
Cadm4
Cadps
Cage1
Calb2
Calcrl
Calhm1
Calhm3
Calm1
Calm3
CALML3
Caln1
Calr3
Caly
Camk4
Camkmt
Camkv
Caml
Camsap1
Camsap2
Cand1
Cant1
Cap2
Capn11
Capn8
Capns1
Caps2
Capsl
Capza3
Capzb
Car1
Car10

Car15
Car6
Carf
Carhsp1
Carm1
Carnmt1
Cars
Casc1
Casc4
Casq1
Casr
Cast
Catip
Catsper1
Catsper2
Catsper3
Catsper4
Catsperd
Catsperz
Cbl
Cblif
Cbll1
Cbln4
Cbx2
Cby1
Cby2
Cby3
Cc2d2b
Ccar1
Ccdc103
Ccdc105
Ccdc106
Ccdc110
Ccdc113
Ccdc114
Ccdc116
Ccdc117
Ccdc124
Ccdc134
Ccdc136
Ccdc14
Ccdc146
Ccdc148
Ccdc15
Ccdc150
Ccdc151
Ccdc154

Ccdc155
Ccdc157
Ccdc158
Ccdc159
Ccdc163
Ccdc169
Ccdc171
Ccdc173
Ccdc175
Ccdc178
Ccdc18
Ccdc180
Ccdc181
Ccdc182
Ccdc184
Ccdc186
Ccdc187
Ccdc189
Ccdc192
ccdc198
Ccdc24
Ccdc27
Ccdc30
Ccdc33
Ccdc34
Ccdc38
Ccdc39
Ccdc42
Ccdc59
Ccdc60
Ccdc62
Ccdc63
Ccdc65
Ccdc68
Ccdc70
Ccdc74a
Ccdc77
Ccdc81
Ccdc82
Ccdc88a
Ccdc88b
Ccdc89
Ccdc91
Ccdc92
Ccdc92b
Ccdc93
Ccdc9b

Ccer1
Cchcr1
Ccin
Ccl1
Ccl2
Ccl24
Ccl26
Ccn4
Ccna1
Ccnb2
Ccnc
Ccnd3
Ccnh
Ccnk
Ccno
Ccnyl1
Ccp110
Ccpg1
Ccpg1os
Ccr6
Ccr8
Cct2
Cct3
Cct4
Cct5
Cct6b
Cd164l2
Cd207
Cd209e
Cd37
Cd3eap
Cd46
Cd84
Cdc123
Cdc14a
Cdc14b
Cdc25c
Cdc42ep3
Cdc45
Cdca2
Cdca3
Cdca5
Cdca8
Cdcp1
Cdcp3
Cdh10
Cdh12

Cdh4
Cdh7
Cdhr2
Cdhr3
Cdhr4
Cdiptos
Cdk14
Cdk15
Cdk18
Cdk20
Cdk5r1
Cdk5rap2
Cdk5rap3
Cdk7
Cdkal1
Cdkl2
Cdkl4
Cdkl5
Cdkn2aip
Cdkn2b
Cdkn2c
Cdkn3
Cdnf
Cdr2
Cdr2l
Cdrt4
Cds1
Cdv3
Cdyl
Ceacam16
Cebpg
Cela2a
Celf3
Celf4
Celf5
Celf6
Celsr2
Celsr3
Cenpc1
Cenpe
Cenpf
Cenph
Cenpj
Cenpq
Cenpt
Cenpu
Cenpv

Cenpx
Cep104
Cep120
Cep126
Cep128
Cep135
Cep152
Cep162
Cep164
Cep19
Cep192
Cep290
Cep295
Cep295nl
Cep350
Cep41
Cep55
Cep57
Cep57l1
Cep63
Cep70
Cep72
Cep76
Cep83
Cep85l
Cep89
Cep95
Cep97
Cers3
Ces2a
Ces2e
Cetn1
Cetn3
Cetn4
Cfap100
Cfap126
Cfap157
Cfap161
Cfap206
Cfap221
Cfap298
Cfap299
Cfap300
Cfap36
Cfap410
Cfap43
Cfap44

Cfap45
Cfap47
Cfap52
Cfap54
Cfap57
Cfap58
Cfap65
Cfap69
Cfap70
Cfap77
Cfap97
Cfap97d1
Cfap97d2
Cfap99
Cfhr1
Cfl2
Cftr
Cgas
Chaf1a
Chaf1b
Chat
Chchd3
Chchd5
Chchd6
Chchd7
Chd5
Chdh
Chfr
Chid1
Chil5
Chmp1b
Chmp6
Chn1
Chn2
Chpt1
Chrm2
Chrm3
Chrm4
Chrm5
Chrna1
Chrna10
Chrna3
Chrnb1
Chrnb2
Chrnb3
Chrne
Chst13

Chst9
Chtf18
Ciapin1
Cib3
Cib4
Cidec
Cinp
Cip2a
Cir1
Cisd1
CK137956
Ckap2l
Cklf
Cks2
Clasp1
Clasrp
Clba1
Clca1
Clca2
Clcn3
Cldn10
Cldn16
Cldn18
Cldn34a
Cldn9
Cldnd2
Clec16a
Clec2m
Clec4a1
Clec4d
Clec4g
Clec4n
Clgn
Clhc1
Clip4
Clmn
Clnk
Clp1
Clpx
Clstn2
Clstn3
Clvs1
Clvs2
Cmah
Cmbl
Cmpk2
Cmss1

Cmya5
CN725425
Cnbd2
Cndp1
Cnga3
Cnga4
Cngb1
Cngb3
Cnih2
Cnnm4
Cnot10
Cntd1
Cntln
Cntn2
Cntn4
Cntnap2
Cntnap4
Cntrl
Cntrob
Cog1
Cog6
Cog8
Coil
Col12a1
Col16a1
Col22a1
Col25a1
Col26a1
Col28a1
Col2a1
Col5a1
Col6a5
Col6a6
Col8a1
Col9a1
Col9a2
Col9a3
Colq
Comp
Cop1
Copg2
Cops5
Coq10b
Coq4
Coq8b
Coq9
Coro2a

Cox5a
Cox6b2
Cpa4
Cpa5
Cpb2
Cpeb2
Cpeb3
Cplane1
Cplane2
Cplx1
Cplx2
Cplx4
Cpm
Cpn1
Cpne4
Cpne9
Cps1
Cptp
Cpvl
Crabp2
Crat
Crb1
Creb1
Creb3l3
Creld2
Crem
Crhbp
Crip3
Cript
Crisp2
Crls1
Crmp1
Crocc2
Crppa
Cryab
Cryba2
Crybb3
Crybg2
Cryzl1
Csde1
Csf3r
Csgalnact1
Csmd1
Csmd2
Csmd3
Csnk1a1
Csnk1d

Csnk1g1
Csnk1g2
Csnk2a2
Csnk2b
Cspg4
Cspp1
Csrnp3
Cst10
Cst13
Cst8
Cstf1
Cstl1
Ctag2
Ctbs
Ctdp1
Ctdspl2
Ctf1
Cthrc1
Ctla4
Ctnna3
Ctnnbl1
Ctsf
Cttnbp2
Ctxn1
Cubn
Cuedc2
Cul3
Cux1
Cuzd1
Cwc15
Cwf19l2
Cwh43
Cxcl17
Cxcl5
Cxcr1
Cyb5d1
Cyb5r1
Cyb5r2
Cyct
Cylc1
Cyp19a1
Cyp1a1
Cyp26c1
Cyp2ab1
Cyp2c55
Cyp2c70
Cyp2g1

Cyp2r1
Cyp3a13
Cyp4f39
Cyp4f40
Cyp51
Cystm1
Cyth2
Cytl1
D130043K22Rik
D16Ertd472e
D1Pas1
D3Ertd751e
D430042O09Rik
D7Ertd443e
Dact3
Dalrd3
Dand5
Dap
Dap3
Dbf4
Dbil5
Dcaf1
Dcaf10
Dcaf12
Dcaf5
Dcaf6
Dcaf7
Dcdc2a
Dchs1
Dclk1
Dclk3
Dcp1b
Dcst1
Dcst2
Dcstamp
Dctn2
Dctn4
Dcun1d3
Dcun1d4
Dcx
Dda1
Ddb2
Ddhd1
Ddit4l
Ddn
Ddx1
Ddx20

Ddx25
Ddx28
Ddx39
Ddx4
Ddx52
Dedd2
Defb13
Defb14
Defb29
Defb50
Degs2
Dennd1b
Dennd5b
Depdc1a
Depdc1b
Dera
Desi1
Desi2
Deup1
Dgat1
Dgat2
Dgka
Dgkb
Dgke
Dgkh
Dgki
Dhrs11
Dhrs7b
Dhrs7c
Dhx16
Dhx30
Dhx34
Dhx40
Dhx58
Diablo
Diaph3
Dido1
Dio1
Dis3l
Dis3l2
Disp3
Dkk1
Dkkl1
Dlat
Dlec1
Dlgap2
Dlgap3

Dlgap5
Dll3
Dlx1
Dlx2
Dlx6
Dmac2l
Dmbx1
Dmp1
Dmrt2
Dmrt3
Dmrta2
Dmrtb1
Dmxl2
Dnaaf1
Dnaaf3
Dnaaf5
Dnah1
Dnah10
Dnah11
Dnah17
Dnah2
Dnah3
Dnah5
Dnah6
Dnah8
Dnaic1
Dnaic2
Dnaja3
Dnajb13
Dnajb14
Dnajb2
Dnajb3
Dnajb4
Dnajb5
Dnajb7
Dnajb8
Dnajb9
Dnajc1
Dnajc15
Dnajc18
Dnajc21
Dnajc24
Dnajc27
Dnajc28
Dnajc3
Dnajc4
DNAJC5B

Dnajc5g
Dnal1
Dnal4
Dnali1
Dnase1l3
Dner
Dnhd1
Dnm1l
Dnm2
Dntt
Doc2a
Dock2
Dok5
Donson
Dop1a
Dot1l
Dpep3
Dpf3
Dpp10
Dpp7
Dpp8
Dpy19l1
Dpy19l2
Dpy30
Dpys
Dpysl5
Dr1
Dram1
Drap1
Drc1
Drc3
Drc7
DRD2
Drd3
Drosha
Dsc1
Dscam
Dscaml1
Dsg2
Dsg3
Dsg4
Dtd1
Dtnb
Dupd1
Dusp10
Dusp13
Dusp15

Dusp18
Dusp21
Dusp27
Dusp4
Dusp6
Dydc1
Dydc2
Dym
Dync1h1
Dync2h1
Dync2li1
Dynll2
Dynlrb2
Dyrk1b
Dyrk3
Dyrk4
Dzip1
E2f5
E2f8
Eaf1
Ebf2
Ebi3
Ecd
Ect2
Ect2l
Eda
Edf1
Edil3
Eef2kmt
Eefsec
Efcab1
Efcab10
Efcab11
Efcab12
Efcab15
Efcab2
Efcab3
Efcab5
Efcab6
Efcab7
Efcab9
Efemp1
Efhb
Efhc1
Efhc2
Efhd1
Efl1

Efna3
Efnb3
Egfem1
Egln2
Egr3
Ehbp1
Ehd1
Eif2b5
Eif2s2
Eif4e
Eif4e3
Eif4g1
Eif4g3
Elavl4
Elf2
Elf5
Elfn2
Ell2
Elmod1
Elmod2
Elovl4
Elp5
Emc2
Emc3
Emc7
Eme1
Emilin2
Eml6
Emsy
Enam
Endou
Enkd1
Enkur
Eno3
Eno4
Enpp3
Enthd1
Entr1
Ep400
Epc1
Epcam
Epgn
Epha3
Epha5
Epha6
Epha7
Epor

Eps8l3
Epsti1
Epx
Eqtn
Ergic2
Eri2
Erich2
Erich3
Erlec1
Erlin2
Ermap
Ermn
Ero1lb
Esm1
Espl1
Esr1
Esrrg
Etfrf1
Etnk1
Etnppl
Etv1
Etv2
Evl
Evx2
Exd1
Exd2
Exoc3
Exoc3l4
Exoc5
Exosc3
Exosc5
Extl2
Eya1
Eya3
Eya4
F11
F2rl2
Fa2h
Faah
Faap100
Fabp6
Fads2b
Faf1
Faf2
Faim2
Faiml
Fam104a

Fam107b
Fam110a
Fam118a
Fam122c
Fam126a
Fam126b
Fam129c
Fam131b
Fam149b
Fam151b
Fam161a
Fam166a
Fam166b
Fam166c
Fam167b
Fam170a
Fam170b
Fam174b
Fam178b
Fam183b
Fam186a
Fam186b
Fam187a
Fam187b
Fam193a
Fam205c
Fam209
Fam214b
Fam216a
Fam217a
Fam219a
Fam221a
Fam221b
Fam222a
Fam227a
Fam227b
Fam228a
Fam228b
Fam229a
Fam229b
Fam243
Fam50b
Fam57b
Fam71a
Fam71d
Fam71e1
Fam71f1

Fam71f2
Fam76a
Fam76b
Fam78a
Fam81a
Fam81b
Fam83c
Fam83e
Fam91a1
Fam92a
Fam98a
Fancd2os
Fancg
Fank1
Far2
Fars2
Fat2
Fbn2
Fbp1
Fbxl13
Fbxl15
Fbxl2
Fbxo15
Fbxo24
Fbxo27
Fbxo3
Fbxo34
Fbxo36
Fbxo39
Fbxo4
Fbxo40
Fbxo43
Fbxw10
Fbxw5
Fbxw7
Fcamr
Fcgbp
Fcgr1
Fdxacb1
Fem1b
Fem1c
Fen1
Fer
Fer1l5
Fer1l6
Ferd3l
Fermt1

Fev
Fez1
Fga
Fgd3
Fgf10
Fgf12
Fgf14
Fgf2
Fgf21
Fgf9
Fgfbp1
Fgfr1op2
Fgg
Fhad1
Fhdc1
Fhit
Fhl5
Filip1l
Fis1
Fkbp1b
Fkbp4
Fkbp6
Fkbp7
Fkbpl
Flacc1
Flcn
Flrt2
Flywch2
Fmc1
Fmn1
Fmo6
Fndc1
Fndc11
Fndc3a
Fndc3b
Fndc8
Fndc9
Fnip1
Fnta
Fosl1
Foxd2
Foxg1
Foxh1
Foxj1
Foxk1
Foxm1
Foxp2

Frat2
Frem1
Frem2
Frs3
Fscn2
Fscn3
Fsd1l
Fsd2
Fsip1
Fsip2
Fsip2l
Fstl5
Ftmt
Fut11
Fxr1
Fxyd2
Fyco1
Fyttd1
Fzr1
G6pc3
Gabarapl1
Gabbr1
Gabbr2
Gabra4
Gabrb1
Gabrb2
Gabrg2
Gabrp
Gabrr1
Gabrr2
Gabrr3
Gad2
Gadl1
Galc
Galk2
Galm
Galnt14
Galnt3
Galntl5
Galp
Gan
Gapdhs
Garem2
Gas2
Gas2l3
Gas8
Gask1b

Gata2
Gata3
Gatd3a
Gbf1
Gcc1
Gcfc2
Gck
Gcm2
Gcnt1
Gcnt3
Gcsam
Gdap1l1
Gde1
Gdf11
Gdf2
Gdf6
Gdf7
Gdpd4
Gemin5
Gemin6
Gemin7
Gen1
Gfap
Gfer
Gfod2
Gfpt1
Gfy
Gga1
Gga3
Ggct
Ggh
Ggn
Ggnbp1
Ggnbp2
Ggt5
Ghitm
Ghsr
Gid4
Gins2
Gins3
Gip
Gipr
Git1
Gja5
Gk2
Gkap1
Glb1l

Glb1l2
Glcci1
Gldn
Glipr1l2
Glp1r
Glra3
Gls2
Glt1d1
Glt6d1
Glyat
Glyatl3
Glyctk
Gm11437
Gm11634
Gm11992
Gm12695
Gm128
Gm13420
Gm136
Gm17018
Gm17359
Gm17949
Gm20403
Gm2115
Gm21976
Gm22299
Gm27301
Gm27395
Gm27454
Gm27539
Gm27543
Gm27608
Gm27717
Gm27747
Gm27834
Gm27884
Gm27972
Gm27998
Gm27999
Gm28729
Gm2a
Gm3045
Gm32742
Gm35339
Gm36368
Gm4131
Gm42906

Gm45521
Gm4869
Gm4952
Gm50318
Gm5134
Gm5142
Gm527
Gm5460
Gm572
Gm5737
Gm5767
Gm614
Gm6569
Gm6583
Gm6657
Gm7271
Gm9195
Gm960
Gm973
Gm9999
Gmcl1
Gmfg
Gmnn
Gmppb
Gmpr2
Gmps
Gnao1
Gnat2
Gnat3
Gnb3
Gng3
Gngt2
Gnl1
Gnpat
Gnpda2
Gnpnat1
Golga4
Golga7b
Golgb1
Gorasp2
Gosr2
Got1l1
Gp1bb
Gp5
Gpank1
Gpat4
Gpatch11

Gpatch2
Gpatch2l
Gpd2
Gphn
Gpi1
Gpr12
Gpr135
Gpr137
Gpr141
Gpr15
Gpr153
Gpr158
Gpr162
Gpr18
Gpr19
Gpr3
Gpr45
Gpr50
Gpr61
Gpr65
Gps1
Gpx6
Gramd1b
Gramd1c
Gramd2
Grap2
Greb1l
Grhl1
Gria1
Gria2
Grid2ip
Grik1
Grik2
Grin2b
Grin2d
Grin3a
Grip1
Grk3
Grk4
Grm1
Grm3
Grm7
Grm8
Grn
Grxcr2
Gsdma
Gsg1

Gsk3b
Gstcd
Gstm5
Gsto2
Gstt2
Gstt4
Gtf2a1l
Gtf2f1
Gtf2f2
Gtf3c4
Gtpbp2
Gtsf1
Gtsf1l
Gtsf2
Guca1a
Gucy1a2
Gucy1b1
Gucy2f
Gulo
Gxylt1
Gys2
H1f6
H2-M5
H2ac12
H2ac21
H2aw
H2ax
H2bc1
H2bc14
H2bu2
Habp2
Habp4
Hadha
Hagh
Haghl
Hao1
Hapln1
Hapln2
Hat1
Haus1
Haus4
Hc
Hcfc2
Hcn1
Hdac1
Hddc3
Hdx

Heatr4
Heatr9
Hectd2
Hectd4
Hecw1
Hecw2
Hemgn
Henmt1
Hephl1
Herc1
Herc6
Herpud2
Hes3
Hexa
Hexdc
Hexim2
Hey1
Hgd
Hgs
Hhipl1
Hif1an
Hipk1
Hipk3
Hipk4
Hivep3
Hk1
Hk3
Hmces
Hmcn1
Hmga2
Hmgcll1
Hmgcr
Hnf1b
Hnf4g
Hnrnpul1
Homez
Hook1
Hook2
Hormad1
Hormad2
Hoxb1
Hoxb5
Hoxc8
Hoxc9
Hpca
Hpcal4
Hps4

Hpse2
Hrh4
Hrob
Hs3st2
Hs3st5
Hsbp1l1
Hsdl2
Hsf2bp
Hsf5
Hsfy2
Hspa13
Hspa1l
Hspa4l
Hspb3
Hspb6
Hspb9
Hspbap1
Htr2b
Htr2c
Htr3a
Htra4
Htt
Hus1
Hus1b
Hvcn1
Hyal1
Hyal3
Hyal4
Hyal5
Hyal6
Hydin
Hykk
Hyls1
Hyou1
Ica1l
Icmt
Id4
Ide
Ifih1
Ift122
Ift172
Ift20
Ift22
Ift27
Ift57
Ift74
Ift80

Ift88
Iglon5
Igsf9
Il11
Il12rb2
Il13ra2
Il18
Il18r1
Il18rap
Il1f6
Il1rapl1
Il1rl1
Il20ra
Il2rb
Il34
Il4i1
Il9r
Ilkap
Ilrun
Immp1l
Immp2l
Immt
Impg1
Impg2
Inka1
Ino80c
Ino80e
Inpp1
Inpp4a
Inpp4b
Inpp5e
Inpp5k
Insc
Insl6
Insyn2b
Ints6
Ints7
Ip6k1
Ipcef1
Ipo13
Ipo4
Iqank1
Iqca
Iqca1l
Iqcc
Iqcd
Iqcf1

Iqcf4
Iqcf5
Iqcf6
Iqcg
Iqch
Iqck
Iqcm
Iqcn
Iqgap1
Iqgap2
Iqsec1
Iqub
Irak1bp1
Irf5
Irgc1
Irs4
Isca2
Isg20l2
Isl1
Islr2
Isoc2a
Itga10
Itga9
Itgad
Itgax
Itgb2
Itgb3bp
Itgb6
Itih1
Itih2
Itih5
Itpr2
Itprid1
Itprip
Itpripl1
Izumo1
Izumo2
Izumo3
Izumo4
Jakmip2
Jakmip3
Jazf1
Jchain
Jhy
Jmjd8
Josd1
Jph3

Jrk
Jrkl
Kansl1l
Kat7
Katnal2
Katnb1
Kbtbd3
Kbtbd7
Kcmf1
Kcna4
Kcnab1
Kcnab2
Kcnb2
Kcnc1
Kcnc2
Kcnf1
Kcnh1
Kcnh5
Kcnh6
Kcnh7
Kcnh8
Kcnip2
Kcnip4
Kcnj10
Kcnj13
Kcnj14
Kcnj16
Kcnj2
Kcnj4
Kcnj6
Kcnj9
Kcnk10
Kcnk2
Kcnma1
Kcnmb2
Kcnn2
Kcnn3
Kcnq2
Kcnu1
Kctd11
Kctd16
Kctd19
Kctd7
Kdm3a
Kdm4a
Kdm4d
Kdm5b

Keap1
Khdrbs3
Kif17
Kif18a
Kif18b
Kif19a
Kif21b
Kif24
Kif26b
Kif27
Kif2a
Kif2b
Kif2c
Kif3a
Kif3b
Kif5c
Kif6
Kif9
Kifap3
Kifc3
Kin
Kiz
Klb
Klc3
Klf17
Klf4
Klf5
Klhdc1
Klhdc10
Klhdc3
Klhdc4
Klhdc9
Klhl1
Klhl12
Klhl18
Klhl25
Klhl28
Klhl3
Klhl32
Klhl41
Klhl6
Klhl7
Klk5
Klk6
Klk8
Klkb1
Knop1

Knstrn
Kpna4
Krba1
Krcc1
Kremen2
Krt1
Krt10
Krt12
Krt2
Krt71
Krt73
Krt77
Krt86
Krt87
Krt88
Ksr2
Ktn1
Ky
L1cam
L2hgdh
Lacc1
Lactbl1
Lama3
Lamp5
Lancl1
Lancl2
Laptm4a
Larp4b
Lax1
Lbh
Lca5l
Lck
Lcorl
Lef1
Lefty2
Lekr1
Lemd1
Lep
Lepr
Letm2
Lexm
Lgals12
Lgals7
Lgals8
Lgi1
Lgr5
Lhfpl1

Lhx1
Lhx2
Lhx4
Lhx5
Lhx8
Lias
Lig3
Lim2
Limd2
Limk2
Lin37
Lin52
Lin54
Lin7a
Lin7b
Lin7c
Lin9
Lingo2
Lipe
Lipf
Liph
Lipi
Lkaaear1
Llcfc1
Lmbr1l
Lmntd1
Lmntd2
Lmo7
Lmod3
Lmtk3
Lnpep
Lnpk
Lonp2
Lox
Loxhd1
Lpcat2b
Lpcat3
Lpgat1
Lpin1
Lpin3
Lpp
Lpxn
Lrat
Lrguk
Lrif1
Lrit1
Lrit2

Lrmda
Lrmp
Lrp12
Lrp1b
Lrp2bp
Lrrc1
Lrrc10b
Lrrc18
Lrrc23
Lrrc26
Lrrc27
Lrrc28
Lrrc29
Lrrc3
Lrrc30
Lrrc34
Lrrc36
Lrrc3b
Lrrc3c
Lrrc43
Lrrc46
Lrrc49
Lrrc51
Lrrc52
Lrrc55
Lrrc56
Lrrc57
Lrrc6
Lrrc61
Lrrc63
Lrrc66
Lrrc69
Lrrc7
Lrrc71
Lrrc72
Lrrc73
Lrrc74a
Lrrc8b
Lrrc9
Lrrcc1
Lrrd1
Lrrfip2
Lrriq4
Lrrk1
Lrrtm3
Lrtm2
Lrwd1

Lsm10
Lsm5
Ltf
Lurap1
Luzp1
Ly6g6c
Ly6g6d
Ly6g6e
Ly75
Lyg2
Lyl1
Lypd6b
Lypla1
Lyrm1
Lyzl1
Lyzl4
Lyzl6
Lztfl1
Lzts1
Maats1
Mab21l1
Mab21l4
Maco1
Macrod1
Macrod2
Mad2l1bp
Mad2l2
Maea
Mael
Magel2
Magi2
Majin
Mak
Malrd1
Mamstr
Manf
Map1lc3b
Map2k2
Map3k10
Map3k13
Map3k19
Map6d1
Map7
Map7d3
Mapk10
Mapk15
Mapk6

Mapk8ip1
Mapk8ip2
Mapkap1
Mapre3
Marchf1
Marchf11
Marchf6
Mas1
Masp1
Mbl1
Mbtd1
Mc5r
Mcat
Mcc
Mcidas
Mcm10
Mcm9
Mcmdc2
Mcoln2
Mcoln3
Mcrip1
Mctp1
Mctp2
Mdc1
Mdga1
Mdga2
Mdh1b
Mdm1
Mdm2
Me3
Meaf6
Meak7
Mecr
Med1
Med19
Med26
Med27
Med7
Med9
Mefv
Megf6
Meig1
Meikin
Meiob
Meioc
Meis3
Memo1

Mepe
Mesp1
Mettl22
Mettl6
Mex3b
Mex3c
Mex3d
Mfap3l
Mff
Mfn1
Mfsd11
Mfsd13b
Mfsd14a
Mfsd2b
Mfsd6l
Mgam
Mgat3
Mgat4c
Mgat4d
Mgat4e
Mgat4f
Mgll
Mib1
Micos13
Micu3
Mid1
Mief1
Mif4gd
Miip
Mindy1
Mindy3
Mindy4
Mios
Mir124a-3
Mir125a
Mir135a-1
Mir141
Mir181c
Mir1956
Mir200c
Mir23b
Mir3544
Mir429
Mir448
Mir665
Mir879
Mirlet7e

Mkrn1
Mkrn2
Mlc1
Mlf1
Mlkl
Mllt10
Mllt11
Mlph
Mmadhc
Mme
Mmel1
Mmp12
Mnd1
Mns1
Mob3b
Mog
Mogat1
Mok
Mon1a
Morc1
Morc2a
Morc2b
Morn1
Morn2
Morn3
Morn4
Morn5
Mphosph9
Mpig6b
Mpp4
Mpp6
Mppe1
Mpv17l
Mreg
Mrgbp
Mrm3
Mrnip
Mroh1
Mroh2a
Mroh2b
Mroh3
Mroh4
Mroh5
Mroh7
Mroh8
Mroh9
Mrpl32

Mrpl35
Mrpl58
Mrps22
Mrps24
Mrs2
Mrtfb
Ms4a1
Ms4a12
Ms4a13
Ms4a14
Ms4a5
Msantd1
Msh4
Msi2
Msl1
Msra
Msrb3
Mstn
Msto1
Mtbp
Mtch2
Mtf1
Mtfr1
Mtfr2
Mthfsd
Mtif3
Mtmr12
Mtmr6
Mtmr9
Mtnr1a
Mtnr1b
Mtor
Muc20
Mucl3
Mul1
Musk
Mxd1
Mxd3
Mybl1
Mybpc1
Mybpc2
Mycbp
Mycbpap
Myh1
Myh10
Myh13
Myh15

Myh2
Myh3
Myl1
Myl2
Myl4
Myl7
Mylk4
Mymx
Myo16
Myo18b
Myo19
Myo1a
Myo1c
Myo1h
Myo3b
Myo5b
Myo9a
Myoc
Myog
Myom3
Myorg
Mypn
Myrfl
Myt1
Myt1l
Mzb1
N4bp2l2
Naa11
Naa38
Naa60
Naa80
Nabp1
Nabp2
Naif1
Napa
Napepld
Naprt
Nasp
Nat9
Nav3
Nbas
Nbea
Ncan
Ncapg
Ncaph
NCKIPSD
Ncoa4

Ndc80
Ndfip2
Ndst4
Ndufa12
Ndufaf2
Ndufaf7
Ndufs1
Ndufs4
Ndufs6
Neb
Nebl
Necab1
Necab3
Nectin3
Nedd4l
Negr1
Nek10
Nek11
Nek2
Nek5
Nelfe
Nell2
Nemf
Nemp1
Nepn
Neurl1a
Neurod2
Neurod6
Nexmif
Nf1
Nfasc
Nfatc2
Nfatc2ip
Nfe2
Nfkbib
Nfkbie
Nfkbil1
Nfrkb
Nfs1
Nfu1
Ngb
Ngef
Ngly1
Nhlh2
Nhlrc4
Nifk
Nim1k

Nipsnap3a
Nit1
Nkapl
Nkiras1
Nkiras2
Nkx1-2
Nkx2-2
Nkx2-3
Nkx2-6
Nkx6-1
Nlgn1
Nlrc4
Nlrc5
Nlrp14
Nme5
Nme7
Nme8
Nmnat1
Nmnat2
Nmt1
Nmu
Nmur1
Nmur2
Nnmt
Nol4
Nol7
Nom1
Nos1ap
Nos2
Nostrin
Noxo1
Noxred1
Npas4
Npbwr1
Npc1l1
Npepl1
Nphp1
Nphs2
Npm2
Npm3
Npy2r
Nr1h3
Nr1h5
Nr1i2
Nr2e1
Nr4a2
Nr6a1

Nrap
Nrd1
Nrg4
Nrip2
Nrm
Nrsn1
Nrxn2
Nrxn3
Nsd2
Nsmce1
Nsmce2
Nsmf
Nsun4
Nsun7
Nt5c1a
Nt5c1b
Ntf3
Ntm
Ntmt1
Ntrk3
Nuak1
Nudcd3
Nudt16l2
Nudt18
Nudt21
Nudt22
Nudt4
Nudt6
Nudt7
Nudt9
Nufip2
Numbl
Nup153
Nup155
Nup210l
Nup214
Nup37
Nup50
Nup54
Nup88
Nup98
Nupl2
Nupr1l
Nutm1
Nwd1
Nwd2
Nxnl1

Nxt1
Nxt2
Nyap2
Oaz2
Oaz3
Oc90
Oca2
Ocstamp
Odaph
Odf1
Odf2
Odf2l
Odf3
Odf3b
Odf3l1
Odf3l2
Ofcc1
Oit1
Olfm2
Olfm4
Olfr1006
Olfr1038-ps
Olfr124
Olfr1336
Olfr1347
Olfr1348
Olfr1361
Olfr1370
Olfr1440
Olfr187
Olfr273
Olfr332
Olfr411
Olfr430
Olfr478
Olfr5
Olfr514
Olfr520
Olfr521
Olfr572
Olfr718-ps1
Olfr78
Olfr90
Olfr93
Olfr95
Olig3
Omg

Onecut1
Opcml
Oplah
Opn1sw
Opn3
Oprl1
Optn
Orai2
Orc3
Osbp
Osbp2
Osbpl10
Osbpl2
Osbpl5
Osbpl8
Osbpl9
Oscp1
Oser1
Osgin1
Otoa
Otof
Otog
Otol1
Otop1
Otos
Otub2
Otud4
Otud7b
Ovch2
Ovol1
Ovol3
Oxgr1
Oxsm
OXTR
P2rx3
P2rx7
P4ha3
Pabpc2
Pacrg
Pacs1
Pacsin3
Padi1
Padi3
Pafah1b1
Pak7
Palm3
Palmd

Pan3
Pank2
Panx3
Paox
Pappa
Pappa2
Papss2
Paqr3
Paqr5
Paqr9
Pard6a
Parp6
Parpbp
Pask
Pate13
Patl2
Pax2
Pax3
Pax4
Pax5
Pax6
Pax9
Pbk
Pbx4
Pcare
Pcbd2
Pcbp3
Pcdh10
Pcdh15
Pcdha1
Pcdha3
Pcdha6
Pcdhac2
Pcdhb1
Pcdhb13
Pcdhb14
Pcdhb18
Pcdhga11
Pcdhga5
Pcdhgb4
Pcdhgb6
Pcdhgb7
Pcf11
Pcgf6
Pck1
Pclaf
Pclo

Pcm1
Pcmt1
Pcnx
Pcsk1
Pcsk2
Pcsk4
Pdcd1lg2
Pdcd2l
Pdcd4
Pdcd5
Pdcl2
Pde11a
Pde1a
Pde1c
Pde3b
Pde4a
Pde4c
Pde4dip
Pde6b
Pde8a
Pdhb
Pdilt
Pdk2
Pdp1
Pdpk1
Pdzd8
Pdzd9
Pdzk1
Pdzk1ip1
Pdzrn4
Peak1
Peli3
Pemt
Pex11a
Pex11b
Pex11g
Pex12
Pex13
Pex3
Pfkp
Pfn3
Pfn4
Pgam2
Pgk2
Pglyrp3
Pgr15l
Pgrmc2

Phactr3
Pheta2
Phf1
Phf10
Phf14
Phf19
Phf2
Phf21a
Phf7
Phkg2
Phlda2
Phlpp1
Phospho1
Phospho2
Phpt1
Phtf1
Phyhipl
Pias2
Pias4
Picalm
Pick1
Pif1
Pifo
Pigc
Pigf
Pigm
Pigr
Pigx
Pigyl
Pih1d2
Pik3ap1
Pik3cg
Pim1
Pinlyp
Pip5kl1
Pirt
Pithd1
Pitpna
Piwil1
Pja2
Pjvk
Pkd1l2
Pkd2l1
Pkd2l2
Pkib
Pkig
Pkmyt1

Pkn2
Pknox1
Pknox2
Pkp1
Pla2g10
Pla2g12b
Pla2g2c
Pla2g4d
Pla2g6
Plaat1
Plaat5
Plac1
Plac8
Plac8l1
Plb1
Plbd1
Plcb1
Plcb2
Plcd1
Plcd4
Plce1
Plch1
Plch2
Plcxd2
Plcz1
Pld5
Plec
Plek
Plekha2
Plekha3
Plekha5
Plekhh2
Plekhm2
Plekho2
Plk1
Plk4
Pllp
Pln
Plpp1
Plpp6
Pmch
Pmel
Pmfbp1
Pmis2
Pml
Pnldc1
Pnlip

Pnliprp1
Pnliprp2
Pnpla2
Poc1a
Poc1b
Podnl1
Pola2
Polb
Pold3
Pole4
Polg
Polh
Poli
Polk
Poln
Polr2i
Polr3gl
Pom121
Pom121l12
Pomc
Pomt1
Pomt2
Popdc2
Popdc3
Pou2f1
Pou2f3
Pou4f3
Pou5f2
Pp2d1
Pparg
Ppargc1a
Ppef1
Ppfia2
Ppil6
Ppm1a
Ppm1b
Ppm1d
Ppm1e
Ppm1g
Ppm1j
Ppox
Ppp1cc
Ppp1r10
Ppp1r11
Ppp1r16a
Ppp1r17
Ppp1r1c

Ppp1r2
Ppp1r27
Ppp1r32
Ppp1r36
Ppp1r3c
Ppp1r42
Ppp2r1b
Ppp2r2b
Ppp2r2d
Ppp2r3c
Ppp2r5c
Ppp2r5e
Ppp3cc
Ppp3r2
Ppp6c
Ppp6r1
Pradc1
Pram1
Prame
Prap1
Prdx4
Preb
Prelid3b
Prim2
Prkaa1
Prkag2
Prkar1b
Prkar2a
Prkca
Prkcd
Prkcg
Prkcq
Prkcz
Prkn
Prl
Prl2a1
Prl2b1
Prl7c1
Prm2
Prm3
Prmt8
Prob1
Prok2
Prom1
Prop1
Prpf31
Prph2

Prpsap1
Prr11
Prr18
Prr19
Prr22
Prr27
Prr32
Prr5l
Prr9
Prrc2a
Prrx1
Prrxl1
Prss21
Prss34
Prss37
Prss38
Prss39
Prss40
Prss42
Prss43
Prss44
Prss45
Prss46
Prss47
Prss52
Prss53
Prss54
Prss55
Prss58
Prtn3
Prune1
Prxl2a
Psd
Psd2
Psen1
Psip1
Psma8
Psmc3
Psmc3ip
Psmc5
Psmd13
Psme4
Psmf1
Psmg1
Pstpip2
Ptar1
Ptbp2

Ptchd3
Ptdss1
Ptdss2
Pten
Ptger3
Ptgfr
Pth1r
Pth2
Ptk6
Ptpa
Ptpdc1
Ptpmt1
Ptpn12
Ptpn20
Ptpn4
Ptpn7
Ptprk
Ptprn2
Ptprq
Ptprt
Ptrh1
Pttg1
Pusl1
Pwwp2b
Pwwp3a
Pxylp1
Pzp
Qrich1
Qrich2
R3hcc1
Rab11b
Rab11fip2
Rab11fip5
Rab15
Rab26
Rab28
Rab2b
Rab37
Rab39
Rab3b
Rab3c
Rab3gap2
Rab3il1
Rab44
Rab4a
Rab6b
Rabac1

Rabepk
Rabgap1l
Rad18
Rad21l
Rad23a
Rad51d
Rad54l
Rad9b
Rag2
Ralgps1
Ralgps2
Ralyl
Ranbp17
Ranbp2
Ranbp3l
Ranbp9
Rangap1
Rarb
Rarres1
Rars2
Rasal2
Rasgrf2
Rasip1
Rb1
Rbfox1
Rbm11
Rbm27
Rbm44
Rbm46
Rbm4b
Rbms3
Rbpjl
Rc3h1
Rcc1
Rchy1
Rcor3
Rcvrn
Rd3
Rdh11
Rdh12
Rdh14
Rdh8
Rdx
Rec8
Reep4
Reep6
Relt

Rerg
Retreg1
Retreg2
Rev3l
Rfc1
Rftn2
Rfx1
Rfx2
Rfx3
Rfx4
Rfx6
Rfx8
Rgl1
Rgs16
Rgs22
Rgs7
Rgs8
Rgsl1
Rhag
Rhbdd1
Rhbdd2
Rhbdd3
Rhcg
Rhno1
Rho
Rhot1
Rhov
Rhpn1
Ribc1
Ribc2
Rif1
Rimbp3
Rin1
Rint1
Riok3
Ripor3
Rlbp1
Rmdn2
Rmi1
Rmnd5b
Rnase12
Rnaseh2a
Rnf125
Rnf126
Rnf138
Rnf139
Rnf14

Rnf144a
Rnf151
Rnf169
Rnf19a
Rnf19b
Rnf20
Rnf207
Rnf220
Rnf224
Rnf24
Rnf32
Rnf38
Rnf39
Rnf4
Rnf40
Rnf41
Rnf43
Rnf44
Rnpep
Ropn1
Ropn1l
Ror1
Rp1l1
Rp2
Rp9
Rpain
Rpe65
Rpgr
Rpgrip1l
Rph3a
Rpl39l
Rpl3l
Rrnad1
Rrp12
Rrp15
Rrp8
Rsbn1
Rsph1
Rsph10b
Rsph14
Rsph4a
Rsph6a
Rsph9
Rspry1
Rtbdn
Rtf2
Rtkn2

Rtn4rl1
Rtn4rl2
Rttn
Rufy3
Runx1
Runx1t1
Runx2
Ruvbl2
Rwdd2a
Rwdd4a
Rxfp1
Ryr1
Ryr3
S100a7a
S100g
S100z
Saa4
Sag
Samd10
Samd13
Samd3
Samd4
Samd4b
Samd8
Sap130
Sarm1
Sass6
Satl1
Sav1
Saxo1
Saxo2
Saysd1
Sbds
Sbf2
Sbno1
Scaf4
Scamp2
Scand1
Scaper
Sccpdh
Scel
Scg5
Scgb1c1
Scgb3a1
Scgn
Sclt1
Scly

Scmh1
Scml4
Scn10a
Scn11a
Scn2a
Scn4a
Scn9a
Scnn1a
Scoc
Scp2d1
Scrg1
Scrn3
Scrt1
Scrt2
Sdf2l1
Sdk2
Sec14l1
Sec14l2
Sec14l3
Sec14l4
Sec14l5
Sec16a
Sec22a
Sec22c
Sec23a
Sec61a2
Sel1l2
Selenos
Selenov
Selenow
Sema3e
Sema4a
Sema4g
Senp2
Septin10
Septin12
Septin3
Septin4
Serac1
Serf1
Serp2
Serpina16
Serpinb2
Serpinb6b
Serpinb7
Serpine3
Serpini2

Sertad2
Setbp1
Setx
Sez6
Sez6l
Sez6l2
Sfmbt1
Sftpd
Sgca
Sgcb
Sgf29
Sgip1
Sgk1
Sgk2
Sgms1
Sgms2
Sgo1
Sgsm1
Sh2d2a
Sh2d4b
Sh2d6
Sh3bp2
Sh3gl3
Sh3glb1
Sh3rf2
Shank1
Shc3
Shcbp1l
Shisa3
Shisa7
Shisal2b
Shkbp1
Shld2
Shmt1
Siae
Siah2
Siglec15
Sik2
Sik3
Sil1
Sim1
Simc1
Sirt2
Sirt3
Sirt4
Sis
Sit1

Ska3
Sla2
Slain2
Slamf1
Slamf9
Slc10a4l
Slc10a5
Slc11a1
Slc12a3
Slc12a7
Slc13a3
Slc13a4
Slc13a5
Slc14a2
Slc15a1
Slc15a5
Slc16a12
Slc16a14
Slc16a3
Slc16a7
Slc16a8
Slc17a2
Slc17a6
Slc1a2
Slc22a1
Slc22a14
Slc22a16
Slc22a2
Slc22a20
Slc22a23
Slc22a5
Slc22a7
Slc23a2
Slc24a4
Slc25a20
Slc25a21
Slc25a35
Slc25a37
Slc25a39
Slc25a41
Slc25a54
Slc26a4
Slc26a5
Slc26a8
Slc28a3
Slc2a13
Slc2a3

Slc2a5
Slc2a7
Slc2a9
SLC30A3
Slc30a4
Slc33a1
Slc34a2
Slc34a3
Slc35a5
Slc35b4
Slc35e3
Slc35f1
Slc35f4
Slc35g1
Slc35g2
Slc35g3
Slc36a1
Slc36a3
Slc37a1
Slc37a2
Slc38a11
Slc38a3
Slc38a7
Slc38a9
Slc39a12
Slc39a3
Slc43a3
Slc44a5
Slc46a2
Slc4a1
Slc4a1ap
Slc4a5
Slc4a7
Slc4a8
Slc5a12
Slc66a2
Slc6a1
Slc6a20a
Slc6a20b
Slc6a4
Slc6a6
Slc6a7
Slc7a9
Slc8a1
Slc8a2
Slc8b1
Slc9a2

Slc9a3
Slc9a3r1
Slc9a4
Slc9b1
Slc9c1
Slco2a1
Slco5a1
Slfn14
Slfn5
Slfnl1
Slk
Sltm
Slurp2
Slx4ip
Smap1
Smarcb1
Smc1b
Smc5
Smc6
Smco1
Smco2
Smco3
Smco4
Smcr8
Smg5
Smg7
Smg9
Smim13
Smim22
Smim23
Smim24
Smim27
Smim8
Smim9
Smpd2
Smpd3
Smpd5
Snap29
Sncaip
SNORA70
SNORD116
Snrk
Snrpd1
Snta1
Sntb2
Sntg1
Sntn

Snupn
Snx13
Snx16
Snx19
Snx25
Snx3
Snx31
Socs7
Sord
Sort1
Sost
Sox30
Sox5
Sox6
Sp7
Sp8
Spa17
Spaca1
Spaca3
Spaca4
Spaca6
Spaca7
Spaca9
Spag1
Spag16
Spag17
Spag4
Spag5
Spag6
Spag6l
Spag8
Spag9
Spam1
Spast
Spata16
Spata17
Spata18
Spata19
Spata20
Spata25
Spata3
Spata31
Spata31d1b
Spata32
Spata33
Spata4
Spata45

Spata46
Spata5
Spata7
Spata9
Spatc1
Spatc1l
Spats1
Spats2
Spcs1
Spcs3
Spdya
Spef1
Spef1l
Spem1
Spem2
Spesp1
Spg7
Sphkap
Spink2
Spink8
Spire2
Spn
Spns3
Spo11
Spock3
Spp2
Sppl2b
Spred3
Sprtn
Spryd7
Spsb1
Spsb2
Spta1
Spty2d1
Sra1
Srd5a3
Srf
Srms
Srpk1
Srpk2
Srr
Srrd
Srrm3
Srrt
Srsf12
Ssmem1
Ssna1

Sstr2
Ssx2ip
St18
St6galnac2
St7
St8sia1
St8sia3
St8sia6
Stag3
Stam2
Stambp
Stard6
Stat4
Stil
Stimate
Stk17b
Stk19
Stk31
Stk32a
Stk32b
Stk33
Stk39
Stkld1
Stmn1
Stmn3
Stn1
Stox2
Stpg1
Stpg2
Stpg3
Stpg4
Strada
Strap
Strbp
Stt3b
Stx18
Stx19
Stx2
Stx5a
Stx8
Stxbp1
Styxl1
Suclg1
Suco
Sufu
Sugp2
Sugt1

Sult1b1
Sult1c1
Sult1c2
Sult6b2
Sun1
Sun3
Sun5
Supt20
Supt3
Supt6
Supv3l1
Susd5
Sv2b
Svep1
Svip
Svop
Syce1l
Syce3
Sycp1
Syngap1
Syngr3
Syngr4
Synj2
Synj2bp
Synpo2l
Synpr
Sypl
Sypl2
Syt10
Syt14
Syt16
Syt17
Syt2
Syt5
Sytl3
Sytl5
T
Taar4
Taar9
Tac2
Tacc3
Taf12
Tagln3
Taok1
Tas1r1
Tasor2
Tat

Tatdn3
Tbata
Tbc1d1
Tbc1d14
Tbc1d15
Tbc1d2
Tbc1d20
Tbc1d21
Tbc1d23
Tbc1d2b
Tbc1d30
Tbc1d32
Tbc1d7
Tbc1d9
Tbcel
Tbkbp1
Tbl2
Tbp
Tbpl1
Tbpl2
Tbr1
Tbx1
Tbx15
Tbx19
Tbx4
Tcaf3
Tcaim
Tcam1
Tcea2
Tceanc2
Tcf24
Tcfl5
Tchp
Tcp11
Tcp11x2
Tcte1
Tctex1d1
Tctex1d2
Tctn1
Tdp2
Tdrd1
Tdrd3
Tdrd5
Tdrd6
Tdrd7
Tdrd9
Tdrp

Tead1
Tek
Tekt1
Tekt2
Tekt3
Tekt4
Tekt5
Tenm1
Tenm2
Tenm4
Tent4b
Tent5b
Tent5c
Tepp
Terb2
Terf1
Tesk1
Tesk2
Tesmin
Tespa1
Tex13a
Tex13c1
Tex13c2
Tex13c3
Tex21
Tex22
Tex26
Tex28
Tex33
Tex35
Tex36
Tex37
Tex38
Tex43
Tex44
Tex45
Tex47
Tex48
Tex52
Tex55
Tex9
Tfam
Tfap2a
Tfap2b
Tfap2e
Tfb1m
Tfpi

Tg
Tgm4
TH
Thap2
Thap7
Thbs4
Theg
Thegl
Them5
Themis3
Thnsl1
Thoc5
Thoc7
Thsd7b
Thumpd3
Tigd2
Tigd4
Tigd5
Tigit
Timm13
Timm9
Tinag
Tkfc
Tktl2
Tlcd4
Tldc2
Tle2
Tle7
Tlk2
Tll1
Tlr5
Tm4sf19
Tm4sf20
Tm4sf4
Tm6sf2
Tm7sf2
Tmbim7
Tmc1
Tmc5
Tmc7
Tmco2
Tmco5
Tmco5b
Tmeff1
Tmeff2
Tmem102
Tmem107

Tmem108
Tmem120a
Tmem129
Tmem132c
Tmem134
Tmem138
Tmem140
Tmem144
Tmem145
Tmem150c
Tmem151a
Tmem156
Tmem161b
Tmem169
Tmem170b
Tmem174
Tmem175
Tmem183a
Tmem190
Tmem191c
Tmem198b
Tmem200a
Tmem200c
Tmem205
Tmem210
Tmem217
Tmem225
Tmem229b
Tmem231
Tmem232
Tmem239
Tmem241
Tmem243
Tmem246
Tmem247
Tmem248
Tmem255a
Tmem26
Tmem262
Tmem269
Tmem270
Tmem30c
Tmem39b
Tmem41b
Tmem45b
Tmem50b
Tmem53

Tmem54
Tmem63c
Tmem67
Tmem71
Tmem8
Tmem82
TMEM89
Tmem95
Tmf1
Tmprss11a
Tmprss11f
Tmprss11g
Tmprss12
Tmprss13
Tmprss3
Tmprss9
Tnfaip2
Tnfaip8
Tnfaip8l1
Tnfrsf11a
Tnfrsf13b
Tnfrsf8
Tnfsf11
Tnfsf15
Tnfsf18
Tnfsf8
Tnk1
Tnks
Tnmd
Tnn
Tnnc1
Tnnc2
Tnni3k
Tnp1
Tnp2
Tnr
Tnrc6c
Tns1
Tob1
Togaram1
Tom1
Tomm20l
Tomm70a
Top2a
Topaz1
Toporsl
Tor1aip1

Tor1aip2
Tox2
Tpcn2
Tpd52l2
Tpgs2
Tph2
Tpi1
Tpo
Tpp2
Tppp2
Tprg
Tprgl
Tprn
Tpst1
Traf1
Traf3ip1
Traip
Tram1
Tram1l1
Tram2
Treh
Trh
Trhde
Trhr
Trim11
Trim14
Trim17
Trim24
Trim27
Trim28
Trim29
Trim33
Trim36
Trim37
Trim39
Trim42
Trim45
Trim46
Trim55
Trim58
Trim61
Trim66
Trim69
Trim7
Trim72
Trim80
Triml1

Trip11
Trip12
Triqk
Troap
Trp53tg5
Trp63
Trpc6
Trpc7
Trpm6
Trpm8
Trpt1
Trpv1
Trpv3
Trpv5
Tsacc
Tsga10
Tsga13
Tshb
Tsks
Tsnaxip1
Tspan1
Tspan6
Tspoap1
Tspyl5
Tssc4
Tssk1
Tssk2
Tssk3
Tssk4
Tssk5
Tssk6
Tsx
Ttbk1
Ttc12
Ttc21a
Ttc21b
Ttc22
Ttc23
Ttc25
Ttc26
Ttc29
Ttc30a2
Ttc30b
Ttc32
Ttc39a
Ttc39d
Ttc41

Ttc7
Ttk
Ttll1
Ttll10
Ttll11
Ttll13
Ttll3
Ttll4
Ttll6
Ttll8
Tuba4a
Tuba8
Tubal3
Tubb3
Tubb4a
Tubb4b
Tubd1
Tulp2
Tulp4
Tusc3
Txlnb
Txndc8
Txndc9
Txnrd3
Tyk2
U1
U3
U6
Uaca
Ubac1
Ubap2
Ubash3a
Ubd
Ube2a
Ube2c
Ube2f
Ube2j1
Ube2k
Ube3b
Ube4a
Ubl3
Ubl4b
Ubl7
Ublcp1
Ubn1
Ubn2
Ubox5

Ubqln3
Ubqln5
Ubr4
Ubr5
Ubtd1
Ubxn10
Ubxn11
Ubxn2a
Ubxn4
Ubxn6
Ubxn8
Ucp3
Ufl1
Ufsp2
Uggt2
Uhmk1
Uhrf1
Uhrf1bp1l
Ulk2
Ulk4
Umod
Unc13a
Unc13c
Unc45b
Unc5c
Unc80
Uox
Upf2
Upk1a
Upk1b
Upk3a
Uqcc1
Uqcc2
Uqcrfs1
Uroc1
Use1
Usp1
Usp12
Usp15
Usp16
Usp2
Usp24
Usp25
Usp29
Usp3
Usp32
Usp37

Usp38
Usp42
Usp44
Usp47
Usp48
Usp49
Usp50
Usp7
Usp8
Vamp4
Vars
Vash2
Vasp
Vat1l
Vdac3
Vgll3
Vhl
Vinac1
Vip
Vipas39
Vipr2
Vmn2r1
Vmo1
Vps13a
Vps13b
Vps26a
Vps28
Vps45
Vps53
Vrk2
Vrk3
Vsig10l
Vsig8
Vsnl1
Vwa3a
Vwa3b
Vwa5b1
Wapl
Wbp11
Wbp2nl
Wdfy1
Wdfy4
Wdpcp
Wdr12
Wdr19
Wdr25
Wdr27

Wdr31
Wdr34
Wdr35
Wdr37
Wdr38
Wdr41
Wdr47
Wdr48
Wdr49
Wdr54
Wdr60
Wdr62
Wdr63
Wdr64
Wdr72
Wdr73
Wdr78
Wdr93
Wdr95
Wdtc1
Wee2
Wfdc15a
Wfdc3
Wnt1
Wnt10b
Wnt16
Wnt2
Wnt3
Wnt3a
Wwp1
Wwp2
Xirp2
Xk
Xkr7
Xkr8
Xkr9
Xpo4
Xpo5
Xpo6
Xpot
Xpr1
Xrcc6
Xrra1
Ybx2
Ybx3
Ydjc
Yif1b

Yipf7
Ylpm1
Yme1l1
Yod1
Ypel1
Ypel4
Zan
Zap70
Zbbx
Zbed4
Zbtb3
Zbtb32
Zbtb44
Zbtb48
Zbtb5
Zbtb8a
Zbtb8b
Zc2hc1a
Zc2hc1b
Zc2hc1c
Zc3h10
Zc3h12c
Zc3h12d
Zc3h14
Zc3h3
Zcchc9
Zdbf2
Zdhhc11
Zdhhc19
Zdhhc22
Zdhhc23
Zdhhc4
Zdhhc5
Zfand3
Zfand6
Zfhx4
Zfp1
Zfp157
Zfp160
Zfp174
Zfp185
Zfp217
Zfp267
Zfp286
Zfp287
Zfp330
Zfp35

Zfp365
Zfp367
Zfp37
Zfp385a
Zfp385c
Zfp41
Zfp420
Zfp438
Zfp451
Zfp454
Zfp462
Zfp488
Zfp511
Zfp512b
Zfp526
Zfp541
Zfp560
Zfp568
Zfp574
Zfp58
Zfp592
Zfp597
Zfp599
Zfp608
Zfp628
Zfp646
Zfp647
Zfp652
Zfp653
Zfp654
Zfp663
Zfp689
Zfp69
Zfp712
Zfp768
Zfp78
Zfp804a
Zfp804b
Zfp821
Zfp882
Zfp952
Zfr2
Zfyve1
Zfyve26
Zhx3
Zic5
Zkscan16

Zkscan17
Zkscan2
Zkscan7
Zmat4
Zmiz2
Zmynd10
Zmynd12
Zmynd15
Znhit2
Znrd1as
Zp3r
Zpbp
Zpbp2
Zpr1
Zranb1
Zscan2
Zswim1
Zswim3
Zswim6
Zup1

S vs W- down
0610010K14Rik
1110008P14Rik
1110012L19Rik
1110038F14Rik
1500009L16Rik
1600014C10Rik
1700017B05Rik
1810010H24Rik
2010300C02Rik
2210016F16Rik
2310011J03Rik
2310033P09Rik
2410002F23Rik
2410131K14Rik
2510009E07Rik
2610002M06Rik
2810459M11Rik
3110021N24Rik
4931406C07Rik
5730409E04Rik
5S_rRNA
6430548M08Rik
A4galt
Aard
Abat
Abca2
Abca6
Abcb10
Abcb4
Abcb7
Abcb8
Abcc1
Abcc4
Abcc5
Abcc6
Abcc8
Abcd3
Abcd4
Abcg1
Abcg2
Abhd15
Abhd3
Abhd6
Abhd8
Ablim1

Abracl
Abraxas1
Acaa2
Acad8
Acad9
Acadl
Acadm
Acads
Acbd4
Accs
Ackr1
Ackr3
Ackr4
Acly
Aco1
Acot4
Acot6
Acsbg1
Acsf2
Acsl4
Acss1
Acss3
Acta2
Actc1
Actn1
Acvr1
Acvr2b
Acvrl1
Acy3
Adam10
Adam23
Adam33
Adamts10
Adamts15
Adamts2
Adamts7
Adamtsl5
Adap1
Adcy2
Adcy4
Adcy6
Adcyap1r1
Adgra2
Adgra3
Adgrb1
Adgrb2
Adgre5

Adgrg1
Adgrl1
Adgrl2
Adgrl4
Adi1
Adipor1
Adora1
Adora2a
Adora2b
Adprh
Adprhl1
Adprhl2
Adra1b
Adra2c
Adrb2
Aebp1
Afap1l1
Afap1l2
Afm
Agap1
Agfg2
Agrn
Agt
Agtr2
Ahdc1
Ahr
Aifm1
Aifm3
Aig1
Ajap1
Ajuba
Ak3
Ak4
Akap17b
Akap7
Akr1b3
Akr1b8
Akr1c19
Akt1
Alad
Alcam
Aldh16a1
Aldh1a1
Aldh1a3
Aldh1b1
Aldh2
Aldh3b1

Aldh3b3
Aldh4a1
Aldh7a1
Aldoc
Alg1
Alkbh2
Alkbh7
Alox5ap
Alpk1
Als2cl
Amacr
Amer1
Amh
Amhr2
Amigo1
Amigo2
Ampd2
Ampd3
Anapc5
Angpt2
Angpt4
Angptl1
Angptl2
Angptl4
Angptl6
Angptl7
Ank
Ank1
Ank3
Ankdd1a
Ankrd46
Ano6
Ano8
Anp32b
Anp32e
Antxr2
Anxa1
Anxa11
Anxa2
Anxa4
Anxa6
Anxa7
Aoc3
Aox1
Ap1g2
Apba3
Apbb1

Apbb3
Apeh
Apex1
Apex2
Aph1a
Apmap
Apobec3
Apoc1
Apoc4
Apoe
Apold1
Apool
App
Aprt
Aqp1
Aqp3
Aqp4
Aqp5
Arap2
Arfip2
Arhgap1
Arhgap10
Arhgap15
Arhgap22
Arhgap27
Arhgap31
Arhgap4
Arhgap42
Arhgap44
Arhgap6
Arhgef10l
Arhgef15
Arhgef18
Arhgef19
Arhgef25
Arhgef28
Arhgef3
Arhgef39
Arhgef40
Arhgef5
Arhgef9
Arid5a
Arid5b
Arl14
Arl4c
Arl4d
Arl5a

Arl8a
Armc10
Armcx3
Armcx5
Armcx6
Arntl
Arpc1a
Arpc1b
Arpin
Arrdc2
Arrdc4
Arsb
Arsi
Arx
Asb2
Ascl2
Asic1
Asic2
Asic3
Asl
Asph
Atad3a
Atg10
Atg101
Atg14
Atg16l1
Atic
Atp11a
Atp13a2
Atp1a1
Atp1b2
Atp2a1
Atp2b1
Atp2b3
Atp5g2
Atp6ap1
Atp6v0a1
Atp6v0e
Atp8b1
Atp8b2
AU021092
Auh
Auts2
Avpi1
AW551984
Axin2
Axl

B230219D22Rik
B2m
B3galt6
B3gat3
B3glct
B3gnt2
B3gnt7
B3gnt9
B4galnt4
B4galt1
B4galt3
B4galt4
B4galt6
Baalc
Baat
Bace1
Bace2
Bach1
Bahd1
Baiap2
Baiap2l1
Bambi
Barx2
Basp1
Batf
Batf3
Bax
Bbc3
BC003965
BC048403
Bcam
Bckdhb
Bcl10
Bcl2
Bcl3
Bcl6
Bcl6b
Bcl7a
Bcl9
Bcl9l
Bcr
Bdh2
Bdkrb2
Bean1
Begain
Bend3
Bend4

Bex3
Bgn
Bhlhb9
Bhlhe40
Bhlhe41
Bicc1
Bicra
Bid
Bin1
Blnk
Bloc1s4
Blvra
Bmf
Bmp1
Bmp4
Bmp5
Bmp7
Bmyc
Bnc1
Bnip3
Boc
Bok
Bphl
Bst2
Btbd2
Btbd3
Btbd6
Btg2
Btk
C1qa
C1qb
C1qbp
C1qc
C1ql1
C1ql3
C1qtnf1
C1qtnf2
C1qtnf3
C1qtnf6
C1qtnf7
C1rl
C2cd2l
C330007P06Rik
C3ar1
C6
C7
C8g

Cables1
Cabp7
Cacna2d1
Cacna2d2
Cacnb3
Cad
Cadm3
Cald1
Calhm2
Calhm5
Camk1d
Camk2g
Camk2n1
Camkk1
Camkk2
Cand2
Capg
Capn2
Capn6
Car11
Car13
Car2
Car7
Card11
Card6
Carmil1
Carmil3
Cask
Caskin1
Casp1
Casp2
Casp6
Casp7
Casp8
Casq2
Castor2
Cat
Cav1
Cav2
Cav3
Cavin1
Cavin2
Cavin3
Cbfa2t3
Cbfb
Cbr3
Cbx3

Cbx4
Ccbe1
Ccdc102a
Ccdc120
Ccdc149
Ccdc17
Ccdc22
Ccdc28b
Ccdc51
Ccdc6
Ccdc8
Ccdc80
Ccdc85b
Ccer2
Cckbr
Ccl11
Ccl5
Ccl8
Ccm2
Ccn2
Ccna2
Ccnd1
Ccnd2
Ccnf
Ccr2
Ccr4
Ccr5
Cd14
Cd151
Cd163
Cd200
Cd244a
Cd248
Cd24a
Cd276
Cd300a
Cd302
Cd34
Cd36
Cd3d
Cd44
Cd47
Cd53
Cd63
Cd68
Cd74
Cd83

Cd9
Cd93
Cd99l2
Cda
Cdc16
Cdc25b
Cdc42ep1
Cdc42ep2
Cdc6
Cdca7l
Cdh1
Cdh15
Cdh19
Cdh23
Cdh24
Cdh3
Cdh5
Cdh6
Cdhr5
Cdk2ap2
Cdk3
Cdk5
Cdkn1a
Cdkn1b
Cdkn2aipnl
Cdo1
Cdon
Cdt1
Cdx1
Cdyl2
Cebpb
Cebpd
Cenpb
Cep170b
Cers2
Cers4
Cers5
Ces1a
Cfap73
Cfd
Cfdp1
Ch25h
Chad
Chadl
Chic1
Chp2
Chpf2

Chrd
Chrdl1
Chrna2
Chrna5
Chst11
Chst12
Chst2
Chst3
Chst7
Chst8
Cilp2
Cisd3
Cish
Cited1
Cited2
Cited4
Ckap4
Ckb
Cks1b
Clcn2
Clcn4
Clcnka
Cldn1
Cldn11
Cldn23
Cldn3
Cldn5
Clec12b
Clec14a
Clec3b
Clic1
Clic5
Clip2
Clmp
Cln3
Cln5
Cln6
Clstn1
Clta
Cltb
Clu
Clybl
Cmc1
Cmc4
Cmip
Cmtm6
Cmtm8

Cmtr2
Cndp2
Cnga2
Cnih1
Cnksr3
Cnn2
Cnn3
Cnnm1
Cnnm3
Cnp
Cnpy2
Cnpy3
Cnpy4
Cnrip1
Cntfr
Cntn3
Coa5
Coa6
Coa7
Coch
Col11a2
Col18a1
Col1a1
Col1a2
Col27a1
Col3a1
Col4a2
Col4a4
Col4a5
Col4a6
Col5a3
Col6a1
Col6a2
Col8a2
Colec12
Colgalt1
Colgalt2
Comt
Comtd1
Copz2
Coq10a
Coro1a
Coro1b
Coro1c
Coro2b
Cotl1
Cox14

Cox4i2
Cox7a1
Cox7a2l
Cox7b
Cox8a
Cpe
Cpn2
Cpne2
Cpne3
Cpne6
Cpox
Cpt1a
Cpt1c
Cpxm1
Cpz
Crabp1
Cracr2b
Creb3
Creb3l1
Creb3l2
Creg1
Crhr1
Crhr2
Crim1
Crispld1
Crispld2
Crlf1
Crocc
Crtc1
Crtc2
Crtc3
Crybg1
Crygn
Cryl1
Csad
Csdc2
Csf1r
Csk
Csn3
Csnk1e
Cspg5
Csrp1
Csrp2
Cst12
Cst3
Cstb
Cstdc1

Cstdc2
Cstf2
Ctbp1
Ctcfl
Ctdsp1
Ctdspl
Ctnna1
Ctnnal1
Ctrb1
Ctsa
Ctsb
Ctsc
Ctsh
Ctss
Ctsz
Cttn
Ctu1
Cuedc1
Cul7
Cuta
Cux2
Cx3cl1
Cx3cr1
Cxcl12
Cyb561d1
Cyb5a
Cyb5r3
Cyba
Cyc1
Cyfip1
Cygb
Cyp11b1
Cyp11b2
Cyp1b1
Cyp21a1
Cyp26b1
Cyp27a1
Cyp2d22
Cyp2j6
Cyp2s1
Cyp2u1
Cyp4b1
Cyp4v3
Cyp4x1
Cyp7b1
Cyth4
D17H6S53E

D1Ertd622e
D2hgdh
D630045J12Rik
Daam2
Dag1
Dao
Dapk1
Dapk2
Dapk3
Dapp1
Dbi
Dbp
Dcaf12l1
Dcaf4
Dcakd
Dck
Dclk2
Dcn
Dcp2
Dcps
Dctd
Dctpp1
Ddah1
Ddah2
Ddit4
Ddr1
Ddr2
Ddx3x
Decr1
Decr2
Def6
Def8
Degs1
Dele1
Dennd2d
Dennd3
Dennd6a
Depp1
Des
Dgkk
Dgkq
Dhcr24
Dhcr7
Dhh
Dhrs13
Dhrs3
Dhrs7

Dhrs9
Dhx37
Dipk1b
Diras2
Disp1
Dkk3
Dlg5
Dlk1
Dlk2
Dmrta1
Dmtn
Dmwd
Dnajc19
Dnase1l1
Dnase2a
Dnd1
Dnlz
Dnmbp
Dnmt3b
Dnph1
Doc2b
Doc2g
Dock1
Dock10
Dock11
Dock5
Dock9
Dok1
Dok2
Dok3
Dok7
Dpagt1
Dpp4
Dpp6
Dpy19l3
Dpysl2
Dpysl4
Dram2
Drd4
Drp2
Dsc2
Dse
Dstn
Dtd2
Dtna
Dtwd2
Dtx1

Dusp2
Dusp23
Dusp26
Dusp28
Dusp7
Dusp8
Dusp9
Dvl3
Dync1i1
Dynlrb1
Dysf
E2f1
E330021D16Rik
Ebf4
Ebp
Ece1
Ecel1
Echdc3
Echs1
Eci1
Ecm1
Ecm2
Ecscr
Eda2r
Edn1
Edn3
Ednra
Ednrb
Eed
Eef1e1
Efcc1
Efemp2
Efhd2
Efna1
Efna2
Efna5
Efnb1
Efs
Egfl7
Egflam
Egfr
Egr1
Egr2
Egr4
Ehd2
Ehd3
Ehd4

Ehf
Ehmt1
Ei24
Eid1
Eid2
Eif2ak3
Eif2b1
Eif2s3x
Eif4a1
Eif4ebp2
Elac2
Elmsan1
Eln
Elovl7
Elp1
Emilin1
Eml1
Eml4
Emp1
Emp2
Emp3
Emx1
Emx2
Enc1
Endod1
Enho
Eno2
Enox2
Enpep
Enpp1
Enpp2
Ensa
Entpd2
Entpd3
Eogt
Epas1
Epb41
Epb41l1
Epb41l3
Epdr1
Epha1
Epha2
Ephb4
Ephb6
Ephx2
Epn3
Erap1

Erbb2
Ercc6l
Erg
Erg28
Ergic1
Ero1l
Esam
Esr2
Esrp1
Esrrb
Esyt2
Esyt3
Etfa
Etnk2
Ets2
Etv5
Eva1a
Eva1b
Evc
Evc2
Evpl
Exo5
Exoc3l
Exoc3l2
Exosc6
Exosc7
Extl1
Extl3
Ezh1
Ezr
F11r
F13a1
F2r
F2rl1
F3
F8a
Fabp3
Fabp4
Fabp5
Fadd
Fads1
Fads2
Fads3
Fads6
Fah
Fahd1
Fam102a

Fam107a
Fam110b
Fam114a1
Fam120c
Fam122b
Fam129b
Fam131c
Fam13a
Fam149a
Fam160b2
Fam168b
Fam171a1
Fam174c
Fam180a
Fam193b
Fam20a
Fam20c
Fam234b
Fam241b
Fam3a
Fam3c
Fam43a
Fam50a
Fam53a
Fancb
Fancf
Fancl
Farp1
Fas
Fat1
Faxc
Fbl
Fbln1
Fbln2
Fbn1
Fbrsl1
Fbxl19
Fbxl4
Fbxl6
Fbxl8
Fbxo10
Fbxo16
Fbxo21
Fbxo31
Fbxo32
Fbxo44
Fbxo45

Fbxo5
Fbxw11
Fcer1g
Fcgrt
Fcsk
Fermt3
Ffar2
Fgd1
Fgd2
Fgd5
Fgf1
Fgf11
Fgf13
Fgf18
Fgfr1
Fgfr2
Fgfr3
Fgfr4
Fgl2
Fgr
Fh1
Fhl1
Fhl2
Fhl3
Fhod1
Fibin
Fign
Fignl2
Fitm2
Fkbp10
Fkbp11
Fkbp14
Fkbp5
Fkbp9
Fkrp
Fli1
Flna
Flot2
Flrt1
Flt3l
Fmnl1
Fmo5
Fn1
Fnbp1
Folr2
Fos
Fosl2

Foxa2
Foxc1
Foxd3
Foxe1
Foxf1
Foxf2
Foxj2
Foxn2
Foxn3
Foxo1
Foxo3
Foxo4
Foxo6
Foxp4
Foxred2
Foxs1
Frmd4a
Frmd5
Frmd7
Frmd8
Frzb
Fscn1
Fshr
Fst
Fstl1
Fundc1
Fundc2
Fuom
Furin
Fut7
Fxyd1
Fxyd5
Fxyd6
Fxyd7
Fyb
Fyb2
Fyn
Fzd1
Fzd10
Fzd2
Fzd5
Fzd6
Fzd7
Fzd8
G0s2
Gab1
Gab3

Gabarapl2
Gabrq
Gadd45a
Gadd45b
Gadd45g
Gal3st3
Gal3st4
Gale
Galk1
Galnt1
Galnt12
Galnt15
Galnt17
Galnt6
Galnt9
Galntl6
Galr3
Galt
Gamt
Gar1
Garnl3
Gas1
Gas6
Gas7
Gask1a
Gata1
Gata4
Gata6
Gatd1
Gatm
Gba
Gbe1
Gbgt1
Gbp2
Gbp3
Gc
Gca
Gcdh
Gcnt4
Gdf10
Gdi1
Gdnf
Gdpd2
Gfi1b
Gfra1
Gfra2
Gfra4

Gga2
Ggcx
Ggps1
Ggta1
Ghdc
Ghr
Gigyf1
Gimap1
Gimap4
Gimap6
Gimap8
Gipc3
Gja1
Gja3
Glb1
Gldc
Glg1
Gli1
Gli2
Gli3
Glis1
Glis2
Glis3
Glmp
Glo1
Glrb
Glrx
Gltp
Gm12248
Gm13889
Gm1673
Gm20390
Gm22077
Gm22842
Gm23187
Gm23212
Gm23991
Gm24907
Gm25791
Gm25835
Gm26247
Gm26330
Gm27308
Gm27505
Gm27533
Gm27538
Gm27636

Gm27786
Gm27861
Gm28006
Gm45717
Gm45902
Gm49909
Gmds
Gmeb1
Gmpr
Gna11
Gna14
Gna15
Gnat1
Gnb1l
Gnb4
Gnb5
Gne
Gng11
Gng12
Gng13
Gng4
Gng7
Gnl3l
Golim4
Got2
Gpam
Gpatch1
Gpc1
Gpc3
Gpc4
Gpd1
Gpkow
Gpnmb
Gpr137b
Gpr146
Gpr156
Gpr157
Gpr161
Gpr173
Gpr176
Gpr37
Gpr62
Gpr63
Gpr75
Gpsm1
Gpx1
Gpx3

Gpx7
Gpx8
Gramd1a
Gramd3
Grb14
Grb2
Grcc10
Grhpr
Grik4
Grip2
Grk2
Grk5
Grk6
Grpel2
Grtp1
Gse1
Gsg1l
Gsn
Gspt2
Gsr
Gsta3
Gsta4
Gstm1
Gstm2
Gstm6
Gstm7
Gsto1
Gstp3
Gtf2ird2
Gucy1a1
Gulp1
Gxylt2
Gypc
Gys1
H2-Ea
H2-Eb1
H2-Ob
H2-T24
H2aj
H6pd
Hap1
Hbegf
Hbp1
Hcfc1
Hcn3
Hcrtr1
Hdac5

Hdac6
Hdac7
Hdac8
Hdc
Hddc2
Heatr3
Hebp1
Hebp2
Helb
Helz2
Heph
Herc3
Hes1
Hes7
Hexb
Hexim1
Hey2
Heyl
Hgfac
Hgsnat
Hhatl
Hhex
Hibadh
Hibch
Hic1
Higd1b
Hikeshi
Hilpda
Hlcs
Hlf
Hlx
Hmg20b
Hmgb3
Hmgcl
Hmgcs2
Hmgn3
Hmox1
Hmx1
Hmx2
Hmx3
Hnmt
Hnrnpa1
Hnrnph2
Hnrnpr
Hoga1
Homer2
Homer3

Hopx
Hoxa10
Hoxa5
Hoxa6
Hoxa7
Hoxa9
Hoxb13
Hoxb2
Hoxb4
Hoxb6
Hoxb7
Hoxb8
Hoxc13
Hoxc6
Hoxd10
Hoxd4
Hoxd9
Hpcal1
Hpgd
Hpn
Hprt
Hps1
Hps6
Hpx
Hr
Hrh1
Hs3st1
Hs3st4
Hs6st1
Hs6st2
Hsd11b1
Hsd11b2
Hsd17b1
Hsd17b10
Hsd17b14
Hsd17b3
Hsd3b7
Hsf4
Hsp90ab1
Hspa12b
Hspb1
Hspb2
Hspb7
Hspb8
Hspg2
Htra1
Htra3

Hyal2
Hyi
Iah1
Icam1
Icam4
Icam5
Icosl
Id1
Id2
Id3
IDH1
Idh2
Idh3g
Ids
Ier3
Ier5l
Ifi209
Ifi35
Ifitm10
Ifitm3
Ifnar1
Ifngr1
Ifngr2
Igbp1
Igdcc3
Igdcc4
Igf1
Igf1r
Igf2bp1
Igf2bp2
Igf2r
Igfbp3
Igfbp6
Igfbp7
Igfbpl1
Ighm
Igsf3
Ikbip
Ikbke
Il10ra
Il12rb1
Il13ra1
Il15ra
Il17d
Il17ra
Il17rc
Il18bp

Il25
Il33
Il6ra
Ildr2
Ilvbl
Imp3
Impdh1
Inf2
Inha
Inhbb
Inka2
Inpp5b
Inpp5d
Inpp5f
Inppl1
Insig1
Insr
Insyn1
Irak1
Irak2
Irf2
Irf2bp1
Irf2bpl
Irf3
Irf6
Irf7
Irf8
Irf9
Irgm1
Irgm2
Irgq
Irs1
Irs2
Irs3
Irx4
Islr
Ism2
Isoc1
Itga1
Itga3
Itga5
Itga6
Itga7
Itga8
Itgav
Itgb1
Itgb4

Itgb5
Itgb7
Itgb8
Itm2a
Itm2b
Itm2c
Itpk1
Itpr3
Ivd
Jade1
Jade2
Jag1
Jag2
Jagn1
Jakmip1
Jam2
Jam3
Jdp2
Josd2
Jph2
Jtb
Jun
Junb
Jund
Kalrn
Kank1
Kank2
Kank4
Kat14
Kat2b
Kazald1
Kbtbd2
Kcna2
Kcna7
Kcnab3
Kcnb1
Kcnc4
Kcng1
Kcng3
Kcng4
Kcnh2
Kcnip1
Kcnip3
Kcnj11
Kcnj5
Kcnj8
Kcnk1

Kcnk3
Kcnmb4
Kcnn1
Kcnq1
Kcns1
Kcns2
Kcns3
Kcnt1
Kctd12
Kctd12b
Kctd15
Kctd17
Kctd6
Kdelr1
Kdm1b
Kdm4b
Kdm5c
Kdm6a
Kdm8
Kdr
Kel
Kera
Khnyn
Kif12
Kif13b
Kif1a
Kif1c
Kif21a
Kirrel3
Kit
Kitl
Klf10
Klf12
Klf13
Klf15
Klf16
Klf2
Klf6
Klhdc8a
Klhdc8b
Klhl13
Klhl2
Klhl21
Klhl26
Klhl29
Klhl33
Klhl36

Klhl40
Klhl5
Klk14
Krt23
L1td1
L3hypdh
L3mbtl3
Lad1
Lag3
Lama1
Lama2
Lama4
Lama5
Lamb1
Lamb2
Lamc1
Lamc3
Lamp2
Lamtor1
Lao1
Lap3
Laptm4b
Large1
Larp6
Las1l
Lasp1
Lbhd2
Lbp
Lbx1
Lcat
Lcn2
Lcp1
Ldb2
Ldb3
Ldhb
Ldlr
Ldlrad3
Ldlrap1
Ldoc1
Lemd2
Lenep
Leprot
Lfng
Lgals1
Lgals3
Lgals3bp
Lgr4

Lhcgr
Lhfp
Lima1
Limd1
Lims2
Lin28a
Lipa
Lipg
Lipm
Lipt2
Litaf
Llgl1
Llgl2
Lman1
Lmnb1
Lmo1
Lmo4
Lmod1
Lmx1b
Lonp1
Lonrf2
Lpar3
Lpar6
Lpcat1
Lpcat2
Lpl
Lratd2
Lrch1
Lrfn3
Lrig3
Lrp10
Lrp3
Lrp4
Lrp5
Lrp8
Lrrc14
Lrrc17
Lrrc32
Lrrc39
Lrrc4
Lrrc4b
Lrrc75a
Lrrc75b
Lrrc8d
Lrrn1
Lrrn2
Lsm14a

Lsm6
Lsm7
Lsp1
Lsr
Lss
Lta4h
Ltb
Ltbp1
Ltbp3
Ltbr
Ltv1
Luc7l3
Lum
Ly6e
Ly6h
Ly6m
Ly86
Lypd1
Lysmd2
Lyve1
Lztr1
Lzts2
Lzts3
Madd
Maf
Mafa
Mafb
Maff
Mafk
Mag
Mageb16
Maged1
Maged2
Mageh1
Magix
Magt1
Mal2
Mamdc2
Mamdc4
Man1a
Man1b1
Man1c1
Maneal
Maoa
Maob
Map1s
Map2k3

Map3k1
Map3k20
Map3k21
Map3k8
Map4k2
Map4k4
Map4k5
Mapk12
Mapk13
Mapk14
Mapk1ip1l
Mapk3
Mapkapk2
Mapre1
Mapre2
Mapt
Marchf9
Marcks
Marcksl1
Mark4
Marveld1
Marveld2
Mast3
Matk
Matn2
Mb21d2
Mblac1
Mblac2
Mbnl1
Mbnl2
Mbnl3
Mboat2
Mbp
Mbtps1
Mcam
Mccc2
Mcm2
Mcm3
Mcm6
Mcoln1
Mcts1
Mcu
Mdfi
Mdfic
Mdh2
Med12
Med16

Med20
Mef2a
Mef2c
Mef2d
Meis1
Meis2
Mertk
Mesd
Met
Metrn
Mettl14
Mettl26
Mettl27
Mettl9
Mex3a
Mfap2
Mfap4
Mfap5
Mfge8
Mfng
Mfsd1
Mfsd10
Mfsd13a
Mfsd14b
Mfsd2a
Mfsd4b4
Mfsd7a
Mgat4b
Mgmt
Mgp
Mgrn1
Mgst1
Mgst2
Mgst3
Mical1
Mical2
Mid1ip1
Mief2
Mier2
Mif
Minar2
Mir143
Mir145a
Mir202
Mir214
Mirlet7b
Mirlet7c-2

Mkx
Mllt3
Mllt6
Mlx
Mmab
Mmd
Mmd2
Mmp11
Mmp14
Mmp15
Mmp2
Mmp23
Mmrn1
Mmrn2
Mn1
Mnt
Mob2
Mob3c
Mocs1
Mocs2
Morf4l2
Mospd1
Mov10
Mpeg1
Mpnd
Mpp1
Mpp2
Mpp3
Mpp7
Mpped1
Mpv17
Mpv17l2
Mpzl2
Mr1
Mras
Mri1
Mro
Mrpl16
Mrpl18
Mrpl23
Mrpl24
Mrpl28
Mrpl40
Mrpl54
Mrpl57
Mrps28
Mrtfa

Mrto4
Mrvi1
Ms4a4a
Msantd2
Msantd3
Msantd4
Msc
Msh2
Msi1
Msmo1
Msn
Mst1
Mt2
Mt3
Mta3
Mtap
Mtarc1
Mtarc2
Mtch1
Mtcl1
Mtcp1
Mthfd1
Mtln
Mtrf1l
Mtss1
Mtss2
Mturn
Mustn1
Mvb12b
Mvp
Mx1
Mx2
Mxd4
Mxra7
Mxra8
Mybpc3
Myc
Mycn
Myct1
Myd88
Myh11
Myh6
Myh7
Myh7b
Myh9
Myl6b
Mylip

Mylk
Mymk
Myo1f
Myo6
Myo7a
Myom2
Mypop
Myrf
Myrip
Myzap
N4bp1
N4bp3
Naa10
Naaa
Nab1
Nab2
Nacc2
Nadsyn1
Naglu
Nampt
Nanos1
Nanos2
Nanos3
Napsa
Nat8f1
Nav2
Nbdy
Nbeal2
Nbl1
Ncam1
Ncf1
Nck2
Nckap5l
Ncor2
Ncs1
Nde1
Ndfip1
Ndn
Ndp
Ndrg1
Ndst1
Ndufa1
Ndufa4
Ndufb11
Necap1
Nectin1
Nectin2

Nedd4
Nedd9
Nefh
Neil1
Neil2
Nek7
Nek8
Nenf
Neo1
Nes
Neu2
Neurl1b
Neurl2
Nfam1
Nfatc1
Nfatc4
Nfe2l2
Nfia
Nfib
Nfil3
Nfkb1
Nfkb2
Nfkbia
Nfkbid
Nfx1
Ngfr
Ngrn
Nhlrc1
Nhs
Nhsl1
Nid1
Nif3l1
Nin
Ninj1
Ninj2
Nipa1
Nipal2
Nipsnap1
Nipsnap2
Nkain1
Nkain4
Nkap
Nkd1
Nkpd1
Nkx1-1
Nkx3-1
Nlrp5

Nlrx1
Nmral1
Nmrk1
Nol3
Nol6
Nol9
Notch1
Notch2
Notch3
Notch4
Notum
Nox1
Nox4
Noxa1
Npcd
Npdc1
Npff
Npl
Npnt
Nppc
Npr1
Npr2
Nprl2
Nptx1
Npy1r
Nqo2
Nr0b1
Nr0b2
Nr1d1
Nr2f1
Nr2f2
Nr2f6
Nr3c2
Nr5a1
Nradd
Nrarp
Nrbp2
Nrep
Nrgn
Nrip1
Nrp1
Nrp2
Nrros
Nsdhl
Nsmce4a
Nsun2
Nt5c2

Nt5dc2
Nt5e
Ntf5
Nthl1
Ntn4
Ntrk1
Nuak2
Nucks1
Nudt14
Nudt5
Nup205
Nup210
Nupr1
Nxnl2
Nxph3
Nyap1
Nynrin
Oaf
Obsl1
Ogn
Ogt
Olfm1
Olfml1
Olfml2a
Olfml2b
Oma1
Orai1
Orai3
Osbpl7
Osr1
Osr2
Ostc
Otop2
Otud5
Otulinl
Otx1
Oxct1
P2rx4
P2rx6
P2ry1
P2ry2
P2ry6
P3h2
P3h3
P3h4
P4ha1
P4ha2

Pabpc4
Pabpc4l
Pacc1
Padi2
Pak1
Pak4
Pald1
Pam
Pank1
Pank4
Panx2
Papss1
Paqr4
Pard3
Pard6b
Pard6g
Parm1
Parp16
Parp4
Pars2
Parva
Parvb
Parvg
Patz1
Pawr
Pax7
Pax8
Pbx1
Pbx2
Pbx3
Pccb
Pcdh18
Pcdh19
Pcdh7
Pced1b
Pcgf2
Pck2
Pcolce2
Pcsk1n
Pcyox1
Pcyox1l
Pdcd2
Pde8b
Pde9a
Pdf
Pdgfa
Pdgfb

Pdgfc
Pdgfd
Pdgfra
Pdgfrb
Pdha1
Pdia2
Pdia4
Pdk1
Pdk4
Pdlim3
Pdlim4
Pdrg1
Pdxk
Pdyn
Pdzd11
Pdzrn3
Pea15a
Pear1
Pecam1
Pecr
Pepd
Per2
Perm1
Pex26
Pex6
Pex7
Pf4
Pfas
Pfkfb1
Pfkfb3
Pgap3
Pgd
Pgghg
Pgls
Pgm2
Pgpep1
Pgr
Pgrmc1
Phb2
Pheta1
Phf13
Phka1
Phka2
Phlda1
Phlda3
Phox2a
Phtf2

Phyh
Phyhd1
Phyhip
Phykpl
Piezo1
Piezo2
Pik3ip1
Pik3r1
Pik3r4
Pik3r5
Pim2
Pim3
Pip4k2a
Pip4k2b
Pip4p2
Pip5k1c
Pir
Pitpnm3
Pitx1
Piwil4
Pja1
Pkd2
Pkdcc
Pkia
Pkn1
Pkn3
Pla2g15
Pla2g1b
Pla2g4a
Pla2g5
Pla2g7
Pla2r1
Plaat3
Plau
Plcd3
Plcg1
Pld2
Plek2
Plekha7
Plekhg1
Plekhg3
Plekhg5
Plekhg6
Plekhh1
Plekhh3
Plgrkt
Plk2

Plk3
Plod1
Plod2
Plod3
Plp1
Plpp2
Plpp3
Plpp4
Plpp7
Plppr1
Plppr4
Plppr5
Pls3
Plscr2
Plscr3
Plscr4
Pltp
Plvap
Plxdc1
Plxdc2
Plxna1
Plxna3
Plxnc1
Plxnd1
Pm20d1
Pmepa1
Pmm1
Pmp22
Pmvk
Pnkd
Pnma2
Pnma3
Pnmt
Pnpla1
Pnpla6
Pnpo
Podn
Podxl
Pofut1
Poglut1
Poglut2
Poglut3
Pola1
Polm
Polr1b
Polr2c
Polr2h

Polr3g
Pomgnt2
Pomk
Pon2
Pon3
Pop5
Porcn
Postn
Pou2f2
Pou3f2
Pou6f1
Ppan
Ppcs
Ppfia4
Ppib
Ppic
Ppif
Ppl
Ppm1f
Ppm1m
Ppp1r13l
Ppp1r14a
Ppp1r14d
Ppp1r1b
Ppp1r3d
Ppp1r3f
Ppp1r3g
Ppp1r9a
Ppp2ca
Ppp2r1a
Ppp2r5b
Ppt2
Pqbp1
Pqlc3
Prag1
Prcd
Prdm1
Prdm11
Prdm5
Prdx3
Prelid2
Prelid3a
Prelp
Prep
Prex1
Prickle1
Prickle3

Prkacb
Prkag3
Prkcb
Prkd1
Prkra
Prmt3
Prmt6
Prnp
Prodh
Proser1
Proser2
Prps2
Prr12
Prr7
Prrc1
Prrc2b
Prrt1
Prrt3
Prss12
Prss35
Prss50
Psap
Psen2
Psma7
Psmb9
Psmd10
Psmg4
Pstk
Ptafr
Ptbp1
Ptch1
Pter
Ptger1
Ptger4
Ptges2
Ptgis
Ptk2b
Ptk7
Ptms
Ptn
Ptov1
Ptp4a3
Ptpn3
Ptpn5
Ptpn6
Ptpn9
Ptprc

Ptprcap
Ptprd
Ptpre
Ptprf
Ptprg
Ptprj
Ptprm
Ptprs
Ptpru
Pttg1ip
Pvalb
Pxdc1
Pxdn
Pxn
Pycard
Pycr1
Pycr2
Pygb
Pygm
Pygo1
Pyroxd2
Qprt
R3hdm4
Rab11fip4
Rab13
Rab17
Rab20
Rab27a
Rab29
Rab33b
Rab34
Rab35
Rab39b
Rab3d
Rab5b
Rab6a
Rab9
Rabggta
Rabggtb
Rac1
Rac2
Rac3
Rad21
Rad9a
Radx
Rai1
Rai2

Ralb
Ralbp1
Raly
Ramp2
Rap1a
Rap1gap2
Rap2b
Rap2c
Rapgef3
Rapgef4
Rapgefl1
Rara
Rarg
Rarres2
Rasa3
Rasa4
Rasd1
Rasd2
Rasef
Rasgrp1
Rasgrp2
Rasgrp3
Rasl10a
Rasl10b
Rasl11a
Rasl11b
Rasl12
Rassf10
Rassf3
Rassf5
Rassf8
Raver2
Rbak
Rbbp5
Rbbp7
Rbbp9
Rbck1
Rbfox3
Rbm10
Rbm15b
Rbm24
Rbm3
Rbm38
Rbm41
Rbm43
Rbm47
Rbms2

Rbmx
Rbp1
Rbp4
Rbpms2
Rcan1
Rcan2
Rcan3
Rcc1l
Rcc2
Rcn1
Rcn3
Rcor2
Rcsd1
Reck
Recql4
Reep3
Reep5
Rell1
Rem1
Rem2
Ret
Retreg3
Rex1bd
Rfc5
Rflna
Rftn1
Rgma
Rgmb
Rgs10
Rgs11
Rgs14
Rgs18
Rgs19
Rgs2
Rgs6
Rgs7bp
Rhbdf1
Rhbdf2
Rhbdl1
Rhbdl3
Rhob
Rhobtb2
Rhoc
Rhod
Rhof
Rhoj
Rhoq

Rhox12
Rhpn2
Ric8a
Ric8b
Rilp
Rilpl1
Rilpl2
Rimbp2
Rimkla
Rims4
Ripk1
Ripk3
Ripk4
Rita1
Rnase4
Rnase6
Rnasel
Rnd3
Rnf122
Rnf130
Rnf145
Rnf152
Rnf180
Rnf208
Rnf227
Rnh1
Rnpepl1
Robo2
Rom1
Ror2
Rorc
Rpl23
Rpl32
Rpp14
Rpp25
Rpp38
Rpp40
Rps12
Rps17
Rps18
Rps3
Rps4x
Rps5
Rps6ka3
Rps6ka4
Rps6ka6
Rps6kb2

Rps8
Rrad
Rragb
Rras
Rras2
Rsad1
Rsbn1l
Rspo1
Rspo2
Rspo4
Rsrp1
Rsu1
Rtl5
Rtn4r
Rufy4
Rundc3b
Runx3
Rxra
Ryk
S100a10
S100a13
S100a16
S100a4
S100a6
S1pr2
S1pr3
Sac3d1
Sall2
Sall4
Samd14
Sap25
Sap30l
Sapcd2
Sardh
Sars
Sars2
Sash3
Sat1
Sat2
Satb1
Sbk1
Sbk2
Sbno2
Scaf11
Scamp5
Scap
Scara3

Scara5
Scarb1
Scarb2
Scarf1
Scarf2
Scd2
Scd3
Scn1b
Scpep1
Scrn2
Sct
Scx
Scyl1
Sdc1
Sdc2
Sdc4
Sdr42e1
Sdr9c7
Sds
Sdsl
Sec11c
Sec24d
Sectm1a
Selenom
Selenon
Selenop
Sell
Selp
Sema3c
Sema3f
Sema3g
Sema4b
Sema4c
Sema4d
Sema4f
Sema5a
Sema5b
Sema6a
Sema6c
Sema6d
Sema7a
Septin1
Septin14
Septin8
Septin9
Serinc1
Serinc2

Serinc5
Serpina12
Serpinb8
Serpine1
Serpine2
Serping1
Serpinh1
Serpini1
Sertad4
Sesn1
Setd6
Sfn
Sfrp1
Sft2d2
Sfxn1
Sfxn2
Sfxn3
Sfxn5
Sgce
Sgpl1
Sgsm3
Sh2b1
Sh2d4a
Sh3bgr
Sh3bgrl
Sh3bgrl2
Sh3bp4
Sh3bp5
Sh3d19
Sh3pxd2a
Sh3tc1
Sh3tc2
Shank2
Shank3
Shbg
Shc2
She
Shf
Shfl
Shh
Shisa2
Shisa5
Shisa8
Shisal1
Shmt2
Shtn1
Sigirr

Siglec1
Sigmar1
Sipa1
Six1
Ski
Skp2
Slain1
Slc10a3
Slc10a6
Slc12a9
Slc15a3
Slc16a1
Slc16a11
Slc16a13
Slc16a2
Slc16a9
Slc19a1
Slc19a2
Slc19a3
Slc1a3
Slc1a4
Slc20a1
Slc22a17
Slc22a18
Slc25a10
Slc25a11
Slc25a12
Slc25a13
Slc25a14
Slc25a18
Slc25a22
Slc25a23
Slc25a27
Slc25a29
Slc25a43
Slc25a44
Slc25a48
Slc25a5
Slc27a1
Slc27a2
Slc27a3
Slc27a4
Slc27a6
Slc29a1
Slc29a2
Slc29a4
Slc2a1

Slc2a4
Slc2a6
Slc30a10
Slc30a2
Slc31a2
Slc32a1
Slc35a4
Slc35b2
Slc35c1
Slc35d2
Slc35e2
Slc35e4
Slc35f2
Slc36a4
Slc37a3
Slc37a4
Slc38a1
Slc38a2
Slc38a5
Slc39a14
Slc39a5
Slc39a7
Slc40a1
Slc41a1
Slc44a1
Slc44a2
Slc47a1
Slc47a2
Slc48a1
Slc49a4
Slc4a11
Slc4a2
Slc4a3
Slc5a3
Slc5a5
Slc6a9
Slc7a10
Slc7a4
Slc7a5
Slc8a3
Slc9a3r2
Slc9a6
Slc9a7
Slc9b2
Slco2b1
Slco4a1
Slfn2

Slit3
Smad1
Smad3
Smad4
Smad7
Smad9
Smarcd3
Smc1a
Smim1
Smim10l2a
Smim11
Smim20
Smim26
Smo
Smox
Smpdl3a
Smpdl3b
Sms
Smtnl2
Smug1
Smyd1
Smyd3
Smyd5
Snai2
Snap91
Snca
Sncb
Sncg
Sned1
Snn
Snora16a
Snora44
SNORA66
Snora68
SNORA70
Snord123
SNORD14
SNORD25
SNORD26
SNORD31
SNORD33
SNORD34
Snord35a
Snord35b
SNORD36
Snord38a
SNORD39

Snord45c
Snord47
Snord49a
Snord65
SNORD81
Snord89
Snrpe
Sntb1
Snx10
Snx12
Snx15
Snx18
Snx21
Snx24
Snx33
Snx5
Snx6
Socs3
Socs5
Sod3
Soga1
Sohlh1
Sohlh2
Sorbs1
Sorl1
Sowahb
Sowahc
Sox1
Sox13
Sox15
Sox18
Sox21
Sox3
Sox4
Sox8
Sox9
Sp3
Sp5
Sparc
Sparcl1
Speg
Sphk1
Spi1
Spin2c
Spin4
Spint1
Spns2

Spock2
Spon1
Spred2
Spry1
Spry2
Spry4
Spryd3
Spsb4
Sptb
Sptbn1
Srgap3
Sri
Srm
Srpx
Srxn1
Ss18l1
Ssbp1
Ssbp3
Ssbp4
Ssh1
Ssr2
Ssr4
Sstr3
St3gal1
St3gal2
St3gal3
St3gal4
St6gal2
St6galnac4
St6galnac6
St8sia5
Stab1
Stac3
Stag2
Stambpl1
Stap2
Stard3
Stard3nl
Stard8
Stat3
Stat5a
Stat5b
Stat6
Stbd1
Stc2
Steap1
Steap2

Stk24
Stk26
Stk38l
Stoml2
Ston1
Stra6
Stra8
Stradb
Stx1a
Stx1b
Stx4a
Stx6
Stx7
Stxbp6
Styk1
Suclg2
Sulf2
Sult1a1
Sult4a1
Sult5a1
Sumf1
Sumf2
Surf6
Susd2
Susd3
Susd4
Suv39h1
Sv2a
Svil
Swap70
Syde1
Syde2
Syn2
Sync
Syne4
Syngr1
Syngr2
Synpo
Syt1
Syt12
Syt13
Syt9
Sytl4
Tab1
Taf1c
Taf3
Taf7

Taf7l
Tafa5
Tagln
Tagln2
Tal1
Taok3
Tap1
Tap2
Tapbp
Tapbpl
Tarbp1
Tax1bp3
Taz
Tbc1d10b
Tbc1d16
Tbc1d25
Tbc1d8
Tbc1d9b
Tbcd
Tbck
Tbl1x
Tbx18
Tbx2
Tbx3
Tbx6
Tbxa2r
Tbxas1
Tcaf1
Tcap
Tcea3
Tceal1
Tceal9
Tcf15
Tcf21
Tcf23
Tcf3
Tcf7l2
Tcim
Tcirg1
Tcof1
Tead4
Tec
Tecr
Ten1
Tent5a
Tep1
Terf2ip

Tesc
Tet2
Tfap2c
Tfap4
Tfeb
Tfrc
Tgfbi
Tgfbr2
Tgif2
Tgm1
Tgm2
Tgs1
Tha1
Thap3
Thbd
Thbs2
Themis2
Thnsl2
Thoc2
Thoc6
Thra
Thrb
Thsd4
Thsd7a
Thy1
Thyn1
Ticam1
Tifa
Timm8a1
Timp2
Timp3
Tinagl1
Tjp3
Tkt
Tle1
Tle4
Tln1
Tlnrd1
Tlr2
Tlr3
Tlr4
Tlr9
Tm2d3
Tm4sf1
Tm7sf3
Tmc6
Tmc8

Tmcc2
Tmed1
Tmed9
Tmem100
Tmem11
Tmem114
Tmem115
Tmem119
Tmem120b
Tmem121b
Tmem130
Tmem132a
Tmem132e
Tmem14c
Tmem150a
Tmem151b
Tmem159
Tmem160
Tmem161a
Tmem164
Tmem17
Tmem176a
Tmem184b
Tmem186
Tmem200b
Tmem204
Tmem223
Tmem238
Tmem240
Tmem245
Tmem25
Tmem256
Tmem29
Tmem35a
Tmem38a
Tmem43
Tmem45a
Tmem47
Tmem50a
Tmem51
Tmem62
Tmem63b
Tmem69
Tmem86a
Tmem88b
Tmem91
Tmem98

Tmlhe
Tmprss2
Tmprss6
Tmsb4x
Tmub1
Tnfrsf11b
Tnfrsf12a
Tnfrsf14
Tnfrsf19
Tnfrsf1a
Tnfrsf1b
Tnfrsf21
Tnfrsf25
Tnfsf9
Tnfsfm13
Tnip1
Tnk2
Tnks1bp1
Tnni1
Tnni3
Tnrc18
Tns2
Tnxb
Tom1l2
Tomm40l
Tomm5
Tor1a
Tor1b
Tor2a
Tor4a
Tox
Tox3
Tpbg
Tpbgl
Tpcn1
Tpd52
Tpd52l1
Tpm1
Tpm2
Tpm3
Tpm4
Tpmt
Tpp1
Tpst2
Trabd
Trabd2b
Tradd

Traf5
Traf6
Traf7
Trak1
Trak2
Trank1
Trem2
Trex1
Trib1
Trib2
Trib3
Tril
Trim15
Trim2
Trim21
Trim25
Trim26
Trim3
Trim35
Trim47
Trim56
Trim6
Trim62
Trim71
Trim8
Trim9
Trip6
Trmt2b
Trnp1
Trp53
Trp53bp2
Trp53i11
Trpc4
Trpm4
Trps1
Trpv2
Trpv4
Trrap
Tsc22d4
Tsen15
Tsen34
Tshz1
Tshz3
Tsku
Tspan13
Tspan14
Tspan15

Tspan17
Tspan18
Tspan3
Tspan32
Tspan33
Tspan4
Tspan7
Tspyl2
Tsr2
Tst
Tstd1
Ttc13
Ttc3
Ttc38
Ttc9
Ttl
Ttyh1
Ttyh2
Ttyh3
Tub
Tubb6
Tuft1
Tulp3
Tvp23a
Twf2
Twist1
Twist2
Twistnb
Txlna
Txn1
Txndc16
Txnrd2
Tyro3
Tyrobp
U6
Uap1l1
Uba7
Ubl4a
Ubr2
Ubtf
Uchl1
Uckl1
Ucp2
Ulk3
Unc119
Unc13d
Unc5b

Unc93b1
Uncx
Ung
Unk
Uqcr11
Urah
Uri1
Urod
Uros
Usf1
Usf2
Ush1g
Usp18
Usp27x
Usp28
Usp35
Usp36
Usp43
Usp46
Utf1
Utp11
Utp14a
Uxt
Vamp5
Vamp7
Vamp8
Vangl1
Vars2
Vash1
Vasn
Vat1
Vav1
Vbp1
Vegfa
Vegfb
Vegfc
Vegfd
Vezf1
Vgll2
Vgll4
Vill
Vim
Vldlr
Vma21
Vnn1
Vopp1
Vps26b

Vps37d
Vps50
Vsig10
Vstm4
Vstm5
Vwa1
Vxn
Was
Wasf3
Wasl
Wbp1
Wbp1l
Wdr4
Wdr43
Wdr45
Wdr55
Wdr81
Wdr82
Wdr86
Wdsub1
Wfdc1
Wfdc5
Wfikkn1
Wfikkn2
Whrn
Wif1
Wipf3
Wls
Wnk2
Wnt10a
Wnt2b
Wnt4
Wnt5a
Wnt6
Wnt7b
Wnt9a
Wrnip1
Wsb2
Wscd1
Wt1
Wwc1
Wwtr1
Xaf1
Xylb
Xylt1
Xylt2
Yaf2

Yap1
Yars2
Yipf1
Ywhab
Ywhag
Ywhah
Zadh2
Zbed3
Zbp1
Zbtb12
Zbtb14
Zbtb16
Zbtb20
Zbtb24
Zbtb38
Zbtb39
Zbtb45
Zbtb7a
Zbtb7b
Zbtb7c
Zc3h12a
Zc3h7b
Zc3hav1
Zc3hav1l
Zc4h2
Zcchc24
Zcchc3
Zdhhc14
Zdhhc15
Zdhhc16
Zdhhc8
Zeb2
Zfand2b
Zfhx2
Zfp113
Zfp12
Zfp146
Zfp180
Zfp2
Zfp229
Zfp266
Zfp280b
Zfp280c
Zfp296
Zfp316
Zfp341
Zfp358

Zfp36
Zfp362
Zfp36l2
Zfp385b
Zfp395
Zfp414
Zfp422
Zfp449
Zfp46
Zfp467
Zfp503
Zfp521
Zfp553
Zfp566
Zfp575
Zfp579
Zfp580
Zfp593
Zfp618
Zfp619
Zfp64
Zfp641
Zfp651
Zfp667
Zfp668
Zfp683
Zfp688
Zfp691
Zfp692
Zfp703
Zfp704
Zfp707
Zfp740
Zfp777
Zfp784
Zfp786
Zfp799
Zfp827
Zfp846
Zfp866
Zfp867
Zfp868
Zfp869
Zfp90
Zfp92
Zfp961
Zfpm1

Zfpm2
Zfx
Zfyve21
Zfyve27
Zglp1
Zhx2
Zkscan3
Zmat1
Zmym3
Zmynd11
Zscan21
Zscan26
Zswim5
Zswim8
Zxdb
Zxdc

W+ vs W- up
0610030E20Rik
1110002E22Rik
1110017D15Rik
1110032A03Rik
1110051M20Rik
1500009C09Rik
1700001K19Rik
1700001L19Rik
1700001O22Rik
1700001P01Rik
1700003F12Rik
1700007K13Rik
1700008O03Rik
1700009N14Rik
1700010I14Rik
1700011L22Rik
1700012B07Rik
1700012B09Rik
1700012P22Rik
1700013F07Rik
1700015G11Rik
1700016C15Rik
1700016D06Rik
1700016H13Rik
1700016K19Rik
1700017N19Rik
1700018B08Rik
1700019A02Rik
1700019D03Rik
1700019N19Rik
1700020A23Rik
1700020L24Rik
1700020N01Rik
1700021F07Rik
1700022I11Rik
1700024G13Rik
1700028J19Rik
1700028P14Rik
1700029H14Rik
1700029I15Rik
1700029J07Rik
1700030J22Rik
1700030K09Rik
1700034E13Rik
1700034J05Rik

1700037C18Rik
1700037H04Rik
1700042G07Rik
1700057G04Rik
1700066M21Rik
1700067K01Rik
1700067P10Rik
1700074P13Rik
1700088E04Rik
1700092M07Rik
1700093K21Rik
1700094D03Rik
1700102P08Rik
1700109H08Rik
1700113H08Rik
1700122O11Rik
1700125H20Rik
1810013L24Rik
1810024B03Rik
2010106E10Rik
2200002J24Rik
2210408I21Rik
2300009A05Rik
2310061I04Rik
2610301B20Rik
2610318N02Rik
2900092C05Rik
3425401B19Rik
4430402I18Rik
4833427G06Rik
4921504E06Rik
4921507P07Rik
4921517D22Rik
4921536K21Rik
4921539E11Rik
4930402F06Rik
4930402H24Rik
4930404N11Rik
4930407I10Rik
4930415O20Rik
4930430F08Rik
4930444P10Rik
4930451I11Rik
4930452B06Rik
4930453N24Rik
4930505A04Rik
4930519G04Rik

4930522H14Rik
4930544D05Rik
4930550C14Rik
4930558K02Rik
4930568D16Rik
4930578I06Rik
4930579F01Rik
4930579G24Rik
4930590J08Rik
4931406B18Rik
4931428F04Rik
4931429L15Rik
4933402J07Rik
4933402N03Rik
4933405L10Rik
4933405O20Rik
4933417A18Rik
4933424G06Rik
4933428M09Rik
4933430I17Rik
5031439G07Rik
5330417C22Rik
5730480H06Rik
6330403K07Rik
6820408C15Rik
9330159F19Rik
9530053A07Rik
9530077C05Rik
9930012K11Rik
9930021J03Rik
A2ml1
A430005L14Rik
A930017K11Rik
A930018P22Rik
AA467197
Aaas
Aadacl2fm3
Aadacl4fm4
Aadat
Aars
Aasdhppt
Abca14
Abca15
Abca16
Abca17
Abca4
Abcb11

Abcc12
Abcf2
Abhd16b
Abhd2
Abhd5
Ablim2
Acap1
Acap3
Ace
Ace3
Acer2
Acot7
Acp4
Acp7
Acr
Acrbp
Acrv1
Acsbg2
Acsbg3
Acsl1
Acsl3
Acsl5
Acsl6
Acsm3
Acsm4
Acte1
Actl11
Actl7a
Actl7b
Actl9
Actn3
Actr3b
Actr6
Actrt1
Actrt2
Actrt3
Acvr1c
Acyp1
Adad1
Adad2
Adal
Adam11
Adam12
Adam18
Adam2
Adam24
Adam28

Adam3
Adam32
Adam5
Adamts3
Adamts4
Adamts6
Adamtsl3
Adarb2
Adat2
Adcy10
Adgrf4
Adgrf5
Adgrg7
Adig
Adrb1
Adrb3
Adtrp
Afap1
Aff2
Afg1l
Agbl2
Agbl4
Agbl5
Agfg1
Ago2
Ago3
Ago4
Agpat1
Agpat2
Agrp
Agtpbp1
Agxt
Ahcyl2
Ahi1
Ahrr
Ahsg
AI661453
Aipl1
Ajm1
Ak1
Ak7
Ak8
Ak9
Akap1
Akap10
Akap3
Akap4

Akna
Aknad1
Akr1e1
Aldh1a2
Aldoa
Aldob
Alk
Alkbh3
Allc
Alpk2
Alx1
Ambn
Amelx
Amer2
Ammecr1l
Amn1
Amz2
Angel1
Angptl8
Ankar
Ankef1
Ankib1
Ankmy1
Ankrd27
Ankrd29
Ankrd31
Ankrd34a
Ankrd34c
Ankrd35
Ankrd37
Ankrd42
Ankrd45
Ankrd53
Ankrd54
Ankrd55
Ankrd60
Ankrd61
Ankrd9
Anks4b
Ankzf1
Anln
Ano10
Ano2
Ano4
Ano5
Ano7
Antxrl

Aopep
Ap1m1
Ap2b1
Ap3b1
Ap3m2
Ap5b1
Apba1
Apip
Apobec2
Apoh
Aptx
Aqp11
Aqp12
Aqp7
Aqp9
Arc
Arel1
Arfgap3
Arfgef2
Arg1
Arg2
Arhgap19
Arhgap24
Arhgap29
Arhgap33
Arhgap9
Arhgef33
Arid3a
Arid3b
Arih1
Arih2
Arl13a
Arl13b
Arl14epl
Arl2bp
Arl3
Arl6
Arl6ip6
Arl9
Armc12
Armc3
Armc4
Armc9
Armh1
Armh4
Armt1
Arntl2

Arrdc5
Arsa
Art3
Asah2
Asap1
Asap3
Asb1
Asb10
Asb14
Asb15
Asb17
Asb3
Asb4
Asb5
Ascc2
Asf1b
Ash2l
Asna1
Aspscr1
Asrgl1
Astn1
Atad1
Atad2b
Ate1
Atg9a
Atg9b
Atl3
Atp10a
Atp10b
Atp1a4
Atp1b3
Atp4a
Atp6v1c1
Atp6v1c2
Atp6v1d
Atp6v1e2
Atp6v1fnb
Atp6v1h
Atp8b3
Atp8b5
Atp9a
Atp9b
Atr
Atxn2l
Atxn3
Atxn7l1
Atxn7l3b

Aurka
Avpr1b
AW554918
Awat2
Axdnd1
Azgp1
Azin2
B230118H07Rik
B4galnt2
B4galnt3
B9d1
B9d2
Babam2
Bag1
Bag3
Bag5
Bag6
Baiap3
Banf2
Bap1
Batf2
Baz2b
Bbof1
Bbs1
Bbs10
Bbs2
Bbs5
Bbs7
Bbs9
BC004004
BC048562
BC048671
Bcan
Bcap29
Bcat1
Bcl11a
Bcl2l12
Bcl2l14
Bcl2l15
Bco1
Bco2
Bdh1
Bhmt2
Bin2
Bin3
Birc5
Blk

Blzf1
Bnip5
Boll
Bpi
Bpifa1
Bpifa3
Bpifc
Braf
Brap
Brd2
Brdt
Brinp2
Bsn
Btaf1
Btbd1
Btbd10
Btbd16
Btbd18
Btbd19
Btbd9
Btg4
Btrc
Bub1b
C2cd3
C2cd6
C87436
C9orf72
Cab39l
Cabcoco1
Cabp2
Cabyr
Cacna1e
Cacna1h
Cacng2
Cacng7
Cacng8
Cacul1
Cage1
Calhm1
Calhm3
Calm1
Calm3
CALML3
Calr3
Caly
Camk4
Camkmt

Camkv
Caml
Camsap1
Cap2
Capn11
Capsl
Capza3
Capzb
Car1
Car10
Car6
Carf
Carnmt1
Casc1
Casq1
Catip
Catsper1
Catsper2
Catsper3
Catsper4
Catsperd
Catsperz
Cbl
Cbll1
Cby1
Cby2
Cby3
Cc2d2b
Ccdc103
Ccdc105
Ccdc106
Ccdc110
Ccdc113
Ccdc114
Ccdc116
Ccdc117
Ccdc134
Ccdc136
Ccdc14
Ccdc146
Ccdc148
Ccdc15
Ccdc150
Ccdc151
Ccdc154
Ccdc155
Ccdc157

Ccdc158
Ccdc159
Ccdc163
Ccdc169
Ccdc171
Ccdc173
Ccdc175
Ccdc178
Ccdc18
Ccdc180
Ccdc181
Ccdc182
Ccdc184
Ccdc186
Ccdc187
Ccdc189
Ccdc192
ccdc198
Ccdc24
Ccdc27
Ccdc30
Ccdc33
Ccdc34
Ccdc38
Ccdc39
Ccdc42
Ccdc59
Ccdc60
Ccdc62
Ccdc63
Ccdc65
Ccdc70
Ccdc74a
Ccdc77
Ccdc81
Ccdc88a
Ccdc88b
Ccdc89
Ccdc91
Ccdc92
Ccdc93
Ccdc9b
Ccer1
Cchcr1
Ccin
Ccl1
Ccl2

Ccl24
Ccl26
Ccna1
Ccnb2
Ccnh
Ccnk
Ccno
Ccnyl1
Ccp110
Ccpg1os
Cct3
Cct4
Cct5
Cct6b
Cd164l2
Cd209e
Cd37
Cd3eap
Cd46
Cdc123
Cdc14a
Cdc14b
Cdc25c
Cdc42ep3
Cdc45
Cdca2
Cdca3
Cdca5
Cdca8
Cdh10
Cdh4
Cdhr2
Cdhr3
Cdhr4
Cdiptos
Cdk18
Cdk20
Cdk5r1
Cdk5rap2
Cdk5rap3
Cdkal1
Cdkl2
Cdkl4
Cdkl5
Cdkn2aip
Cdkn2b
Cdkn2c

Cdkn3
Cdr2
Cdr2l
Cdrt4
Cds1
Cdv3
Cebpg
Cela2a
Celf3
Celf5
Celf6
Celsr2
Celsr3
Cenpc1
Cenpe
Cenph
Cenpj
Cenpt
Cenpu
Cep104
Cep126
Cep128
Cep135
Cep152
Cep162
Cep164
Cep19
Cep295
Cep295nl
Cep350
Cep41
Cep55
Cep57
Cep57l1
Cep63
Cep70
Cep72
Cep76
Cep83
Cep85l
Cep89
Cep95
Cep97
Cers3
Ces2a
Cetn1
Cetn3

Cetn4
Cfap100
Cfap126
Cfap157
Cfap161
Cfap206
Cfap221
Cfap298
Cfap299
Cfap300
Cfap36
Cfap410
Cfap43
Cfap44
Cfap45
Cfap52
Cfap54
Cfap57
Cfap58
Cfap65
Cfap69
Cfap70
Cfap77
Cfap97
Cfap97d1
Cfap97d2
Cfap99
Cfl2
Cftr
Cgas
Cgrrf1
Chaf1a
Chaf1b
Chchd6
Chd5
Chdh
Chfr
Chid1
Chil5
Chn1
Chn2
Chpt1
Chrm2
Chrm3
Chrm4
Chrm5
Chrna1

Chrna3
Chrnb1
Chrnb3
Chrne
Chst13
Ciapin1
Cib4
Cip2a
Cisd1
CK137956
Ckap2l
Cklf
Cks2
Clba1
Clca2
Cldn18
Cldn34a
Cldnd2
Clec16a
Clec2m
Clec4n
Clgn
Clhc1
Clip4
Clmn
Clnk
Clp1
Clstn2
Clvs1
Clvs2
Cmpk2
Cmya5
CN725425
Cnbd2
Cndp1
Cnga3
Cnga4
Cnih2
Cnnm4
Cnot10
Cntd1
Cntln
Cntn2
Cntn4
Cntrl
Cntrob
Cog1

Cog6
Cog8
Coil
Col16a1
Col22a1
Col2a1
Col5a1
Col9a3
Colq
Comp
Copg2
Cops5
Coq10b
Coq4
Coq9
Coro2a
Cox6b2
Cpa5
Cpeb2
Cpeb3
Cplx1
Cpm
Cpn1
Cpne4
Cpne9
Cps1
Cptp
Cpvl
Crat
Creb3l3
Creld2
Crem
Crip3
Cript
Crisp2
Crmp1
Cryab
Cryba2
Cryzl1
Csf3r
Csgalnact1
Csmd1
Csmd2
Csmd3
Csnk1g1
Csnk1g2
Csnk2a2

Csnk2b
Cspg4
Csrnp3
Cst10
Cst13
Cst8
Cstf1
Cstl1
Ctag2
Ctbs
Ctf1
Ctnna3
Ctsf
Cul3
Cux1
Cuzd1
Cwf19l2
Cyb5r1
Cyb5r2
Cyct
Cylc1
Cyp19a1
Cyp26c1
Cyp3a13
Cyp4f40
Cyp51
D130043K22Rik
D16Ertd472e
D1Pas1
D430042O09Rik
D7Ertd443e
Dalrd3
Dap
Dap3
Dbf4
Dbil5
Dcaf1
Dcaf10
Dcaf12
Dcaf5
Dcaf6
Dcdc2a
Dchs1
Dclk1
Dclk3
Dcp1b
Dcst1

Dcst2
Dctn2
Dctn4
Dcun1d3
Dcun1d4
Dcx
Dda1
Ddhd1
Ddx20
Ddx25
Ddx28
Ddx39
Ddx4
Ddx52
Dedd2
Defb14
Defb29
Defb50
Degs2
Dennd1b
Depdc1a
Depdc1b
Dera
Desi1
Deup1
Dgat1
Dgat2
Dgka
Dgke
Dgkh
Dgki
Dhrs11
Dhrs7b
Dhx16
Dhx34
Dhx58
Diablo
Diaph3
Dis3l
Dis3l2
Disp3
Dkkl1
Dlat
Dlec1
Dlgap5
Dll3
Dlx6

Dmac2l
Dmp1
Dmrt2
Dmrta2
Dmrtb1
Dmxl2
Dnaaf1
Dnaaf3
Dnaaf5
Dnah1
Dnah10
Dnah11
Dnah17
Dnah2
Dnah3
Dnah5
Dnah6
Dnah8
Dnaic1
Dnaic2
Dnajb13
Dnajb14
Dnajb2
Dnajb3
Dnajb4
Dnajb5
Dnajb7
Dnajb8
Dnajb9
Dnajc1
Dnajc15
Dnajc18
Dnajc21
Dnajc24
Dnajc27
Dnajc28
Dnajc3
DNAJC5B
Dnajc5g
Dnal1
Dnal4
Dnali1
Dnase1l3
Dnhd1
Dnm1l
Dnm2
Donson

Dop1a
Dot1l
Dpep3
Dpf3
Dpp7
Dpp8
Dpy19l1
Dpy19l2
Dpy30
Dpys
Dr1
Dram1
Drap1
Drc1
Drc3
Drc7
Drd3
Drosha
Dscaml1
Dsg2
Dsg3
Dtd1
Dupd1
Dusp10
Dusp13
Dusp15
Dusp18
Dusp21
Dusp4
Dydc1
Dydc2
Dym
Dync1h1
Dync2h1
Dync2li1
Dynlrb2
Dyrk1b
Dyrk3
Dyrk4
Dzip1
E2f5
E2f8
Ebf2
Eef2kmt
Eefsec
Efcab1
Efcab10

Efcab12
Efcab15
Efcab2
Efcab3
Efcab5
Efcab6
Efcab7
Efcab9
Efemp1
Efhb
Efhc1
Efhc2
Efhd1
Efna3
Efnb3
Egfem1
Egln2
Egr3
Ehd1
Eif2b5
Eif4g3
Elavl4
Elf2
Elf5
Elfn2
Ell2
Elmod1
Elmod2
Elovl4
Emc3
Emc7
Eme1
Eml6
Emsy
Enkd1
Enkur
Eno3
Eno4
Enpp3
Enthd1
Entr1
Ep400
Epc1
Epcam
Epha6
Epha7
Epsti1

Epx
Eqtn
Ergic2
Eri2
Erich2
Erich3
Erlin2
Ero1lb
Esm1
Espl1
Esrrg
Etfrf1
Etv1
Etv2
Exd1
Exd2
Exoc3l4
Extl2
Eya1
Eya3
Eya4
Fa2h
Faah
Faap100
Fabp6
Fads2b
Faf1
Faf2
Faim2
Faiml
Fam104a
Fam107b
Fam110a
Fam118a
Fam122c
Fam126a
Fam126b
Fam149b
Fam151b
Fam161a
Fam166a
Fam166b
Fam166c
Fam170a
Fam170b
Fam174b
Fam178b

Fam183b
Fam186a
Fam186b
Fam187a
Fam187b
Fam193a
Fam205c
Fam209
Fam214b
Fam216a
Fam217a
Fam219a
Fam221a
Fam221b
Fam227a
Fam227b
Fam228a
Fam228b
Fam229a
Fam229b
Fam243
Fam50b
Fam71a
Fam71d
Fam71e1
Fam71f1
Fam71f2
Fam76a
Fam78a
Fam81b
Fam83c
Fam83e
Fam92a
Fam98a
Fancd2os
Fancg
Fank1
Far2
Fars2
Fbn2
Fbp1
Fbxl13
Fbxl15
Fbxo15
Fbxo24
Fbxo27
Fbxo34

Fbxo36
Fbxo39
Fbxo4
Fbxo43
Fbxw10
Fbxw5
Fcgr1
Fdxacb1
Fem1b
Fem1c
Fen1
Fer
Fer1l5
Fer1l6
Fez1
Fga
Fgd3
Fgf21
Fgf9
Fgfbp1
Fgg
Fhad1
Fhdc1
Fhl5
Filip1l
Fis1
Fkbp1b
Fkbp4
Fkbp6
Fkbpl
Flacc1
Flcn
Flrt2
Flywch2
Fmc1
Fmn1
Fndc11
Fndc3a
Fndc3b
Fndc8
Fnip1
Fnta
Fosl1
Foxd2
Foxg1
Foxh1
Foxj1

Foxm1
Frat2
Frem1
Frs3
Fscn2
Fscn3
Fsip1
Fsip2
Fsip2l
Fstl5
Ftmt
Fxr1
Fyco1
Fyttd1
Fzr1
G6pc3
Gabbr1
Gabbr2
Gabra4
Gabrb1
Gabrg2
Gabrr1
Gabrr2
Galc
Galk2
Galnt14
Galnt3
Galntl5
Galp
Gan
Gapdhs
Garem2
Gas8
Gata2
Gbf1
Gcc1
Gcnt1
Gcnt3
Gcsam
Gde1
Gdf2
Gdf6
Gdpd4
Gemin5
Gen1
Gfod2
Gfpt1

Gfy
Ggct
Ggh
Ggn
Ggnbp1
Ggnbp2
Ghitm
Gid4
Gins2
Gins3
Gipr
Git1
Gja5
Gk2
Gkap1
Glb1l
Glb1l2
Glcci1
Gldn
Glipr1l2
Glra3
Gls2
Glt1d1
Glt6d1
Glyctk
Gm11437
Gm11634
Gm11992
Gm12695
Gm128
Gm136
Gm17018
Gm17359
Gm17949
Gm20403
Gm27301
Gm27747
Gm27834
Gm27884
Gm28729
Gm3045
Gm35339
Gm36368
Gm42906
Gm45521
Gm4869
Gm4952

Gm50318
Gm5134
Gm5142
Gm527
Gm5460
Gm5767
Gm614
Gm6569
Gm6583
Gm6657
Gm973
Gm9999
Gmcl1
Gmfg
Gmnn
Gmpr2
Gmps
Gnao1
Gnat3
Gnb3
Gng3
Gngt2
Gnpat
Gnpnat1
Golga4
Golga7b
Golgb1
Gorasp2
Got1l1
Gp5
Gpank1
Gpat4
Gpatch2
Gpd2
Gpr12
Gpr137
Gpr15
Gpr153
Gpr18
Gpr19
Gpr3
Gpr45
Gpr61
Gpr65
Gps1
Gpx6
Gramd1b

Gramd1c
Grap2
Grhl1
Gria2
Grid2ip
Grik2
Grin2b
Grin3a
Grk3
Grk4
Grm8
Grn
Gsdma
Gsg1
Gstcd
Gstm5
Gsto2
Gstt2
Gstt4
Gtf2a1l
Gtf2f2
Gtf3c4
Gtpbp2
Gtsf1
Gtsf1l
Gtsf2
Guca1a
Gucy1b1
Gulo
Gxylt1
Gys2
H1f6
H2ac12
H2ac21
H2aw
H2bc1
H2bc14
Habp2
Habp4
Hagh
Haghl
Hao1
Hat1
Haus1
Haus4
Hcfc2
Hdac1

Hddc3
Hdx
Heatr4
Heatr9
Hectd4
Hecw1
Hecw2
Hemgn
Henmt1
Hephl1
Herc1
Hexa
Hey1
Hgd
Hgs
Hif1an
Hipk3
Hipk4
Hk1
Hk3
Hmcn1
Hmga2
Hnf1b
Hnf4g
Homez
Hook1
Hook2
Hormad1
Hormad2
Hoxb1
Hoxc8
Hoxc9
Hpca
Hpcal4
Hps4
Hpse2
Hrh4
Hrob
Hsbp1l1
Hsdl2
Hsf5
Hsfy2
Hspa13
Hspa1l
Hspa4l
Hspb6
Hspb9

Htr2c
Htra4
Hus1b
Hyal3
Hyal4
Hyal5
Hyal6
Hydin
Hykk
Hyls1
Hyou1
Ica1l
Id4
Ide
Ifih1
Ift122
Ift172
Ift20
Ift22
Ift57
Ift74
Ift80
Ift88
Iglon5
Igsf9
Il12rb2
Il18
Il18r1
Il1rl1
Il20ra
Il4i1
Il9r
Immp1l
Immp2l
Immt
Impg1
Impg2
Inka1
Ino80e
Inpp1
Inpp4b
Inpp5e
Inpp5k
Insl6
Ints6
Ints7
Ip6k1

Ipo13
Ipo4
Iqank1
Iqca
Iqca1l
Iqcc
Iqcd
Iqcf1
Iqcf4
Iqcf5
Iqcf6
Iqcg
Iqch
Iqck
Iqcm
Iqcn
Iqgap1
Iqgap2
Iqub
Irak1bp1
Irf5
Irgc1
Isca2
Isg20l2
Isl1
Isoc2a
Itga10
Itgb3bp
Itgb6
Itih1
Itih5
Itpr2
Itprid1
Itprip
Itpripl1
Izumo1
Izumo2
Izumo3
Izumo4
Jakmip2
Jazf1
Jhy
Jmjd8
Josd1
Jph3
Jrk
Jrkl

Kansl1l
Kat7
Katnal2
Katnb1
Kbtbd3
Kbtbd7
Kcnab1
Kcnab2
Kcnc1
Kcnc2
Kcnh1
Kcnh8
Kcnip4
Kcnj16
Kcnj6
Kcnj9
Kcnk10
Kcnk2
Kcnma1
Kcnmb2
Kcnn2
Kcnu1
Kctd16
Kctd19
Kctd7
Kdm4d
Keap1
Khdrbs3
Kif17
Kif18b
Kif19a
Kif21b
Kif24
Kif26b
Kif27
Kif2a
Kif2b
Kif2c
Kif3a
Kif3b
Kif3c
Kif6
Kif9
Kifap3
Kifc3
Kin
Kiz

Klb
Klc3
Klf17
Klf4
Klf5
Klhdc1
Klhdc10
Klhdc3
Klhl12
Klhl28
Klhl3
Klhl41
Klhl6
Klhl7
Klk5
Klk8
Knstrn
Kpna4
Krba1
Krcc1
Krt1
Krt2
Krt71
Krt77
Krt88
Ktn1
Lacc1
Lactbl1
Lamp5
Lancl1
Lancl2
Lca5l
Lcorl
Lef1
Lekr1
Lemd1
Lepr
Letm2
Lexm
Lgals8
Lgi1
Lgr5
Lhx1
Lhx4
Lhx5
Lhx8
Lias

Lig3
Limd2
Limk2
Lin37
Lin52
Lin7a
Lin7b
Lin9
Lingo2
Lipe
Lipf
Liph
Lkaaear1
Llcfc1
Lmntd1
Lmntd2
Lmod3
Lnpk
Loxhd1
Lpcat2b
Lpin1
Lpin3
Lpp
Lpxn
Lrat
Lrguk
Lrif1
Lrit2
Lrp12
Lrp1b
Lrp2bp
Lrrc1
Lrrc10b
Lrrc18
Lrrc23
Lrrc26
Lrrc27
Lrrc28
Lrrc29
Lrrc3
Lrrc30
Lrrc34
Lrrc36
Lrrc3b
Lrrc43
Lrrc46
Lrrc49

Lrrc51
Lrrc52
Lrrc56
Lrrc57
Lrrc6
Lrrc61
Lrrc63
Lrrc66
Lrrc69
Lrrc71
Lrrc72
Lrrc73
Lrrc74a
Lrrc8b
Lrrc9
Lrrcc1
Lrrd1
Lrrfip2
Lrriq4
Lrrk1
Lrwd1
Lsm10
Lurap1
Luzp1
Ly6g6c
Ly75
Lyg2
Lypla1
Lyrm1
Lyzl1
Lyzl4
Lyzl6
Lztfl1
Lzts1
Maats1
Macrod2
Mad2l1bp
Maea
Mael
Magi2
Mak
Malrd1
Map2k2
Map3k10
Map7
Mapk10
Mapk15

Mapk8ip1
Mapk8ip2
Mapkap1
Mapre3
Marchf1
Marchf11
Marchf6
Mas1
Masp1
Mbtd1
Mcat
Mcidas
Mcm9
Mcmdc2
Mcoln2
Mcoln3
Mctp1
Mdc1
Mdga1
Mdga2
Mdh1b
Mdm1
Mdm2
Me3
Meak7
Mecr
Med1
Med26
Med27
Med7
Medag
Megf6
Meig1
Meikin
Meioc
Meis3
Memo1
Mesp1
Mettl22
Mex3d
Mfap3l
Mff
Mfsd11
Mfsd13b
Mfsd14a
Mfsd2b
Mfsd6l

Mgam
Mgat3
Mgat4c
Mgat4d
Mgat4e
Mgat4f
Mgll
Micos13
Mief1
Mif4gd
Miip
Mindy1
Mindy3
Mindy4
Mir125a
Mir135a-1
Mir1956
Mir429
Mkrn1
Mlc1
Mlf1
Mllt10
Mllt11
Mmel1
Mnd1
Mns1
Mob3b
Mog
Mogat1
Mok
Mon1a
Morc2a
Morc2b
Morn1
Morn2
Morn3
Morn5
Mphosph9
Mpig6b
Mpp6
Mppe1
Mreg
Mrgbp
Mrm3
Mrnip
Mroh2a
Mroh2b

Mroh3
Mroh4
Mroh5
Mroh7
Mroh8
Mroh9
Mrpl32
Mrpl58
Mrps22
Mrs2
Mrtfb
Ms4a1
Ms4a13
Ms4a14
Ms4a5
Msantd1
Msh4
Msl1
Msra
Mstn
Msto1
Mtbp
Mtch2
Mtf1
Mtfr1
Mtfr2
Mtif3
Mtmr12
Mtmr6
Mtmr9
Mtor
Mul1
Musk
Mxd1
Mybl1
Mybpc1
Mycbpap
Myh10
Myh13
Myh3
Myl1
Myl2
Mymx
Myo16
Myo18b
Myo19
Myo1a

Myo1c
Myo1h
Myo5b
Myog
Myom1
Myorg
Mypn
Myt1
Myt1l
N4bp2l2
Naa11
Naprt
Nasp
Nat9
Nbas
Nbea
Ncan
Ncaph
NCKIPSD
Ndc80
Ndfip2
Ndst4
Ndufa12
Ndufaf7
Neb
Nebl
Necab1
Necab3
Nectin3
Nek11
Nek2
Nek5
Nell2
Nemf
Nepn
Neurl1a
Nfasc
Nfatc2
Nfatc2ip
Nfkbie
Nfs1
Nfu1
Ngb
Ngef
Ngly1
Nhlh2
Nhlrc4

Nim1k
Nipsnap3a
Nkapl
Nkiras1
Nkx1-2
Nkx2-3
Nkx2-6
Nlgn1
Nlrp14
Nme5
Nme7
Nme8
Nmnat2
Nmt1
Nmu
Nol4
Nom1
Nos1ap
Noxred1
Npas4
Npepl1
Nphp1
Npm3
Npy2r
Nr1i2
Nr2e1
Nr6a1
Nrd1
Nrm
Nrsn1
Nrxn3
Nsd2
Nsmce1
Nsun4
Nsun7
Nt5c1a
Nt5c1b
Ntmt1
Ntrk3
Nudcd3
Nudt16l2
Nudt18
Nudt21
Nudt22
Nudt4
Nudt9
Numbl

Nup153
Nup155
Nup210l
Nup50
Nup54
Nup88
Nup98
Nupr1l
Nutm1
Nwd1
Nwd2
Nxt1
Oaz2
Oaz3
Oc90
Ocstamp
Odaph
Odf1
Odf2
Odf3
Odf3b
Odf3l1
Odf3l2
Oit1
Olfm4
Olfr1361
Olfr187
Olfr5
Olfr95
Onecut1
Optn
Orc3
Osbp
Osbp2
Osbpl10
Osbpl5
Oscp1
Oser1
Osgin1
Otof
Otub2
Otud7b
Ovol1
Oxgr1
Oxsm
P2rx3
Pabpc2

Pacrg
Pacs1
Padi3
Pak7
Palm3
Paqr3
Paqr5
Paqr9
Pard6a
Parp6
Parpbp
Pask
Pax2
Pax3
Pax4
Pax5
Pax6
Pbk
Pbx4
Pcbp3
Pcdh10
Pcdh15
Pcdha1
Pcdha3
Pcdhac2
Pcdhb1
Pcdhb13
Pcdhb14
Pcdhga11
Pcdhga5
Pcdhgb6
Pcf11
Pcgf6
Pclaf
Pcm1
Pcmt1
Pcnx
Pcsk1
Pcsk2
Pcsk4
Pdcd1lg2
Pdcd2l
Pdcl2
Pde11a
Pde1a
Pde3b
Pde4a

Pde4c
Pde4dip
Pde8a
Pdhb
Pdilt
Pdpk1
Pdzd8
Pdzd9
Pdzk1ip1
Pdzrn4
Peak1
Pemt
Pex11a
Pex11b
Pex11g
Pex12
Pex13
Pex3
Pfkp
Pfn3
Pfn4
Pgam2
Pgk2
Pglyrp3
Pgrmc2
Phactr3
Pheta2
Phf10
Phf14
Phf2
Phf21a
Phf7
Phkg2
Phlpp1
Phospho1
Phospho2
Phtf1
Phyhipl
Pias2
Picalm
Pick1
Pif1
Pifo
Pigm
Pigr
Pigyl
Pih1d2

Pik3ap1
Pinlyp
Pip5kl1
Pirt
Piwil1
Pja2
Pjvk
Pkd2l1
Pkd2l2
Pkib
Pkig
Pkmyt1
Pknox1
Pknox2
Pla2g2c
Pla2g4d
Pla2g6
Plaat1
Plaat5
Plac8l1
Plb1
Plbd1
Plcd4
Plce1
Plch2
Plcxd2
Plcz1
Plec
Plek
Plekha2
Plekha5
Plekhh2
Plekhm2
Plekho2
Plk1
Plk4
Pllp
Pln
Plpp1
Plpp6
Pmch
Pmfbp1
Pmis2
Pml
Pnldc1
Pnliprp1
Pnpla2

Poc1a
Poc1b
Podnl1
Pola2
Polb
Pold3
Polg
Poli
Polk
Poln
Polr2i
Polr3gl
Pom121l12
Pomt1
Pomt2
Popdc3
Pou2f1
Pou5f2
Pp2d1
Ppargc1a
Ppef1
Ppfia2
Ppil6
Ppm1b
Ppm1d
Ppm1e
Ppm1g
Ppm1j
Ppp1cc
Ppp1r10
Ppp1r11
Ppp1r17
Ppp1r1c
Ppp1r2
Ppp1r27
Ppp1r32
Ppp1r36
Ppp1r42
Ppp2r1b
Ppp2r2b
Ppp2r2d
Ppp2r3c
Ppp2r5c
Ppp2r5e
Ppp3cc
Ppp3r2
Ppp6c

Pram1
Prelid3b
Prim2
Prkaa1
Prkag2
Prkar1b
Prkar2a
Prkca
Prkcq
Prm2
Prm3
Prob1
Prok2
Prom1
Prop1
Prpf31
Prph2
Prpsap1
Prr11
Prr18
Prr19
Prr22
Prr27
Prr9
Prrx1
Prss21
Prss34
Prss37
Prss38
Prss39
Prss40
Prss42
Prss43
Prss44
Prss45
Prss46
Prss47
Prss52
Prss53
Prss54
Prss55
Prss58
Prune1
Prxl2a
Psd
Psd2
Psen1

Psip1
Psma8
Psmc3ip
Psmd13
Psme4
Psmf1
Psmg1
Pstpip2
Ptar1
Ptchd3
Ptdss2
Pten
Pth2
Ptpa
Ptpdc1
Ptpmt1
Ptpn20
Ptpn4
Ptprn2
Ptprt
Ptrh1
Pttg1
Pusl1
Pwwp3a
Pzp
Qrich1
Qrich2
R3hcc1
Rab11fip2
Rab11fip5
Rab28
Rab2b
Rab3b
Rab3c
Rab3gap2
Rab3il1
Rab4a
Rabac1
Rabepk
Rabgap1l
Rabl2
Rad18
Rad9b
Rag2
Ralgps1
Ralgps2
Ralyl

Ranbp17
Ranbp3l
Ranbp9
Rangap1
Rasal2
Rbm11
Rbm27
Rbm44
Rbm4b
Rbms3
Rcc1
Rchy1
Rcor3
Rcvrn
Rdh11
Rdh12
Rdh14
Rec8
Reep4
Reep6
Relt
Retreg1
Retreg2
Rftn2
Rfx1
Rfx2
Rfx3
Rfx4
Rfx8
Rgs16
Rgs22
Rgsl1
Rhbdd1
Rhbdd2
Rhbdd3
Rhcg
Rhno1
Rhot1
Rhov
Rhpn1
Ribc1
Ribc2
Rimbp3
Rin1
Rint1
Ripor3
Rmdn2

Rmi1
Rmnd5b
Rnase12
Rnaseh2a
Rnf126
Rnf138
Rnf139
Rnf144a
Rnf151
Rnf169
Rnf19b
Rnf20
Rnf32
Rnf38
Rnf40
Rnf41
Rnpep
Ropn1
Ropn1l
Rp1l1
Rp9
Rpgr
Rpgrip1l
Rph3a
Rpl39l
Rrp12
Rrp15
Rrp8
Rsbn1
Rsph1
Rsph10b
Rsph14
Rsph4a
Rsph6a
Rsph9
Rtkn2
Rtn4rl2
Runx1t1
Runx2
Ruvbl2
Rwdd2a
Rwdd4a
Rxfp1
Ryr3
S100a7a
Samd10
Samd13

Samd3
Samd4
Samd8
Sarm1
Satl1
Sav1
Saxo1
Saxo2
Saysd1
Sbds
Sccpdh
Scg5
Sclt1
Scly
Scmh1
Scml4
Scn11a
Scn4a
Scn9a
Scnn1a
Scoc
Scp2d1
Scrn3
Scrt1
Scrt2
Sdf2l1
Sec14l2
Sec14l4
Sec22a
Sel1l2
Selenos
Selenov
Sema4a
Senp2
Septin10
Septin12
Septin3
Septin4
Serp2
Setx
Sez6l
Sez6l2
Sfmbt1
Sftpd
Sgca
Sgcb
Sgip1

Sgk1
Sgms2
Sh2d2a
Sh2d6
Sh3gl3
Sh3glb1
Sh3rf2
Shank1
Shc3
Shcbp1l
Shisa3
Shisa7
Shkbp1
Shld2
Shmt1
Siah2
Siglec15
Sik2
Sik3
Sil1
Sirt2
Sirt4
Sit1
Sla2
Slain2
Slc11a1
Slc12a1
Slc12a3
Slc13a5
Slc14a2
Slc16a12
Slc16a3
Slc16a7
Slc17a2
Slc17a6
Slc1a2
Slc22a14
Slc22a16
Slc22a2
Slc22a23
Slc22a5
Slc22a7
Slc25a37
Slc25a39
Slc25a41
Slc25a54
Slc26a5

Slc26a8
Slc2a13
Slc2a3
Slc2a5
SLC30A3
Slc33a1
Slc34a2
Slc34a3
Slc35a5
Slc35b4
Slc35e3
Slc35f1
Slc35f4
Slc35g3
Slc36a3
Slc37a1
Slc38a11
Slc38a3
Slc38a9
Slc39a3
Slc44a5
Slc4a1ap
Slc4a5
Slc4a8
Slc5a12
Slc66a2
Slc6a20a
Slc6a20b
Slc6a6
Slc6a7
Slc7a9
Slc8a1
Slc8a2
Slc9a3r1
Slc9a4
Slc9b1
Slc9c1
Slco5a1
Slfn14
Slfn5
Slfnl1
Sltm
Smap1
Smc1b
Smc5
Smc6
Smco1

Smco2
Smcr8
Smg5
Smg7
Smg9
Smim23
Smpd2
Smpd3
Smpd5
Snap29
Sncaip
SNORA70
SNORD116
Snrpd1
Snta1
Sntg1
Sntn
Snupn
Snx19
Snx25
Snx31
Socs7
Sord
Sox30
Sox5
Sox6
Sp7
Spa17
Spaca1
Spaca3
Spaca4
Spaca7
Spaca9
Spag1
Spag16
Spag17
Spag4
Spag5
Spag6
Spag6l
Spag8
Spag9
Spam1
Spast
Spata16
Spata17
Spata18

Spata19
Spata20
Spata25
Spata3
Spata31
Spata31d1b
Spata32
Spata33
Spata4
Spata45
Spata46
Spata5
Spata7
Spata9
Spatc1
Spatc1l
Spats1
Spats2
Spcs1
Spdya
Spef1
Spef1l
Spem1
Spem2
Spesp1
Spg7
Spink2
Spink7
Spire2
Spo11
Spock3
Sprtn
Spryd7
Spsb2
Spta1
Srms
Srrm3
Srsf12
Ssmem1
Ssna1
Sstr2
Ssx2ip
St18
St6galnac2
St8sia6
Stam2
Stambp

Stard6
Stat4
Stil
Stk17b
Stk19
Stk31
Stk32b
Stk33
Stk39
Stkld1
Stmn3
Stom
Stox2
Stpg1
Stpg2
Stpg3
Stpg4
Strada
Strap
Strbp
Stt3b
Stx18
Stx2
Stx5a
Styxl1
Suclg1
Sufu
Sugp2
Sult1b1
Sult1c2
Sun1
Sun3
Sun5
Supt20
Supt3
Supt6
Supv3l1
Sv2b
Svip
Syce1l
Syce3
Syngr3
Syngr4
Synj2bp
Sypl
Syt14
Syt16

Syt5
Sytl3
Sytl5
T
Taar4
Taar9
Tacc3
Taf12
Tas1r1
Tasor2
Tatdn3
Tbata
Tbc1d1
Tbc1d14
Tbc1d15
Tbc1d2
Tbc1d20
Tbc1d21
Tbc1d23
Tbc1d2b
Tbc1d32
Tbc1d7
Tbc1d9
Tbkbp1
Tbl2
Tbp
Tbpl1
Tbx19
Tcam1
Tcea2
Tceanc2
Tcfl5
Tchp
Tcp11
Tcte1
Tctex1d1
Tctex1d2
Tctn1
Tdp2
Tdrd1
Tdrd3
Tdrd5
Tdrd6
Tdrd7
Tdrd9
Tek
Tekt1

Tekt2
Tekt3
Tekt4
Tekt5
Tenm2
Tenm4
Tent4b
Tent5b
Tent5c
Tepp
Terb2
Terf1
Tesk1
Tesk2
Tesmin
Tespa1
Tex13a
Tex13c1
Tex13c2
Tex13c3
Tex21
Tex22
Tex26
Tex28
Tex33
Tex35
Tex36
Tex37
Tex38
Tex43
Tex44
Tex45
Tex47
Tex48
Tex52
Tex55
Tex9
Tfam
Tfap2a
Tfap2b
Tfap2e
Tfb1m
Tfpi
Tg
TH
Thap2
Theg

Thegl
Themis3
Thnsl1
Thoc5
Thoc7
Thumpd3
Tigd2
Tigd4
Tigd5
Tigit
Tkfc
Tktl2
Tlk2
Tm6sf2
Tmbim7
Tmc1
Tmc5
Tmc7
Tmco2
Tmco5
Tmco5b
Tmem102
Tmem107
Tmem120a
Tmem129
Tmem132c
Tmem134
Tmem138
Tmem144
Tmem145
Tmem156
Tmem175
Tmem183a
Tmem190
Tmem191c
Tmem198b
Tmem200c
Tmem205
Tmem210
Tmem217
Tmem225
Tmem232
Tmem239
Tmem246
Tmem247
Tmem262
Tmem269

Tmem270
Tmem30c
Tmem39b
Tmem45b
Tmem50b
Tmem53
Tmem54
Tmem63c
Tmem67
Tmem82
TMEM89
Tmem95
Tmf1
Tmprss12
Tnfaip2
Tnfaip8l1
Tnfrsf11a
Tnks
Tnn
Tnnc1
Tnp1
Tnp2
Tnr
Tnrc6c
Tob1
Togaram1
Tom1
Tomm20l
Top2a
Topaz1
Toporsl
Tor1aip1
Tor1aip2
Tpcn2
Tpgs2
Tph2
Tpo
Tpp2
Tppp2
Tprgl
Tprn
Traf1
Traf3ip1
Traip
Tram2
Trh
Trhde

Trim11
Trim14
Trim17
Trim24
Trim27
Trim28
Trim29
Trim33
Trim36
Trim37
Trim42
Trim45
Trim46
Trim66
Trim69
Trim72
Trim80
Triml1
Trip12
Triqk
Troap
Trp53tg5
Trp63
Trpc6
Trpc7
Trpm8
Trpt1
Tsga10
Tsga13
Tshb
Tsks
Tsnaxip1
Tspan1
Tspoap1
Tspyl5
Tssk1
Tssk2
Tssk3
Tssk4
Tssk5
Tssk6
Ttbk1
Ttc12
Ttc21a
Ttc21b
Ttc22
Ttc23

Ttc25
Ttc26
Ttc29
Ttc30a2
Ttc30b
Ttc39a
Ttc39d
Ttc41
Ttc7
Ttk
Ttll1
Ttll10
Ttll11
Ttll13
Ttll3
Ttll6
Ttll8
Tuba4a
Tuba8
Tubal3
Tubb4b
Tubd1
Tulp2
Tulp4
Tusc3
Txndc8
Txndc9
Txnrd3
Tyk2
Uaca
Ubac1
Ubap2
Ube2c
Ube2f
Ube2j1
Ube2k
Ube3b
Ubl3
Ubl4b
Ubl7
Ublcp1
Ubn1
Ubn2
Ubox5
Ubqln3
Ubqln5
Ubr4

Ubr5
Ubtd1
Ubxn10
Ubxn11
Ubxn2a
Ubxn6
Ubxn8
Ucp3
Ufl1
Ufsp2
Uggt2
Uhrf1
Uhrf1bp1l
Ulk2
Ulk4
Unc13c
Unc45b
Unc5c
Unc80
Upk3a
Uqcc1
Uroc1
Usp1
Usp15
Usp16
Usp2
Usp25
Usp32
Usp37
Usp42
Usp44
Usp47
Usp48
Usp50
Usp8
Vash2
Vat1l
Vdac3
Vhl
Vinac1
Vipas39
Vmn2r1
Vps13a
Vps13b
Vps26a
Vps45
Vrk2

Vrk3
Vsig10l
Vsig8
Vwa3a
Vwa3b
Vwa5b1
Wapl
Wbp11
Wbp2nl
Wdfy1
Wdpcp
Wdr12
Wdr19
Wdr27
Wdr31
Wdr34
Wdr35
Wdr37
Wdr38
Wdr41
Wdr47
Wdr48
Wdr49
Wdr54
Wdr60
Wdr62
Wdr63
Wdr64
Wdr78
Wdr93
Wfdc15a
Wfdc3
Wnt10b
Wnt16
Wnt3
Wwp1
Xirp2
Xkr8
Xpo4
Xpo6
Xpot
Xpr1
Xrcc6
Xrra1
Ybx2
Ybx3
Ydjc

Yif1b
Ylpm1
Ypel1
Zan
Zbbx
Zbtb3
Zbtb32
Zbtb44
Zbtb48
Zbtb5
Zbtb8a
Zc2hc1a
Zc2hc1b
Zc2hc1c
Zc3h12c
Zc3h14
Zc3h3
Zcchc9
Zdbf2
Zdhhc11
Zdhhc19
Zdhhc23
Zdhhc4
Zdhhc5
Zfand3
Zfp1
Zfp157
Zfp174
Zfp185
Zfp217
Zfp267
Zfp286
Zfp287
Zfp35
Zfp365
Zfp367
Zfp37
Zfp385a
Zfp385c
Zfp420
Zfp438
Zfp451
Zfp462
Zfp512b
Zfp526
Zfp541
Zfp568

Zfp574
Zfp58
Zfp592
Zfp597
Zfp599
Zfp608
Zfp628
Zfp646
Zfp652
Zfp653
Zfp654
Zfp663
Zfp78
Zfp804a
Zfp821
Zfyve26
Zhx3
Zkscan17
Zkscan7
Zmiz2
Zmynd10
Zmynd12
Zmynd15
Znhit2
Znrd1as
Zp3r
Zpbp
Zpbp2
Zpr1
Zranb1
Zswim1
Zswim6
Zup1

W+ vs W- down
1110038F14Rik
1500009L16Rik
1700017B05Rik
2810459M11Rik
5730409E04Rik
A4galt
Aard
Abcd4
Abhd8
Abracl
Acaa2
Acadl
Acadm
Acads
Acbd4
Accs
Ackr4
Acot4
Acsf2
Acsl4
Acss1
Actc1
Actn1
Acy3
Adam33
Adamts2
Adamts7
Adcyap1
Adcyap1r1
Adgra3
Adgrb1
Adgrb2
Adgre5
Adgrg1
Adgrl1
Adi1
Adpgk
Adprhl2
Aebp1
Afm
Agap1
Aifm1
Ajuba
Ak3
Akap7

Akr1b3
Akr1c19
Akt1
Aldh16a1
Aldh1a1
Aldh1a3
Aldh2
Aldh3b1
Aldh4a1
Aldh7a1
Alox5ap
Alpk1
Als2cl
Amacr
Amh
Amhr2
Amigo2
Ampd3
Angptl2
Ank
Ank1
Ano8
Antxr2
Anxa1
Anxa11
Anxa2
Anxa4
Anxa6
Anxa7
Aox1
Apba3
Apbb1
Aph1a
Apobec3
Apoe
Apool
Aprt
Aqp1
Arhgap10
Arhgap22
Arhgef10l
Arhgef15
Arhgef19
Arhgef25
Arhgef3
Arhgef40
Arhgef5

Arid5a
Arl4c
Arl4d
Armcx3
Armcx6
Arpc1b
Arsb
Arsi
Arx
Asl
Asph
Atad3a
Atic
Atp13a2
Atp1a1
Atp1b2
Atp2a1
Atp5g2
Atp6ap1
Auts2
Avpi1
Axin2
Axl
B2m
B3gnt7
B4galnt4
B4galt4
Bace2
Baiap2l1
Bax
Bcam
Bckdhb
Bcl3
Bcl6
Bcl7a
Bcl9l
Bcr
Begain
Bex3
Bhlhb9
Bhlhe40
Bin1
Bmp1
Bmp4
Boc
Bok
Bphl

Btbd6
Btg2
C1qa
C1qb
C1qbp
C1qc
C1qtnf2
C1qtnf6
C1qtnf7
C7
Cables1
Cacnb3
Calhm5
Camk2n1
Camkk1
Camkk2
Capg
Car13
Caskin1
Casp1
Casp6
Cat
Cavin1
Cavin2
Cavin3
Cbr3
Cbx4
Ccdc102a
Ccdc17
Ccdc6
Ccdc8
Cckbr
Ccnd2
Cd151
Cd248
Cd276
Cd302
Cd3d
Cd44
Cd47
Cd53
Cd63
Cd68
Cd74
Cd99l2
Cdc25b
Cdc42ep1

Cdh24
Cdh3
Cdhr5
Cdkn1a
Cdon
Cebpb
Cebpd
Cenpb
Cers2
Chpf2
Chst11
Chst12
Chst2
Cited1
Cited2
Cited4
Ckb
Clcn2
Clcn4
Cldn11
Cldn3
Cldn5
Clec3b
Clip2
Cln3
Cln6
Clta
Clu
Clybl
Cmc4
Cmip
Cmtm8
Cndp2
Cnga2
Cnn2
Cnn3
Cnpy2
Cnrip1
Cntfr
Cntn3
Col18a1
Col1a2
Col27a1
Colec12
Colgalt1
Comt
Coq10a

Coro2b
Cotl1
Cox4i2
Cox7a2l
Cox7b
Cox8a
Cpe
Cpne2
Cpxm1
Creg1
Crispld2
Crtc2
Csad
Csdc2
Csk
Csnk1e
Ctdsp1
Ctsa
Ctsb
Ctss
Ctsz
Cttn
Cuedc1
Cul7
Cx3cl1
Cx3cr1
Cyba
Cyfip1
Cygb
Cyp11b1
Cyp11b2
Cyp21a1
Cyp27a1
Cyp2d22
Cyp4x1
Cyth4
D17H6S53E
D2hgdh
D630045J12Rik
Dao
Dcn
Dcps
Dctd
Dctpp1
Ddr1
Des
Dgkq

Dhrs13
Diras2
Dkk3
Dmtn
Dnase2a
Doc2b
Dok1
Dpysl2
Dpysl4
Dram2
Dsc2
Dse
Dtx4
Dusp7
Dusp9
Dynlrb1
E2f1
Ebp
Ece1
Edn1
Efcc1
Efemp2
Efna2
Efnb1
Efs
Egfl7
Egln3
Egr1
Egr4
Eid1
Eif2s3x
Elmsan1
Elovl7
Emilin1
Emp3
Enox2
Enpep
Entpd3
Epb41
Epdr1
Epha2
Ephb4
Ephx2
Epn3
Erap1
Erbb2
Etnk2

Ets2
Eva1b
Evc
Exoc3l2
Exosc6
Extl1
F11r
F2r
Fads1
Fads2
Fads3
Fads6
Fam107a
Fam129b
Fam131c
Fam13a
Fam160b2
Fam171a1
Fam180a
Fam20a
Fam20c
Fam3a
Fam3c
Fbln1
Fbxo16
Fbxo44
Fcer1g
Fcgrt
Fermt3
Fgd1
Fgf1
Fgf13
Fgf18
Fgfr1
Fgfr4
Fh1
Fhl1
Fibin
Fkbp10
Fkbp5
Fkbp9
Fli1
Flt3l
Folr2
Fos
Fosl2
Foxj2

Foxo4
Foxo6
Frzb
Fshr
Fst
Fundc1
Fundc2
Fuom
Fxyd1
Fxyd6
Fzd2
Gadd45b
Galnt6
Galnt9
Gamt
Gas6
Gas7
Gask1a
Gata4
Gata6
Gatm
Gba
Gbgt1
Gcdh
Gdnf
Gga2
Ghdc
Gimap1
Gja4
Glb1
Gli1
Glmp
Glo1
Glrb
Glrx
Gm12248
Gm20390
Gm27505
Gm27533
Gm28006
Gm45717
Gna14
Gng4
Gnl3l
Gpc1
Gpx1
Gpx3

Gpx7
Gpx8
Grhpr
Grip2
Grtp1
Gse1
Gsn
Gspt2
Gsta3
Gstm1
Gstm2
Gstm6
H2-Ea
H2-Eb1
H6pd
Hap1
Hdac7
Hdc
Hebp1
Hes1
Hes7
Hexb
Heyl
Hgfac
Hhatl
Hlcs
Hlf
Hmgb3
Hmgcs2
Hmox1
Hmx3
Hnrnpa1
Hoga1
Homer3
Hoxa9
Hoxc6
Hoxd4
Hoxd9
Hpcal1
Hps1
Hr
Hs3st1
Hs3st4
Hs6st1
Hs6st2
Hsf4
Hspa12b

Hspb2
Hspb8
Hyi
Icam4
Id3
IDH1
Idh2
Idh3g
Ier3
Ifitm3
Ifnar1
Ifngr1
Igbp1
Igdcc3
Igfbp6
Igfbp7
Ighm
Il17rc
Il18bp
Ilvbl
Impa2
Impdh1
Inf2
Inha
Inhbb
Inka2
Inppl1
Irak1
Irf2
Irf2bpl
Irf6
Irf7
Irgm2
Irs1
Irx4
Islr
Itga5
Itgb4
Itgb5
Itgb7
Itgb8
Itm2a
Itm2b
Itm2c
Jade2
Jdp2
Junb

Jund
Kank2
Kazald1
Kcna1
Kcnb1
Kcnc4
Kcnmb4
Kcnq1
Kcns1
Kcns3
Kctd15
Kctd17
Kdelr1
Kif1c
Kitl
Klf2
Klf6
Klhdc8a
Klhdc8b
Klhl13
Klhl21
Klhl26
Klhl40
L1td1
Lag3
Lama5
Lamb2
Lamc3
Laptm4b
Lbhd2
Lbp
Ldhb
Ldlr
Lfng
Lgals1
Lgals3
Lgals3bp
Lgi3
Limd1
Lipa
Lipg
Llgl1
Lmo4
Lmod1
Lpcat1
Lratd2
Lrp10

Lrp5
Lrrc17
Lrrn1
Lsp1
Lta4h
Ltbr
Luc7l3
Ly6e
Ly6h
Lzts2
Lzts3
Maf
Mafk
Maged1
Maged2
Mageh1
Man1c1
Map3k21
Map3k8
Map4k2
Mapk13
Mapkapk2
Mapkapk3
Mapre2
Mapt
Marcksl1
Marveld1
Mb21d2
Mbnl2
Mbnl3
Mbtps1
Mcam
Mcm2
Mcts1
Mef2c
Mef2d
Meis1
Meis2
Mertk
Mettl9
Mfap2
Mfge8
Mfsd10
Mgp
Mgst1
Mgst2
Mical1

Mid1ip1
Mirlet7b
Mkx
Mllt3
Mllt6
Mmab
Mmd
Mmd2
Mmp11
Mmp14
Mmp15
Mmp2
Mmp23
Mocs2
Morf4l2
Mpeg1
Mpp1
Mpp2
Mpv17l2
Mras
Mri1
Mro
Ms4a4a
Msantd3
Msantd4
Msi1
Msn
Mst1
Mt3
Mtap
Mtch1
Mtcp1
Mtss2
Mustn1
Mvb12b
Mvp
Mxra7
Mybpc3
Myc
Mycn
Myd88
Myh6
Myh7
Myh7b
Myh9
Mypop
Myrf

Myrip
Myzap
Naa10
Nacc2
Nadsyn1
Naglu
Nanos3
Nbl1
Ncs1
Ndfip1
Ndn
Ndp
Ndrg2
Ndufb11
Nectin1
Nedd9
Nek8
Nenf
Nfatc4
Nfe2l2
Nfkbia
Nhsl1
Nipsnap1
Nipsnap2
Nkap
Nkx1-1
Nlrx1
Nmrk1
Nol3
Noxa1
Npcd
Npdc1
Nptx1
Nr1d1
Nr2f2
Nr2f6
Nr5a1
Nrbp2
Nrep
Nrros
Nsmce4a
Nt5dc2
Ntn4
Oaf
Olfm1
Olfml1
Orai1

Osbpl7
Osr2
P2rx4
P2ry2
P3h3
P3h4
Padi2
Pak1
Pald1
Pam
Papss1
Pard3
Pard6b
Parm1
Parp16
Parva
Patz1
Pbx2
Pccb
Pced1b
Pcgf2
Pck2
Pcolce2
Pcyox1l
Pde9a
Pdgfrb
Pdha1
Pdlim3
Pdlim4
Pdyn
Pea15a
Pear1
Pecam1
Per2
Pex26
Pf4
Pfkfb3
Pgghg
Phka1
Phlda3
Piezo1
Pip4k2b
Pip5k1c
Pir
Pkdcc
Pkia
Pkn1

Plaat3
Plau
Pld2
Plekhg5
Plekhh1
Plekhh3
Plk2
Plod1
Plpp2
Plpp3
Plpp4
Plppr5
Plscr3
Plscr4
Pltp
Plxdc1
Plxna3
Plxnd1
Pmm1
Pmp22
Pmvk
Pnpla1
Poglut2
Postn
Ppic
Ppif
Ppm1m
Ppp1r14a
Ppp1r1b
Ppp2r1a
Ppp2r5b
Ppt2
Pqbp1
Prag1
Prdm11
Prdx3
Prelp
Prep
Prickle3
Prkcb
Proser2
Prr12
Prr7
Prss35
Psap
Psen2
Psma7

Psmb9
Psmd10
Pstk
Ptch1
Ptges2
Ptk7
Ptms
Ptp4a3
Ptpn3
Ptprf
Ptpru
Pvalb
Pycard
Pycr1
Pygb
Pygm
Pygo1
Pyroxd2
R3hdm4
Rab11fip4
Rab13
Rab34
Rab9
Rabggta
Rabggtb
Rac2
Rai1
Rapgef3
Rapgef4
Rara
Rarres2
Rasa3
Rasa4
Rasd1
Rasgrp2
Rasl10a
Rasl10b
Rasl12
Rbm10
Rbm3
Rbms2
Rbp1
Rcan2
Rcn1
Rcn3
Rcor2
Rcsd1

Reep5
Reg3g
Rem1
Ret
Rex1bd
Rftn1
Rgs10
Rgs11
Rhbdf1
Rhbdf2
Rhbdl3
Rhob
Rhoc
Rilp
Rilpl1
Rilpl2
Rnase4
Rnd3
Rnf130
Rorc
Rpp25
Rpp38
Rps17
Rps18
Rps3
Rps4x
Rps6ka4
Rps6kb2
Rps8
Rrad
Rras
Rras2
Rsad1
Rspo1
Rspo4
Rtkn
Rxra
S100a10
S100a13
S100a16
S100a6
Sall2
Sap30l
Sardh
Sat1
Sbk1
Scara3

Scara5
Scarb1
Scarb2
Scarf1
Scn1b
Sct
Sdc1
Sdc2
Sdc4
Sdsl
Sec24d
Selenon
Selenop
Sema3c
Sema3f
Sema3g
Sema4b
Sema4d
Sema7a
Serpine2
Serping1
Serpinh1
Sfrp1
Sfxn1
Sfxn2
Sfxn3
Sgce
Sh3bp4
Sh3d19
Sh3tc1
Sh3tc2
Shank3
Shbg
Shf
Shisa2
Shisa8
Ski
Slc12a9
Slc16a11
Slc16a13
Slc1a4
Slc22a17
Slc22a18
Slc25a10
Slc25a11
Slc25a13
Slc25a22

Slc25a23
Slc25a5
Slc27a3
Slc29a1
Slc29a2
Slc31a2
Slc35a4
Slc35c1
Slc35e4
Slc39a14
Slc39a7
Slc40a1
Slc41a1
Slc47a1
Slc48a1
Slc4a2
Slc4a3
Slc5a5
Slc6a9
Slc7a4
Slc7a5
Slc9a6
Smad3
Smad7
Smarcd3
Smim1
Smim10l2a
Smim20
Smo
Smtnl2
Smyd1
Snord38a
Snph
Sntb1
Snx12
Snx5
Socs3
Sod3
Sohlh1
Sox13
Sox3
Sox4
Sox8
Sox9
Sparc
Sparcl1
Speg

Sphk1
Spi1
Spint1
Spsb4
Sptb
Ssbp1
Ssbp3
Ssr4
Sstr3
St3gal2
St3gal3
St3gal4
St6gal2
St6galnac4
St6galnac6
Stab1
Stac3
Stambpl1
Stard3
Stat3
Stat5b
Stc2
Stk24
Stra6
Stradb
Stx1b
Stx4a
Sulf2
Sult5a1
Sumf1
Syde1
Syngr1
Synpo
Syt9
Sytl4
Tagln2
Tap1
Tap2
Tapbpl
Tax1bp3
Taz
Tbc1d9b
Tbl1x
Tbx2
Tcap
Tceal9
Tcf21

Tcf23
Tcf3
Tcf7l1
Tcf7l2
Tcirg1
Tep1
Tesc
Tfap4
Tfeb
Tgfbi
Tgm2
Tha1
Thbd
Thra
Thy1
Ticam1
Tie1
Timp2
Timp3
Tinagl1
Tjp3
Tkt
Tlnrd1
Tlr4
Tm7sf3
Tmc8
Tmcc2
Tmem132a
Tmem132e
Tmem14c
Tmem150a
Tmem151b
Tmem161a
Tmem176a
Tmem184b
Tmem240
Tmem256
Tmem47
Tmem63a
Tmem91
Tmem98
Tmsb4x
Tnfrsf12a
Tnfrsf1a
Tnfrsf25
Tnfsfm13
Tnip1

Tns2
Tnxb
Tomm40l
Tor4a
Tpbg
Tpd52
Tpm1
Tpm2
Tpm4
Tpp1
Tradd
Trib2
Trim2
Trim3
Trim47
Trim56
Trim8
Trip6
Trp53
Trpv4
Tsc22d4
Tsen34
Tshz1
Tsku
Tspan14
Tspan15
Tspan18
Tspan7
Tspyl2
Tsr2
Tst
Tstd1
Ttl
Ttll12
Ttyh1
Ttyh2
Ttyh3
Tubb6
Twf2
Twist1
Txnrd2
Tyro3
Uba7
Ubtf
Uchl1
Uckl1
Ucp2

Unc93b1
Usf1
Usf2
Ush1g
Usp36
Usp43
Utf1
Utp14a
Vamp8
Vasn
Vav1
Vegfa
Vegfb
Vim
Vopp1
Vps26b
Vps37d
Vwa1
Vxn
Wbp1
Wbp1l
Wdr13
Wdr45
Wdr86
Wnt2b
Wnt4
Wnt6
Wscd2
Wt1
Wwc1
Xaf1
Xylb
Xylt2
Yipf1
Ywhag
Zadh2
Zbtb12
Zbtb7c
Zcchc24
Zdhhc8
Zfp36
Zfp362
Zfp36l2
Zfp395
Zfp467
Zfp503
Zfp521

Zfp553
Zfp579
Zfp64
Zfp641
Zfp651
Zfp692
Zfp703
Zfp740
Zfp92
Zfp961
Zfpm1
Zhx2
Zmym3

W+ vs S up
Adamts2
Adamtsl2
Adgrl1
Amh
Aoc3
Arhgef3
Armcx4
Bcam
Calhm1
Calhm2
Cavin1
Ccl11
Cdon
Chp2
Col1a2
Col27a1
Ctsc
Cyp4v3
Dapk1
Diras2
Dsc2
Dse
Ednra
Efna2
Efnb1
Epb41l1
Fkbp9
Flna
Foxred2
Fstl1
Fzd7
Gas1
Gask1a
Hap1
Hs6st2
Il6ra
Ildr2
Itga1
Kcng1
Kcnj5
Kcnq1
Kdr
Lama2
Lama4
Lamb1

Lamb2
Lrp5
Lum
Maged2
Mapk13
Mmp15
Mt3
Myrf
Nav2
Ncs1
Nhs
Npcd
Npnt
Nrp1
Padi2
Pbx1
Pde8b
Pdgfd
Pdgfra
Pdlim3
Plekhg5
Prkcb
Ptpru
Rgmb
Scara3
Shisal1
Slc12a1
Slc1a3
Slc22a17
Slc48a1
Smtnl2
Snca
Sptb
Ston1
Stra6
Stxbp6
Tagln
Thra
Tmem121b
Trip6
Tshz1
Wnt4
Zc3h12a
Zfp46

W+ vs S down
1110017D15Rik
1700001K19Rik
1700001O22Rik
1700003F12Rik
1700009N14Rik
1700012B07Rik
1700015G11Rik
1700016C15Rik
1700016D06Rik
1700016H13Rik
1700017N19Rik
1700019D03Rik
1700019N19Rik
1700020A23Rik
1700029H14Rik
1700034E13Rik
1700037C18Rik
1700042G07Rik
1700074P13Rik
1700092M07Rik
1700093K21Rik
1700122O11Rik
2610318N02Rik
4430402I18Rik
4921507P07Rik
4921517D22Rik
4921539E11Rik
4930402F06Rik
4930404N11Rik
4930407I10Rik
4930505A04Rik
4930522H14Rik
4930544D05Rik
4930578I06Rik
4930590J08Rik
4931428F04Rik
4931429L15Rik
4933402J07Rik
4933402N03Rik
4933417A18Rik
5031439G07Rik
6330403K07Rik
Abhd16b
Ablim2
Ace

Acer2
Acrv1
Acsbg2
Acsbg3
Acsl1
Acte1
Actg2
Actl11
Actl7a
Actl9
Actrt1
Actrt2
Actrt3
Adig
Agpat2
AI661453
Aif1l
Aipl1
Ak1
Akap1
Akap3
Akap4
Aknad1
Ankef1
Ankrd53
Ankrd60
Ankrd61
Antxrl
Aqp7
Arl9
Arrdc5
Asb13
Asb17
Asb5
Atl3
Atoh8
Atp1a4
Atp6v1fnb
Atxn3
Azin2
Bag1
Bag3
Banf2
BC048562
BC048671
Bcan
Bcl2l14

Bmp8b
Bnc2
Bpifa3
Cabp2
Cabyr
Calhm3
Caln1
Calr3
Camk4
Cap2
Capza3
Cby2
Ccdc159
Ccdc169
Ccdc187
Ccdc192
ccdc198
Ccdc27
Ccdc33
Ccdc70
Ccdc91
Ccer1
Ccin
Cd164l2
Cd209e
Cd46
Cdc14a
Cdc14b
Cdh10
Cdhr3
Cdiptos
Cdk18
Cdk5r1
Cdkl5
Cdrt4
Cep295nl
Cep76
Cep85l
Cfap100
Cfap97d1
Chn2
Chpt1
Cldnd2
Clip4
Clmn
Clstn3
Clvs1

Cnbd2
Col22a1
Col26a1
Col5a1
Coq10b
Cpa5
Cpeb3
Cpn1
Cpvl
Crisp2
Crmp1
Csnk1g1
Csnk1g2
Cst10
Cst13
Cst8
Cstl1
Cthrc1
Cylc1
D7Ertd443e
Ddn
Dgkh
Dnah3
Dnajb4
Dnajb7
Dnajb8
DNAJC5B
Dnase1l3
Dpysl5
Dram1
Dynll2
Dyrk4
Ebf2
Efcab1
Efcab15
Efcab3
Efcab6
Efcab9
Egr3
Elavl4
Erich2
Erich3
Faim2
Fam107b
Fam110a
Fam122c
Fam166a

Fam166c
Fam183b
Fam187b
Fam205c
Fam209
Fam214b
Fam217a
Fam221b
Fam222a
Fam243
Fam71a
Fam71d
Fam71e1
Fam71f1
Fam71f2
Fam78a
Fam81b
Fancd2os
Fbxo24
Fbxo39
Fermt1
Fga
Fhdc1
Fhl5
Flywch2
Fndc8
Fscn3
Fstl5
Gabarapl1
Galntl5
Gapdhs
Gfod2
Gm11634
Gm136
Gm17359
Gm5460
Gm614
Gm6657
Gm7271
Gm9999
Gpat4
Gpd2
Gpi1
Gpr12
Gsg1
Gtsf1l
Gulo

Gxylt1
Habp2
Habp4
Hc
Hk1
Homez
Hook1
Hoxc9
Hs3st5
Hsdl2
Hsfy2
Hspa1l
Hyal5
Igsf9
Inpp1
Iqca1l
Iqcf1
Iqcf4
Iqcf5
Iqcf6
Iqcm
Iqcn
Irgc1
Isg20l2
Islr2
Itgb3bp
Itih5
Izumo1
Izumo2
Izumo3
Jph3
Kcnh8
Kif17
Kif21b
Kif27
Kif2b
Kif2c
Klc3
Klf17
Klk5
Lbh
Lemd1
Lexm
Lhx4
Lipe
Lipf
Lmtk3

Lnpk
Lpin1
Lpin3
Lpp
Lrrc18
Lrrc51
Lrrc52
Lrrc57
Lrrc69
Lrrc72
Lrrc74a
Lrrc8b
Lrrd1
Ly6g6c
Lypla1
Lyrm1
Mdga2
Mfap3l
Mgat3
Mgat4f
Mir125a
Mllt11
Mmel1
Mmp12
Morn3
Mpig6b
Mroh3
Mroh5
Mroh7
Ms4a14
Ms4a5
Msi2
Mstn
Mtfr1
Muc20
Myl2
Myo5b
NCKIPSD
Ndst4
Necab3
Neurl1a
Nfatc2
Nmur2
Nrap
Nrd1
Nrip2
Nsmf

Nsun4
Nt5c1b
Nuak1
Nudt16l2
Oaz3
Odf1
Odf3
Odf3b
Osbp2
Osgin1
Otub2
P4ha3
Paqr5
Pax4
Pdcd1lg2
Pde1a
Pdilt
Pdzd8
Pfn3
Phospho1
Picalm
Pim1
Pkib
Plcz1
Plek
Plekhh2
Plpp6
Pmfbp1
Pmis2
Poc1b
Pp2d1
Ppef1
Ppm1j
Ppp2r2b
Prkar1b
Prkar2a
Prkcd
Prkcq
Prm2
Prm3
Prph2
Prr27
Prss37
Prss52
Prss55
Prss58
Prune1

Psmf1
Pwwp2b
Rangap1
Rcc1
Reep6
Retreg1
Rgs22
Rmdn2
Rnf138
Rnf144a
Rnf151
Rnf169
Ropn1
Rtn4rl1
Runx2
Samd13
Samd4
Satl1
Scg5
Scp2d1
Scrt1
Septin4
Serpinb6b
Sgca
Sh3rf2
Slc11a1
Slc30a4
Slc36a3
Slc38a9
Smim23
Sox5
Sox6
Spaca3
Spaca9
Spag4
Spata18
Spata19
Spata20
Spata3
Spata31d1b
Spata32
Spata9
Spatc1
Spem1
Spem2
Srrm3
Sstr2

St6galnac2
Stimate
Stpg1
Stpg3
Stpg4
Sun3
Syngr3
Synpo2l
Tbata
Tbc1d20
Tbc1d21
Tekt5
Tent5c
Tepp
Tex13a
Tex33
Tex35
Tex36
Tex37
Tex43
Tex44
Tex45
Tex48
Tex52
Tex55
Tfam
Theg
Thegl
Thsd7b
Tigd5
Tmc5
Tmco2
Tmco5
Tmco5b
Tmem132c
Tmem190
Tmem191c
Tmem225
Tmem229b
Tmem239
Tmem247
Tmem255a
Tmem269
Tmem270
Tmem53
Tmem82
Tmprss11g

Tnp1
Tnp2
Tns1
Tppp2
Trim17
Trim36
Trim42
Trim66
Trim72
Trim80
Triml1
Trp53tg5
Trp63
Tsks
Tspan6
Tssk1
Tssk2
Tssk3
Tssk6
Ttc22
Ttc7
Ttll10
Tuba4a
Tuba8
Tubal3
Tulp2
Txlnb
Txndc8
Ube2k
Ubl3
Ubl4b
Ubqln3
Ubqln5
Ubxn11
Ubxn8
Unc5c
Uqcc1
Usp25
Usp3
Usp44
Usp50
Vinac1
Vsig10l
Vwa3a
Wbp2nl
Wdfy4
Wdr64

Wnt3
Ypel4
Zbbx
Zbtb8b
Zc2hc1b
Zfp365
Zic5
Zp3r
